# A fully orthogonal system for protein synthesis in bacterial cells

**DOI:** 10.1038/s41467-020-15756-1

**Published:** 2020-04-20

**Authors:** Nikolay A. Aleksashin, Teresa Szal, Anne E. d’Aquino, Michael C. Jewett, Nora Vázquez-Laslop, Alexander S. Mankin

**Affiliations:** 1 Center for Biomolecular Sciences, University of Illinois at Chicago, Chicago, IL 60607 USA; 2 Department of Pharmaceutical Sciences, University of Illinois at Chicago, Chicago, IL 60612 USA; 3 Interdisciplinary Biological Science Program, Northwestern University, 2145 Sheridan Rd, Evanston, IL 60208 USA; 4 Department of Chemical and Biological Engineering, Northwestern University, 2145 Sheridan Rd, Evanston, IL 60208 USA; 5 Center for Synthetic Biology, Northwestern University, 2145 Sheridan Rd, Evanston, IL 60208 USA

**Keywords:** Experimental organisms, Expression systems, Synthetic biology, Ribosome

## Abstract

Ribosome engineering is a powerful approach for expanding the catalytic potential of the protein synthesis apparatus. Due to the potential detriment the properties of the engineered ribosome may have on the cell, the designer ribosome needs to be functionally isolated from the translation machinery synthesizing cellular proteins. One solution to this problem was offered by Ribo-T, an engineered ribosome with tethered subunits which, while producing a desired protein, could be excluded from general translation. Here, we provide a conceptually different design of a cell with two orthogonal protein synthesis systems, where Ribo-T produces the proteome, while the dissociable ribosome is committed to the translation of a specific mRNA. The utility of this system is illustrated by generating a comprehensive collection of mutants with alterations at every rRNA nucleotide of the peptidyl transferase center and isolating gain-of-function variants that enable the ribosome to overcome the translation termination blockage imposed by an arrest peptide.

## Abstract (web summary)

Ribosome engineering is an emerging powerful approach for synthetic protein synthesis. Here the authors invert the Ribo-T system, using the engineered ribosome to translate the proteome while the native ribosome translates specific mRNA.

## Introduction

**Fig. 1:**
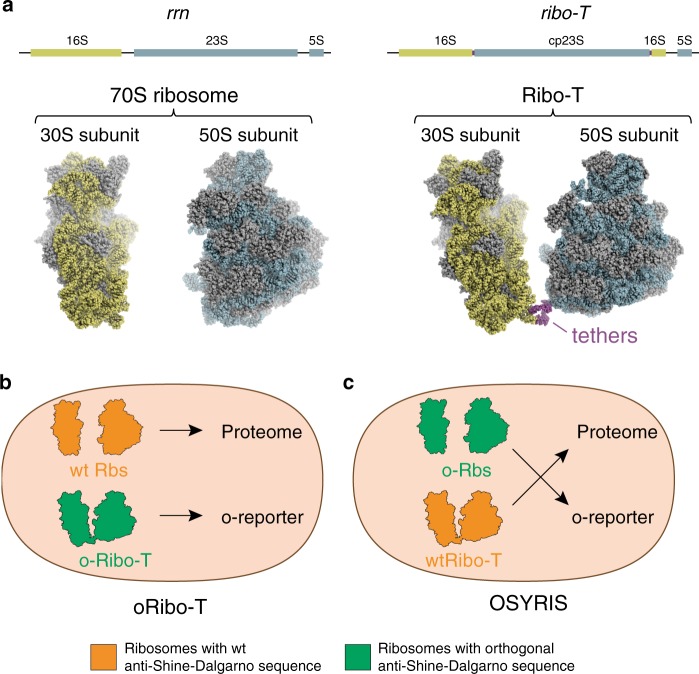
The OSYRIS set-up. **a** Organization of rRNA genes and structure of the dissociable 70S ribosome (left) and Ribo-T (right). The small and large subunits of Ribo-T are covalently linked by two RNA tethers connecting circularly-permutated 23S rRNA to the loop of helix 44 in 16S rRNA^14,17^. The sequences of 16S rRNA sequences are shown in olive and those of 23S rRNA in gray. **b** In the original Ribo-T-based orthogonal translation system^14^, wt dissociable ribosomes translate the cellular proteome while the orthogonal Ribo-T (oRibo-T) is committed to the translation of the orthogonal reporter (o-reporter) mRNA. **c** In the OSYRIS cells (this work), the proteome is synthesized by Ribo-T whereas the dissociable ribosomes function as a specialized orthogonal translation system. The tethered nature of Ribo-T confines both subunits of the dissociable ribosome (the 30S subunit with altered ASD and the 50S subunit) to the translation of only the o-reporter mRNA.

The ribosome performs distinct, complex, and highly coordinated functions during protein synthesis. It is composed of two subunits, small and large, which in bacteria are the 30S and 50S, respectively (Fig. 1a). The 30S subunit drives the initiation of translation relying in part on the complementarity between the Shine–Dalgarno (SD) sequence in the vicinity of the mRNA’s start codon and the anti-SD (ASD) sequence at the 3′ end of its 16S rRNA^1,2^. At the elongation stage of protein synthesis, the 30S subunit carries out the decoding function by sustaining codon–anticodon interactions, while at termination it facilitates the recognition of the stop codons by the release factors. The 50S subunit hosts the peptidyl transferase center (PTC) where polymerization of amino acids into a polypeptide takes place and also, at the termination phase, peptide release is catalyzed. The growing amino acid chain advances from the PTC into the nascent peptide exit tunnel (NPET) on its way out from the ribosome^3,4^.

The ribosome has evolved to operate with its natural substrates (mRNAs, tRNAs, and proteinogenic amino acids) enabling it to synthesize genetically encoded proteins. Nevertheless, its synthetic capabilities could be expanded by molecular engineering to allow the use of alternative genetic codes, polymerization of a wider variety of amino acids, or even carrying out a programmable synthesis of non-proteinaceous polymers^5^. Ribosome engineering could be also employed for elucidating the origin, evolution, and function of the protein synthesis apparatus. All such endeavors, however, require altering the intrinsic properties of the ribosome^6^ that inevitably diminish or even abolish the ribosome’s ability to synthesize cellular proteins^7,8^. Although interesting solutions to this problem could be offered by cell-free translation systems^9^, the efficiency and scalability issues limit their current application.

The ribosome engineering predicament can be overcome by creating an orthogonal protein synthesis apparatus within the cell that does not participate in the production of the cellular proteome and is exclusively dedicated to the translation of one or several specific mRNAs^10^. By mutating the ASD in the 16S rRNA and introducing a complementary SD sequence into an mRNA, it has been possible to direct a fraction of small subunits to the translation of only the cognate mRNA^11,12^, a strategy that has been exploited for expanding the decoding capacity of the ribosome^13^. The orthogonality of this set-up, however, is limited to only the small subunit because, due to the stochastic nature of the association of large and small ribosomal subunits in multiple rounds of translation, both the wild-type (wt) and the orthogonal 30S subunits share the same pool of 50S subunits. The inability to create orthogonal 50S subunits has limited the efforts to remodel the PTC and the NPET, the most critical sites for designing a translation apparatus with modified or expanded catalytic capacity. The engineering of the first fully orthogonal translation system became possible with the advent of the ribosome with tethered subunits, Ribo-T^14^. In Ribo-T and in subsequent similar designs^15–17^, circularly permutated 23S rRNA is embedded into the 16S rRNA, yielding a ribosome whose subunits are tethered by two RNA linkers (Fig. 1a). Because small and large subunits of Ribo-T are inseparable, in the orthogonal Ribo-T (oRibo-T) with altered ASD both subunits are committed to translating the cognate mRNA and thus, oRibo-T functions independently from the wt ribosomes that translate the cellular proteins (Fig. 1b). With the help of oRibo-T it was possible to select PTC mutations that facilitate the polymerization of specific amino acid sequences problematic for the wt ribosome^14,16^. However, while achieving full orthogonality, the unusual design of Ribo-T limits its functionality. Ribo-T translates proteins with only half the rate of the dissociable ribosome^14^ and it is more slow in departing from the start codons in comparison with the wt ribosomes^18^. Furthermore, the biogenesis of even non-orthogonal Ribo-T is rather slow and inefficient^18^ and the assembly problems could be further exacerbated if the ribosome’s functional centers are subjected to additional alterations^8^. All these factors complicate the direct use of Ribo-T in bioengineering efforts.

In order to overcome the shortcomings of the original oRibo-T-based approach, we have now designed a conceptually distinct system based on a bacterial cell with two functionally independent translation machineries, one of which is represented by dissociable, yet fully segregated ribosomes whose both subunits are dedicated to translation of only specialized mRNAs. By “flipping” the roles of Ribo-T and dissociable ribosomes, we engineered bacterial cells where translation of the proteome is carried out by Ribo-T, whereas the ribosome, composed of the dissociable orthogonal 30S (o-30S) subunit and wt 50S subunit functions as a fully orthogonal translation machine (Fig. 1c). In the resulting setup, that we named OSYRIS (Orthogonal translation SYstem based on Ribosomes with Isolated Subunits), complete orthogonality is achieved because the tethered nature of Ribo-T precludes it from associating with either the o-30S or the 50S of the dissociable ribosome. Therefore, in OSYRIS cells, the physically-unlinked o-30S and 50S ribosomal subunits are nevertheless compelled to interact with each other and function as fully orthogonal ribosomes (o-ribosomes). As a result, not only the o-30S, but also the free 50S subunit can be engineered to achieve new functionalities without interfering with the expression of the cellular proteome (Fig. 1c).

## Results

### Engineering OSYRIS

**Fig. 2:**
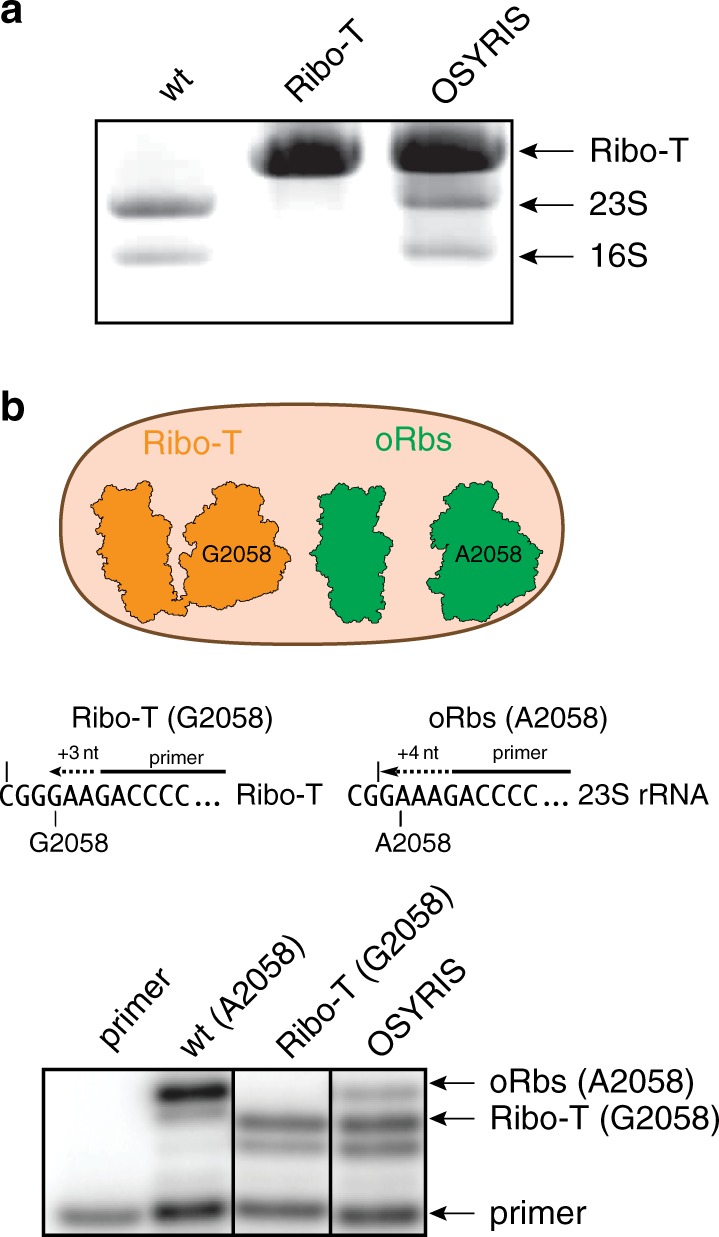
Performance of the dissociable orthogonal ribosome in the OSYRIS cells. **a** Gel electrophoresis analysis of the large rRNA species in the OSYRIS cells in comparison with wild type (wt) *E. coli*, containing only dissociable 70S ribosomes, and with Ribo-T cells (Ribo-T) which only carry tethered ribosomes. **b** Primer extension analysis of the rRNA content in the OSYRIS cells. Top, Ribo-T and the dissociable ribosome can be distinguished because of the A2058G mutation present in the Ribo-T rRNA. Middle, the principle of the primer extension analysis: in the presence of ddCTP, reverse transcriptase extends the primer by 4 nts on the 23S rRNA template (with A2058) but only by 3 nts on the Ribo-T rRNA template (with G2058). Bottom, gel electrophoresis analysis of the primer extension products generated on the rRNA extracted from wt, Ribo-T, or OSYRIS cells. The representative gels of three (panel **a**) or two (panel **b**) independent experiments are shown. Uncropped gel corresponding to panel **a** can be found in the Source data file.

The components of the system (Supplementary Fig. 1) were assembled in an *E. coli* strain that lacks chromosomal *rrn* alleles^19^ (Supplementary Fig. 2a, b). In the resulting OSYRIS cells, the Ribo-T rRNA with improved 16S-23S tethers^17^ is expressed from the optimized pRibo-Tt plasmid. Another plasmid, poRbs, carries the rRNA genes of the dissociable o-ribosomes, whose 16S rRNA gene has an altered ASD (Supplementary Fig. 1b). In the cells transformed with these two plasmids, the o-ribosomes account for ~15% of the total ribosomal population (Fig. 2a, b and Supplementary Fig. 3). Specialized reporter genes (*gfp*, *rfp*, *or luc*) with an SD cognate to that of the o-ribosome ASD (we will refer to these orthogonal reporters as o-reporters) are introduced on a third plasmid (poGFP, poRFP/oGFP, or poLuc) (Supplementary Fig. 1c–e).

**Fig. 3:**
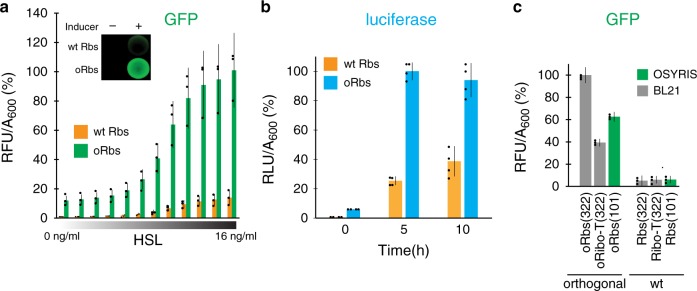
Expression of the orthogonal reporter in the OSYRIS cells. **a, b** Expression of o-reporters GFP (**a**) or luciferase (**b**) in OSYRIS cells carrying dissociable ribosomes with wt (wt Rbs) or orthogonal (oRbs) 30S subunit. Transcription of o-*gfp* was induced by varying concentrations of the inducer, homoserine lactone (HSL)^38^. Transcription of o-*luc* was induced by 1 ng/ml of HSL. The inset in (**a**) shows the UV light picture of the agar plate onto which the indicated cells were spotted and grown. **c** Comparison of the expression of the o-*gfp* reporter in OSYRIS cells expressing oRbs from a low copy number plasmid (green bars) with that in BL21 cells expressing oRbs or oRibo-T from a medium copy number plasmid (gray bars) (see Supplementary Fig. 1a, b). The medium-copy number plasmids are based on the pBR322 replication origin (322); the low-copy number plasmid is based on pSC101 replication origin (101). The graph bars represent the mean ± s.d. of 3 (**a**, **c**) or 4 (**b**) biological replicates. The raw data can be found in the Source data file.

The expression of the o-reporters in the OSYRIS cells relies on the o-ribosomes: in their absence, the reporter proteins (GFP, RFP, or luciferase) are produced at low levels, whereas the presence of the dissociable o-ribosomes, greatly stimulates their expression (Fig. 3a, b and Supplementary Fig. 4). Thus, o-30S subunits, whose operational exclusion from translation of cellular mRNAs has been confirmed in previous studies^12,17^, efficiently drive translation of o-mRNAs in OSYRIS. Noteworthy, dissociable o-ribosomes outperformed oRibo-T in expression of the o-reporters when introduced in the same host (*E. coli*, *BL21*) on the comparable vectors (Fig. 3c, Supplementary Figs. 5 and 6). Furthermore, relative expression of the o-GFP reporter in the OSYRIS cells, where o-ribosomes are expressed from a low-copy number plasmid, is higher in comparison with cells expressing oRibo-T from a higher-copy number plasmid (Fig. 3c, Supplementary Fig. 6).

### Dissociable ribosome does not translate cellular proteome

**Fig. 4:**
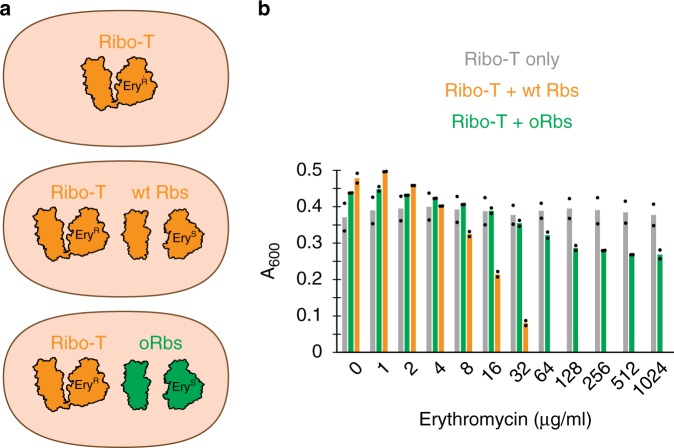
Resistance of the OSYRIS cells to erythromycin (Ery) illustrates the functional isolation of the orthogonal dissociable ribosome. **a** Ribosome composition of the OSYRIS cells expressing (orange) (wt Rbs)or orthogonal (green) (oRbs) ribosomes. Tethered ribosomes carry the A2058G mutation rendering them resistant to Ery (Ery^R^), whereas dissociable ribosomes are sensitive to Ery (Ery^S^). **b** Optical density (*A*_600_) of the OSYRIS cells cultures expressing wt Rbs or oRbs after 24 h growth in the 96-well plates in the presence of the indicated concentrations of Ery. Expressing Ery^S^ wt Rbs alongside Ery^R^ Ribo-T renders cells sensitive to Ery (orange bars) supporting the involvement of the 50S subunit upon its association wityh wt 30S subunit in proteome translation (MIC_Ery_ of these cells is 64 µg/mL). In contrast, cells expressing oRbs remain Ery^R^, demonstrating that the 50S subunit of the oRbs in the OSYRIS cells is functionally isolated and does not participate in the translation of the cellular proteome. The bar graph represents the mean of two biological replicates with individual data points indicated by black dots. The raw data can be found in the Source data file.

**Fig. 5:**
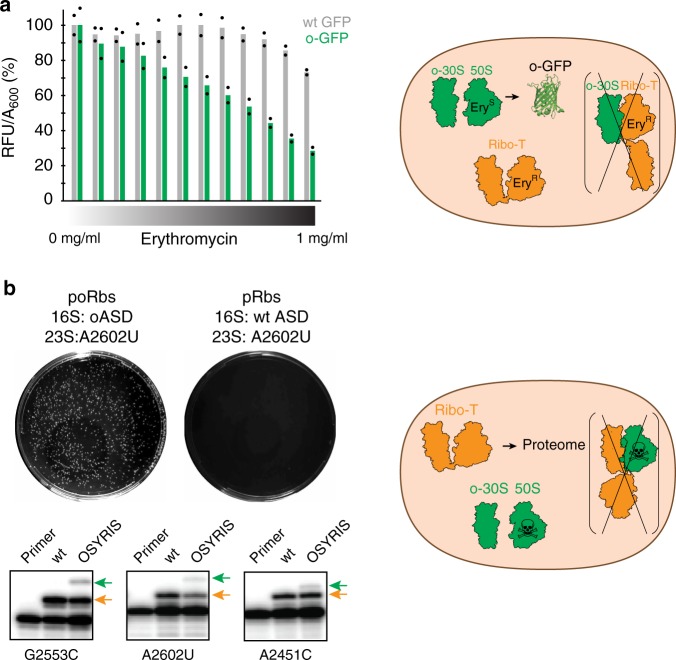
The orthogonality of the small and large subunits of the dissociable o-ribosome in the OSYRIS cells. **a** Expression of the o-GFP reporter in the OSYRIS cells is inhibited by erythromycin (left, green bars) demonstrating that it is translated primarily by the dissociable oRbs but not by the Ribo-T/30S hybrid (cartoon on the right). The bar graph represents the mean of two biological replicates with individual data points indicated by black dots. The raw data can be found in the Source data file. **b** Top: OSYRIS cells transformed with a poRbs plasmid with the lethal mutation A2602U are able to form colonies (left plate), revealing that the large subunit of the oRbs is excluded from the translation of the cellular proteome. The dominant lethal nature of the A2602U mutation in a non-orthogonal translation system is demonstrated by the lack of colonies when OSYRIS cells are transformed with the same plasmid but with unaltered (wt) ASD in the 16S rRNA gene (pRbs) (right plate) (see also Supplementary Fig. 7b, c). Bottom: primer extension analysis demonstrates that the OSYRIS cells stably maintain the large ribosomal subunits with 23S rRNA mutations that would be dominantly lethal in wt *E. coli* cells. cDNA bands representing mutant 23S rRNA (green arrows) or Ribo-T rRNA (orange arrows) are indicated. Co-existence of Ribo-T (with G2058) with dissociable ribosomes with lethal 23S rRNA mutations (but wt adenine at position 2058) was further confirmed by primer extension analysis around the 2058 rRNA residue (Supplementary Fig. 7d). Right: cartoon illustrating the conclusions from these experiments which argue that the dissociable 50S subunits are largely isolated from the translation of the cellular proteome whose expression relies on Ribo-T. The data represent the results of three independent biological replicates. The uncropped gel can be found in the Source data file.

To test whether both the small and large subunits of the dissociable o-ribosomes in the OSYRIS cells remain functionally isolated from Ribo-T, we took advantage of the A2058G mutation present in Ribo-T that renders it resistant to the antibiotic erythromycin (Ery)^14^. If the free 50S subunits, which are Ery-sensitive, could somehow cooperate with the small subunits of Ribo-T in translating the proteome, Ery would stall such hybrid ribosomes on mRNAs and thus inhibit general protein synthesis and cell growth. However, OSYRIS cells continue to grow even at the highest tested concentration of the antibiotic (1 mg/ml) (Fig. 4, green bars), demonstrating the functional autonomy of the dissociable 50S subunit and Ribo-T. In contrast, expression of the o-GFP reporter progressively decreased with the increase of Ery concentration in the medium (Fig. 5a). This result indicates that translation of the o-reporter is driven primarily by the ribosome composed of dissociable o-30S and 50S subunits, as opposed to o-30S/Ribo-T hybrids (Fig. 5a). Thus neither o-30S subunit, not 50S subunit interact with Ribo-T and both subunits remain functionally dedicated to each other in spite of the lack of a physical linkage between them.

A more rigorous proof of the orthogonality of the dissociable 50S subunits in the OSYRIS cells was obtained by introducing mutations into its 23S rRNA that are known to be dominantly lethal in wt *E. coli* cells^20,21^. Two of these mutations, A2451C and A2602U, alter critical nucleotides of the PTC active site, while mutation G2553C disrupts essential rRNA–tRNA interactions required for the proper placement of the A-site aminoacyl-tRNA for peptide bond formation^22,23^ (Supplementary Fig. 7a). If the mutant dissociable 50S subunits interact primarily with the o-30S subunits, survival of the OSYRIS cells should not be compromised because o-ribosome is excluded from general translation. If, on the contrary, the free 50S subunits associate with Ribo-T and participate in translation of the proteome, the dominantly lethal 23S rRNA mutations would prevent or severely compromise the growth of the OSYRIS cells. Attempts to express the mutant 50S subunits in the cells lacking o-30S (by transforming Ribo-T cells with the pRbs plasmid encoding the mutant 23S rRNAs along with wt 16S rRNA) yielded no transformants (Fig. 5b, Supplementary Fig. 7b, c), confirming the dominantly lethal nature of the 23S rRNA mutations. In contrast, when the mutant 23S rRNA gene was introduced in OSYRIS cells on the plasmid carrying orthogonal 16S rRNA, many transformants appeared (Fig. 5b, Supplementary Fig. 7b, c). Analysis of the rRNA isolated from the cultures of the transformed cells revealed fairly high expression level of the free 50S subunits containing the mutant 23S rRNA (Fig. 5b, Supplementary Fig. 7d). These results clearly demonstrate that the dissociable large ribosomal subunit remains functionally isolated from Ribo-T.

Altogether, the results of the o-reporter expression and tolerance to dominantly lethal mutations show that in the OSYRIS cells, the dissociable o-ribosomes translate o-mRNAs but do not significantly contribute to translation of the proteome. Therefore, both subunits of the dissociable o-ribosomes in OSYRIS cells are suitable for biomolecular engineering.

### Selecting gain-of-function mutations in OSYRIS cells

Having established the orthogonality of the dissociable ribosome in the OSYRIS cells, we carried out a proof-of-principle experiment to test the potential of the system for selecting mutations in the rRNA of the large subunit that would enable the ribosome to carry out otherwise problematic tasks. Specifically, we aimed to engineer a ribosome capable of efficient release of difficult-to-terminate proteins. In general, release of a fully synthesized polypeptide is a highly nuanced reaction catalyzed by the PTC with the assistance of class 1 release factors^24,25^. While most proteins are efficiently released at the stop codons, termination of others can be more troublesome^26,27^. An extreme case of inefficient termination in *E. coli* is represented by programmed translation arrest at the stop codon of the mRNA encoding the regulatory protein TnaC^28–30^. At high concentrations of tryptophan, the release of the fully translated TnaC is inhibited and the resulting stalling of the ribosome at the *tnaC* stop codon leads to the activation of the expression of the downstream genes of the *tna* operon^31^. The termination arrest at the *tnaC* stop codon is mediated by unfavorable interactions of the nascent TnaC with rRNA nucleotides of the NPET and the PTC^30,31^. The TnaC-mediated termination arrest represents a paradigm of inefficient protein release and illustrates one of the issues that could curb the expression of bioengineered polypeptides carrying, for example, non-canonical amino acids.

**Fig. 6:**
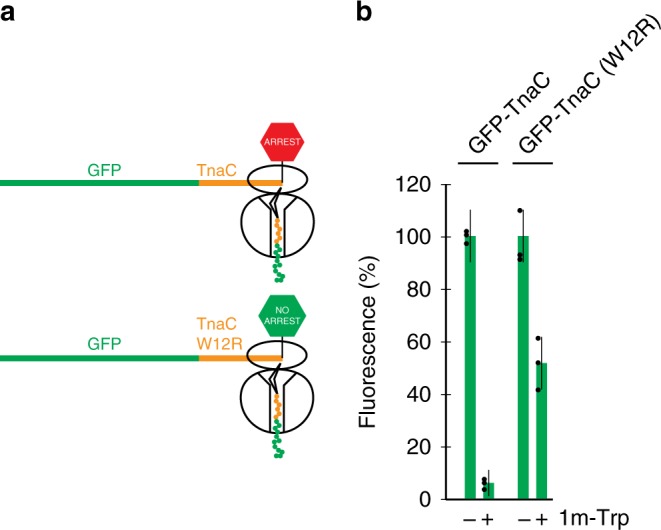
Engineering the reporter for selecting gain-of-function rRNA mutations. **a** Appending the TnaC-coding sequence to the end of *gfp* is expected to reduce the reporter expression due to the inhibitory action of TnaC on termination when translation occurs at high concentrations of l-tryptophan^28^. The presence of the W12R mutation in TnaC is known to partially alleviate the termination problem^28^ and should lead to a higher level of reporter expression. **b** Expression of the GFP-TnaC fusion in the OSYRIS cells is inhibited by 94% in the presence of the l-tryptophan analog 1-methyl tryptophan (1m-Trp), while the expression of the GFP-TnaC(W12R) mutant decreases only by 48%. The graph bars represent mean ± s.d. of three technical replicates. The raw data can be found in the Source data file.

In order to identify mutations that could alleviate the inefficient termination of TnaC, we constructed a reporter in which the TnaC-coding sequence (lacking its own start codon) was appended at the end of the *gfp* gene (Fig. 6a). As expected, in vivo and in vitro expression of the GFP-TnaC chimera was inhibited at high concentration of tryptophan (Fig. 6b, Supplementary Fig. 8). Introduction of the W12R mutation in the TnaC-coding segment, known to alleviate the termination arrest^28^, significantly stimulated the reporter expression in the presence of tryptophan in the cell and in cell-free translation system (Fig. 6b, Supplementary Fig. 8).

**Fig. 7:**
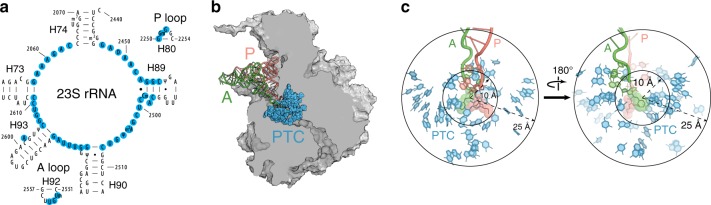
The PTC mutant library. **a** The 23S rRNA residues whose mutations comprise the PTC library shown in the secondary structure diagram of the 23S rRNA domain V central loop. The relevant 23S rRNA hairpins are indicated. **b** The location of the mutated PTC nucleotides in the 50S ribosomal subunit. The subunit is shown in a cross-cut representation. The P- and A-site tRNAs are shown. **c** The distance of the mutated 23S rRNA residues from the PTC active site. All the 23S rRNA residues within the 10 Å radius (the inner shell) and a large fraction of those within the 25 Å radius (second shell) of the PTC active site were mutated. The aminoacylated acceptor ends of the P- and A-site tRNAs are shown in pink and green, respectively.

We then generated a comprehensive library of 120 single-nucleotide 23S rRNA mutants in the poRbs plasmid (Fig. 7, Supplementary Fig. 1, Supplementary Table 1) which included alterations at: (i) every of the 9 rRNA residues in the PTC active site located within a 10 Å radius of the α-amino group of the acceptor amino acid participating in peptide bond formation; (ii) 41 second-shell nucleotides (those within a 25 Å radius from the PTC); (iii) 6 residues of the 23S rRNA P- and A-loops involved in positioning the acceptor ends of the P- and A-site tRNAs. Many of the individual mutations included in the library have been reported to be deleterious or lethal in wt *E. coli* cells^20,21^, and thus could be readily tested only due to the orthogonal nature of dissociable ribosomes in our system.

**Fig. 8:**
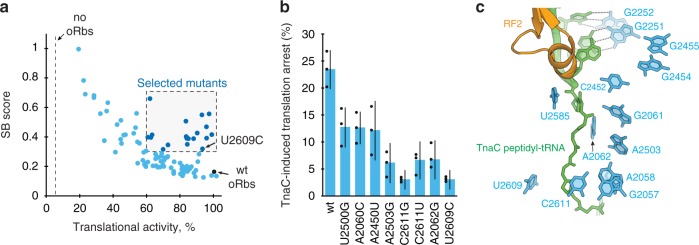
Selection of the gain-of-function mutations using OSYRIS. **a** Translational activity and stalling bypass (SB) score of the PTC library mutants expressing the orthogonal GFP-TnaC reporter in the OSYRIS cells. The dots representing the mutants exhibiting efficient termination (SB score > 0.3) while maintaining high efficiency of translation (>60% of the wt control) are shown in a darker shade of blue. The dotted line indicates the background level of expression of the orthogonal GFP-TnaC(W12R) mutant in the Ribo-T cells lacking oRbs. The black dot indicates the translation of the o-reporter by oRbs with wt 23S rRNA. **b** Testing ribosomes with gain-of-function mutations in a cell-free translation system (see Supplementary Fig. 11c). Ribosomal 50S subunits with lethal mutations (U2500G, A2060C, and A2450U) were isolated from OSYRIS cells and combined with wt 30S subunits. The ribosomes with non-lethal mutations were isolated from SQ171 cells (see Supplementary Table 2). The bar graph represents the mean ± s.d. of the three independent experiments. Statistical significance of the difference from wt values was determined by Student’s *t* test. **c** The placement of the 23S rRNA residues (blue) whose mutations resulted in gain-of-function (dark blue dots in panel **a**) relative to the TnaC-tRNA (green) and RF2 (orange) in the structure of the TnaC-stalled ribosome^30^.

We characterized the ability of the individual mutants to terminate translation of the GFP-TnaC polypeptide by estimating the stalling bypass (SB) score. The SB score reflects the relative expression of the hard-to-terminate GFP-TnaC reporter in comparison with the GFP-TnaC(W12R) variant that terminates efficiently (Fig. 6, Supplementary Fig. 8). The expression level of the GFP-TnaC(W12R) construct was also used to evaluate the effect of the PTC mutations on the general translation activity of the mutant ribosome. Strikingly, a number of the mutants with alterations in the PTC nucleotides exhibited a notably higher SB score than the OSYRIS cells with wild type 50S subunit (Fig. 8a, Supplementary Figs. 9 and 10). Among these, 19 mutants combined high translation activity (>60% of the wt control) with a significantly increased SB score (>0.3 vs. 0.17 for the wt control) (Fig. 8a, Supplementary Table 1). The identified mutations were at the 23S rRNA residues located in the PTC active site (G2061, C2452, and U2585), the P-loop (G2251 and G2252) and in the second PTC shell, including residues at the NPET entrance (A2057, A2058, C2611, A2062, A2503, and U2609) and two residues (G2454 and G2455) that via A2453 stack upon C2452 of the PTC (Fig. 8c). Two of the non-lethal mutations within this list (U2609C and A2058U) had been previously described as being capable of releasing TnaC-mediated termination arrest^29,31^ and therefore, served to validate that the newly identified mutations help to overcome ribosome stalling at the *tnaC* stop codon.

A unique opportunity offered by the OSYRIS cells is the possibility of isolating individual ribosomal subunits with even lethal mutations because dissociable 30S or 50S subunits can be separated from Ribo-T by sucrose gradient centrifugation^14^ (Supplementary Fig. 11a, b). Taking advantage of this feature of the system, we prepared large ribosomal subunits carrying lethal mutations U2500G, A2060C, and A2450U that showed SB score > 0.37, re-associated them with wt (non-orthogonal) 30S subunits, and tested in a cell-free translation system whether the mutations relieve ribosome stalling at the stop codon of the *tnaC* ORF (Supplementary Fig. 11c). We also tested some of the non-lethal mutants (A2503G, A2062G, C2611G, and C2611U) with SB scores of 0.35–0.55. Consistent with the in vivo data, all the tested mutant ribosomes showed decreased stalling at the *tnaC* stop codon in comparison with the wt ribosome during cell-free translation (Fig. 8b, Supplementary Fig. 11c), revealing their ability to more efficiently terminate translation of the TnaC peptide. The location of the identified termination arrest-releasing mutations suggests that either an altered placement of the peptidyl-tRNA or a less strict positioning of the P-site substrate and/or of the release factor in the PTC of the mutant ribosome facilitate TnaC release. Arguably, the mutations that relieve TnaC-mediated termination arrest could be possibly isolated using the previous oRibo-T-based approach^14,16^. However, some of the mutations identified in OSYRIS would likely be missed, because the reduced expression level of the reporter afforded by oRibo-T in comparison with dissociable o-ribosomes in OSYRIS (Fig. 3c) would limit the number of mutants exceeding the minimal efficiency threshold imposed in our screen.

## Discussion

Our proof-of-principle experiments demonstrated that the OSYRIS design, based on the ability of Ribo-T to sustain cellular growth while compelling the dissociable subunits of the o-ribosome to interact with each other, presents a conceptually distinct useful approach for generating a fully orthogonal cellular translation system. Engineering of OSYRIS was possible because Ribo-T is sufficiently active to translate the cellular proteome at a level required for sustaining cell growth^14^. However, translation driven by Ribo-T is sluggish and RiboT assembly is inefficient^18^, which likely is one of the factors that contributes to the slow growth rate of the OSYRIS cells (doubling time *τ* ~300 min in 96-well plates in comparison with *τ* ~45 min for the BL21 strain) (Supplementary Fig. 5a). Therefore, optimization of the Ribo-T functionality and assembly could improve the growth rate of OSYRIS cells and expand further the versatility of the orthogonal system. The three-plasmids set up (Supplementary Fig. 1) makes OSYRIS highly modular and, thus, easily adjustable for various applications. In principle, OSYRIS could be simplified further by introducing Ribo-T rRNA genes into the chromosome and combining the orthogonal rRNA genes and the reporter gene on the same plasmid. Reducing the number of plasmids could additionally facilitate growth of the OSYRIS cells. Increasing the fraction of o-ribosomes in the OSYRIS cells by modulating either the plasmid copy number or the promoter strength could be another way to improve the system performance and adjust it to specific needs. Thus, while in our experiments o-ribosomes were engaged in expression of only a single reporter gene, several genes equipped with altered SD could be translated simultaneously if the fraction of o-ribosomes is properly balanced, opening the possibility of orthogonal expression of, for example, multi-subunit protein complexes.

An obvious possible application of OSYRIS is engineering ribosomes capable of incorporation of non-canonical amino acids into polypeptides that the ribosome discriminates against (such as backbone modified D- and β-amino acids^32^). Although expanding the ribosome’s synthetic potential requires many components, from a specialized aminoacylation system to a designer genetic code, the fully orthogonal dissociable ribosome operating in the OSYRIS cells could accelerate the achievement of this goal. Importantly, OSYRIS makes possible many other endeavors, from employing ribosome retro-engineering for elucidating the origin of the translation apparatus to evolving new catalytic functions for programmable synthesis of polymers of nonprotein nature.

## Methods

### Plasmid construction

Plasmids used for generation and optimization of the OSYRIS set-up are shown in Supplementary Fig 1. The nucleotide sequences and features of the key plasmids are shown in Supplementary Data 1.

All the plasmids were constructed using Gibson assembly^33^, with the plasmid backbone prepared by inverse PCR or restriction nuclease digest and the cloned inserts either PCR-amplified from the respective templates or synthesized chemically by Integrated DNA Technologies. PCR reactions were carried out using Q5 high-fidelity DNA polymerase (New England Biolabs), and PCR products were purified using DNA Clean and Concentrator kit (Zymo Research). The Gibson assembly reactions for rRNA-encoding plasmids were electroporated into *E. coli* POP2136 cells (all the bacterial strains are listed in Supplementary Table 2) and transformants were recovered on LB plates supplemented with the proper antibiotics; plates were incubated at 30 °C to prevent the expression of the rRNA genes controlled by the lambda P_L_ promoter^34^. All the other plasmids were transformed and propagated in *E. coli* JM109 strain grown in LB media supplemented when needed with 100 μg/ml of ampicillin (Amp), 50 μg/ml of kanamycin (Kan), or 50 μg/ml of spectinomycin (Spc). Plasmids were isolated using High Pure Plasmid Isolation Kit (Roche), checked by PCR and capillary sequencing, and used for engineering of the OSYRIS cells. The following sections outline the construction of the main plasmids.

### Constructing the pRibo-Tt plasmid

The backbone of the pRibo-T v 2.0 plasmid^17^, carrying the A2058G erythromycin resistance mutation, was linearized with SgsI restriction enzyme and purified. The cluster of the missing tRNAs genes (encoding tRNA^Glu^, tRNA^Ala^, tRNA^Ile^, tRNA^Trp^, and tRNA^Asp^), whose transcription is controlled by the P_tac_ promoter and T1 terminator, was synthesized as a gBlock (Integrated DNA Technology) and PCR-amplified using primers NA1 and NA2 (all primers are listed in Supplementary Table 3). PCR reaction was catalyzed by the Q5 High-Fidelity DNA Polymerase (New England Biolabs) according to the manufacture protocol under the following conditions: 98 °C, 30 s followed by 30 cycles (98 °C, 10 s; 64 °C, 30 s; 72 °C, 20 s), followed by the final incubation for 2 min at 72 °C. Purified PCR product (20 ng) was mixed with SgsI-linearized pRibo-T v.2.0 backbone (80 ng) in a Gibson assembly reaction (1.7% PEG-800, 3.1 mM DTT, 0.31 mM β-nicotinamide adenine dinucleotide, 62.5 µM each dNTP, 3.1 mM MgCl_2_, 31.3 mM Tris/HCl, pH 7.5, 0.004 U/µl T5 Exonuclease (Epicenter), 4 U/µl Taq DNA Ligase (New England Biolabs), 0.025 U/µl Phusion High Fidelity DNA Polymerase (New England Biolabs). After 1 h incubation at 50 °C, 3 µl of the reaction mixture were transformed into electrocompetent POP2136 *E. coli* cells. Cells were plated onto LB/Amp agar plates. Individual colonies of the transformants were picked, grown onto LB/Amp media and plasmids were isolated. The presence of the tRNA cluster was confirmed by PCR amplification using primers NA3 and NA4 and sequencing.

### Constructing the poRBS plasmid

Orthogonal and wt rRNA operons under transcriptional control of the lambda P_L_ promoter and T1/T2 terminators were PCR amplified from the pO2 and pAM552 plasmids^14^, respectively, using the primers NA5 and NA6. The Kan^R^ gene was amplified from the plasmid pKD13^35^ using the primers NA7 and NA8. pSC101 origin of replication was amplified from the pCSacB plasmid^36^ using the primers NA9 and NA10. The PCR reactions were treated with DpnI to reduce the background of the parental plasmids. The PCR products were purified, confirmed by electrophoresis, and mixed (40 ng of each) in the Gibson assembly reaction. After 1 h incubation at 50 °C, 3 µl of the reaction mix were transformed into electrocompetent POP2136 *E. coli* cells. Cells were plated onto LB/Kan agar plates. After 24 h incubation at 30 °C, individual colonies were picked, grown in LB/Kan media, and plasmids were isolated and verified by restriction digest and sequencing.

### Constructing the poGFP plasmid

The o-GFP gene with 5′UTR, 3′UTR, and T1/T2 terminators was PCR amplified from the plpp5-oGFP plasmid^14^ using primers NA11 and NA12. The LuxR repressor and the P_Lux_ promoter^37^ were PCR amplified from the pJDO75 plasmid^38^ using primers NA13 and NA14. Spc^R^ marker (*aadA*) was PCR amplified from the ptRNA67 plasmid^36^ using primers NA15 and NA16. The p15A origin of replication was PCR amplified from the ptRNA67 plasmid using primers NA17 and NA18. The PCR reactions involving plasmid templates were treated with DpnI. Purified PCR products (40 ng of each) were mixed in the Gibson assembly reaction. After 1 h incubation at 50 °C 3 µl of the reaction mix were transformed into electrocompetent JM109 *E. coli* cells (Promega). Cells were plated onto LB/Spc agar plates. After 18 h incubation at 37 °C, individual colonies were picked, grown in LB/Spc media and plasmids were isolated. The presence of the *luxR* gene insert was confirmed by PCR using primers NA19 and NA20. Restriction digest of the resulting plasmid indicated that its size exceeds the expected one by ~1 kb. Subsequent restriction analysis and sequencing showed that the *luxR* gene has undergone duplication (Supplementary Fig. 1c). This duplication is not expected to affect the o-*gfp* reporter expression.

### Constructing the poRFP-oGFP plasmid

The Spc^R^ marker (*aadA*) and the p15A origin of replication were PCR amplified from the ptRNA67 plasmid^36^. The PCR reactions were treated with DpnI. The o-GFP gene with P_lpp5_ promoter, 5′UTR, 3′UTR, and T1/T2 terminators was PCR amplified from the plpp5-oGFP plasmid^14^. Purified PCR products (~40 ng of each) were mixed in the Gibson assembly reaction. After 1 h incubation at 50 °C, 3 µl of the reaction mix were transformed into electrocompetent JM109 *E. coli* cells (Promega). Cells were plated onto LB/Spc agar plates. After 24 h incubation at 37 °C, individual colonies were picked, grown in LB/Spc media and plasmids were isolated. The structure of the resulting plasmid plpp5-oGFP-pA15-Spec was verified by restriction digest and sequencing. The *rfp* gene with P_T5_ promoter and T0 transcription terminator was PCR-amplified from the plasmid pRYG^39^, the orthogonal SD sequence was introduced by PCR and the resulting *o-rfp* construct was inserted into a unique SphI site of the plpp5-oGFP-pA15-Spc plasmid.

### Constructing the poLuc plasmid

The plasmid poLuc carrying the orthogonal luciferase gene was constructed based on poGFP (Supplementary Fig. 1c). The 1653 bp gene *luc* encoding firefly luciferase was PCR amplified from the pBESTluc plasmid (Promega) using the primers NA21 and NA22. The resulting PCR product and the poGFP plasmid were cut with restriction enzymes BglII and SalI and ligated. The ligation mixture was transformed into *E. coli* JM109 competent cells, the *luc* gene-positive clones were identified by colony PCR, and the integrity of the cloned *luc* gene was verified by sequencing.

### Constructing the poGFP-TnaC plasmid

For constructing the reporter poGFP-TnaC plasmids (wt or W12R mutant), the *gfp*-coding sequence in the poGFP plasmid was replaced with the sequences coding for the chimeric wt or mutant GFP-TnaC proteins. The DNA inserts containing the orthogonal ribosome binding site and GFP-TnaC or GFP-TnaC (W12R) coding sequences were generated by PCR using the templates used for in vitro translation (described below) using primers NA23 and NA24. After purification, the inserts were introduced by Gibson assembly into the poGFP plasmid cut with the restriction enzymes BglII and SalI. After transformation, the presence of the correct insert in individual colonies was checked by colony PCR using the primers NA25 and NA26 and by sequencing the corresponding segments of the plasmid.

### Engineering Ribo-T-expressing cells

SQ171 FG cells (Supplementary Table 2) that lack chromosomal rRNA alleles^19^ and carry mutations in the *ybeX* and *rpsA* genes that stimulate their growth when expressing Ribo-T^14^ were used as the host (Supplementary Fig. 2). The gene *upp* was inactivated by recombineering for the future possible use of 5-fluorouracil negative selection.

The recipient cells initially carried two plasmids: the pCSacB plasmid containing the *rrnB* operon, counter-selectable *sacB* marker, and Kan^R^ gene, and the ptRNA67 plasmid carrying the missing tRNA genes that were eliminated during deletion of the chromosomal rRNA operons^36^. Cells were made electrocompetent and then 50 µl of the cell suspension were transformed with 50 ng of the pRibo-Tt plasmid, carrying the Ribo-T rRNA genes and missing tRNA genes (Supplementary Fig. 1), isolated from the POP2136 cells. Transformed cells were diluted with 1 ml of SOC medium (2% tryptone, 0.5% yeast extract, 10 mM NaCl, 10 mM MgSO_4_, 10 mM MgCl_2_, 20 mM glucose) and incubated at 37 °C for 6 h with shaking. A 150 μl aliquot of the culture was diluted to 2 ml with fresh SOC medium supplemented with 50 μg/ml Amp, 25 μg/ml Spc, and 0.25% sucrose, and grown for 12 h at 37 °C with constant shaking. Cells were spun down (1 min, 5000*g*) and plated on LB/agar plates containing 50 μg/ml Amp, 25 μg/ml Spc, 5% sucrose, and 1 mg/ml erythromycin (Ery). Plates were incubated for 48 h at 37 °C. The absence of the pCSacB plasmid was verified by the sensitivity of the transformants to Kan that was tested by replica plating colonies on LB/agar plates supplemented with 50 μg/ml Amp, 25 μg/ml Spc with or without the addition of 50 µg/ml of Kan. Transformants were then grown in LB media supplemented with 50 μg/ml Amp and 25 μg/ml Spc, plasmids were isolated and verified by restriction analysis. The absence of the wt rRNA was additionally confirmed by isolation of the total RNA using the RNeasy Mini Kit (Qiagen) and agarose gel electrophoresis.

### Elimination of the ptRNA67 plasmid

The obtained transformants were then cured of the ptRNA67 plasmid. For that, the cells were passaged in LB media supplemented with 100 μg/ml Amp for ~100 generations. After plating cell dilutions, the absence of the ptRNA67 plasmid in individual clones was verified by their sensitivity to Spc and the lack of visible amounts of the ptRNA67 plasmid bands in the restriction digest of the total plasmid preparation.

### Inactivating the recA gene in the Ribo-T-expressing cells

Our initial attempts to introduce poRbs into engineered cells frequently led to the appearance of the aberrant plasmids resulting from recombination between the poRbs and pRibo-Tt plasmids. Therefore, to avoid this problem, we inactivated the *recA* gene in the cells bearing the pRibo-Tt plasmid. (Of note, inactivating the *recA* gene before curing off the ptRNA67 plasmid prevented the plasmid loss even after prolonged passaging of the cells in the absence of Spc).

To inactivate the *recA* gene in the OSYRIS cells by P1 phage transduction, we first prepared the donor strain BW25113 *recA::cat* by the conventional recombineering procedure using chloramphenicol (Chl)-resistance cassette from the pKD3 plasmid^35^. The cassette was PCR-amplified using the primers NA27 and NA28. PCR fragment was transformed into BW25113 strain carrying the Red recombinase-expressing plasmid pDK46. After the selection and verification of the *recA::cat* strain, and curing the pKD46 plasmid, the resulting strain was used as a donor for the phage transduction. P1 phages transduction was carried out according to the standard protocol^40^ except that the recovery incubation was 6 h instead of 1 h before plating the transductants on LB/agar plates supplemented with 50 μg/ml Amp and 15 μg/ml Chl. The genotype of the engineered strain is shown in Supplementary Table 3.

### Introducing the poRbs plasmid into SQ171/pRibo-Tt cells

The SQ171 FG *ΔrecA*/pRibo-Tt strain was then transformed with the poRbs (or when needed, pRbs) plasmid by electroporation and selection of the Amp^R^/Kan^R^/Chl^R^ cells. The only deviation from the standard transformation protocol was that recovery of the transformants in the SOC medium lacking antibiotics was prolonged to 6 h prior and transformants were selected on LB/agar plates supplemented with 50 μg/ml Amp, 25 μg/ml Kan, and 15 μg/ml Chl. Transformants were verified by restriction analysis of the total plasmid and analysis of rRNA by agarose gel electrophoresis.

### Introducing the reporter plasmids

Reporter plasmids (poGFP, poRFP/oGFP, poLuc, and poGFP-TnaC) were introduced by electroporation into SQ171 FG *ΔrecA*/pRibo-Tt/poRbs cells and selection of the Amp^r^/Kan^r^/Chl^r^/Spc^r^ cells, essentially as described in the previous section.

### Verifying the genome sequence of the OSYRIS cells

During the construction of the OSYRIS cells, the original host cells have been passaged multiple times and undergone single-colony purification at multiple steps, possibly leading to the accumulation of spontaneous mutations. Therefore, the total genome of the fully assembled OSYRIS cells was sequenced. Analysis of the resulting sequence showed the presence of mutations in several genes (Supplementary Table 4). Some of these mutations (e.g., in the genes *ptsI* or *ackA*) may potentially negatively affect cell growth under some conditions and could be corrected in the future by genome engineering.

### Analyzing expression of the orthogonal gfp gene

The OSYRIS cells carrying either poRbs or pRbs plasmids (expressing orthogonal or non-orthogonal ribosomes, respectively) and the poGFP reporter plasmid were grown overnight in LB media supplemented with 50 μg/ml Amp, 25 μg/ml Kan, 25 μg/ml Spc, and 15 μg/ml Chl at 37 °C with constant shaking. Cultures were diluted 1:40 (v/v) in fresh LB media supplemented with the same antibiotics and additionally containing 1 ng/ml of N-(β-ketocaproyl)-l-homoserine lactone (HSL) (Santa Cruz Biotechnology), the inducer of the reporter gene transcription. The cultures (120 μl) were placed in the wells of the 96-well flat-bottom polystyrene tissue culture plate (Costar) and placed in the plate reader (TECAN Infinite M200 Pro) and incubated at 37 °C with constant linear (3 mm) shaking. Cell culture densities (*A*_600_) and GFP fluorescence (an excitation wavelength of 485 nm, an emission wavelength of 520 nm, optimal gain 30% RFU with applying the gain regulation function) were monitored over a time period of 24–48 h. The autofluorescence of cells lacking the reporter was subtracted from all the recorded values.

For the erythromycin sensitivity test, overnight cultures were diluted 1:40 into fresh LB media supplemented with either only HSL (final concentrations: 0–16 ng/ml) or with 1 ng/ml of HSL and varying concentrations of erythromycin (final concentrations: 0–1 mg/ml). Monitoring of cell growth and GFP expression was as described in the previous paragraph.

When OSYRIS cells carried the poRFP/poGFP reporter, the expression of RFP was monitored using an excitation wavelength of 550 nm and an emission wavelength of 675 nm, optimal gain 30% RFU with applying the gain regulation function.

### Analyzing expression of the orthogonal luciferase gene

The OSYRIS cells carrying the poLuc plasmid were grown for 24 h in LB media supplemented with 50 μg/ml Amp, 25 μg/ml Kan, 25 μg/ml Spc, and 15 μg/ml Chl and then diluted 1:40 into fresh medium containing the same antibiotics and 1 ng/ml of HSL. After 6 h, 0.2 *A*_600_ of each culture was spun down (5 min, 5000*g*, 4 °C), and cell pellets were flash-frozen. Luciferase activity was measured using the Luciferase Assay System (Promega) following the manufacturer’s protocol. Specifically: cell pellets were thawed in a 20 °C water bath and resuspended in a 25 µl of LB supplemented with 10% (v/v) of dibasic phosphate buffer (1 M K_2_HPO_4_ pH 7.8, 20 mM EDTA). Twenty microlitre of cell suspension were mixed with 60 μl of freshly prepared lysis mix (25 mM Tris-phosphate pH 7.8, 2 mM dithiothreitol, 2 mM 1,2-diaminocyclohexane-N,N,N′,N′-tetraacetic acid, 10% glycerol, 1% Triton X-100, 1.25 mg/ml lysozyme, 2.5 mg/ml bovine serum albumin), and lysed at room temperature for 10 min. The 10 µl aliquots of cell lysates were then placed into wells of 96-well black/clear bottom assay plate (Corning), 50 µl of Luciferase Assay Reagent (Promega) were added and fluorescence readings were immediately acquired in TECAN microplate reader.

### Comparing the expression of the reporters by oRibo-T or oRbs

*E. coli* BL21 strain was transformed with either poGFP or poLuc plasmids. The transformants were selected on LB/agar plates supplemented with 50 µg/ml of Spc, grown from individual colonies, and then rendered electrocompetent. The reporter-containing cells were then transformed with poRibo-T (the pBR322 ori-based, Amp^R^ plasmid expressing oRibo-T rRNA)^17^, or with o-pAM552 plasmid (the pBR322 ori-based, Amp^R^ plasmid expressing oRbs rRNA)^17^.

The expression of o-*gfp* or o-*luc* reporters was measured as described above.

### Analyzing the mutant rRNA content

The presence of the engineered mutations in the 23S rRNA of the orthogonal ribosome was analyzed by primer extension. For that, total RNA was isolated from the OSYRIS cells using the RNeasy Mini Kit (Qiagen). The primers and combination of dNTPs and ddNTPs for analysis of each mutation are shown in Supplementary Table 5. For each assay, the appropriate 5′ [^32^P]-labeled primer (0.5 pmol) was annealed to 1 μg of total RNA in 1× hybridization buffer (50 mM K-HEPES, pH 7.0, 100 mM KCl) by incubating at 90 °C for 1 min and then cooling over 15 min to 42 °C. Annealed primers were extended with 2 units of AMV reverse transcriptase (Roche) in the presence of 0.25 mM of the appropriate ddNTP and 0.2 mM of each of the remaining dNTPs for 20 min at 42 °C (final reaction volume of 8 µl). The reaction was stopped by adding 120 μl of stop buffer (84 mM NaOAc, 0.8 mM EDTA, pH 8.0, 70% EtOH), cooling at −80 °C for 15 min and pelleting nucleic acids by centrifugation 1 h at 15,000*g* (4 °C). The supernatant was removed, the pellet was dried and dissolved in formamide loading dye. The cDNA products were resolved in a 12% denaturing polyacrylamide gel and visualized by phosphorimaging. The intensity of the toeprint bands was determined using the ImageJ software^41^. The background was subtracted.

### Expressing GFP-TnaC in the cell-free translation system

The DNA templates containing the T7 RNA polymerase promoter, ribosome binding site from bacteriophage T7 gene 10 and GFP-TnaC or GFP-TnaC (W12R) coding sequences (see the complete sequence in the Source data file) were generated by cross-over PCR. First, the T7 promoter and the *gfp*-coding sequence were PCR amplified from the pY71-T7-GFP plasmid^42^ using the T7 promoter forward primer NA29 (Supplementary Table 4) and either NA30 complementary to the wt *tnaC* or NA31 complementary to the W12R mutant of the *tnaC* gene. Independently, 3′ segments of the wt or mutant *tnaC* genes with the 3’ untranslated regions were PCR amplified from the plasmids pGF2500-tnaC-wt or pGF2500-tnaC-mut^43^ using forward primers NA32 for wt, or NA33 for the W12R mutant, and a common reverse primer NA34.

Two PCR products corresponding to either the wt or mutant *gfp-tnaC* constructs were then combined together at 400 pg/μl (final concentration) and reamplified using the T7 and TnaC(rev) primers.

In vitro translation of the *gfp-tnaC* templates was carried out in the PURExpress, ΔRibosome, ΔtRNAs, Δamino acids cell-free translation system composed of purified components (New England Biolabs), as described in ref. ^29^ with minor modifications. Reactions were supplemented with a 19-amino acids mixture (final concentration: 0.3 mM of each amino acid) and L-tryptophan to a final concentration of 50 μM (for reactions with low-tryptophan conditions) or 5 mM Trp (for high-tryptophan conditions). PCR-generated DNA templates were added to a final concentration of 5 ng/µl. The reactions were carried out at 37 °C for 3 h in a total volume of 5 μl in 384-well plates with black walls and clear bottom (Falcon) in a plate reader (TECAN Infinite M200 Pro). GFP fluorescence (excitation at 485 nm, emission at 520 nm, optimal gain 30% RFU with applying the gain regulation function) was monitored over time.

### Preparing the PTC mutant library

The PTC mutant library was generated by transferring individual mutations from the pT7rrnB library^44^ into the 23S rRNA gene in the poRbs plasmid.

To prepare the plasmid backbone, the poRbs plasmid was digested with SgsI and Bst1107I restriction enzymes, resulting in the excision of a 1546 nt fragment from the 23S rRNA gene. The reaction products were separated by agarose gel electrophoresis, and the 7483 bp backbone fragment was purified from the gel using Zymoclean Gel DNA Recovery Kit (Zymo Research) and DNA Clean & Concentrator Kit (Zymo Research) sequentially.

To generate the 1606-bp inserts carrying the PTC mutations, individual plasmids of the pT7*rrnB* plasmid library were used as a template for the PCR reaction catalyzed by the Q5 High-Fidelity DNA Polymerase (New England Biolabs) and employing the primers NA35 and NA36. PCR products were cleaned up using the DNA Clean & Concentrator Kit (Zymo Research).

The plasmid backbone (35 ng) and the DNA inserts (60 ng) were mixed in a total volume of 5 μl of a Gibson assembly reaction and incubated for 1 h at 50 °C.

Individual Gibson-assembly reactions were used to transform chemically competent POP2136 cells. The high-throughput transformation was carried out in a flat-bottom tissue culture 96-well plates with low evaporation transparent lid (Falcon). In each well of the plate, 20 µl of competent cells were mixed with 2 μl of individual Gibson assembly reactions. Plates were incubated on ice for 30 min, at 42 °C for 50 s and again on ice for 15 min. One hundred microlitre of SOC medium were added to each well, and cells were allowed to recover at 30 °C for 2 h on a shaker. Culture volumes were reduced to 40 μl by spinning the plate at 6000*g* for 6 min in a swinging bucket rotor and removing 80 μl of supernatant. A 6 µl aliquot of each of the remaining cell suspension were then spot-plated using a multichannel pipettor on LB/agar rectangular OmniTray Single-Well plates (Nunc) supplemented with 50 μg/ml Kan. Plates were incubated at 30 °C for 20 h.

Individual colonies were inoculated in fresh LB media supplemented with 50 μg/ml Kan and grown for 12 h at 30 °C. Plasmids were isolated, and the presence of the desired mutations, as well as the lack of off-target mutations in the PCR-amplified 23S rRNA segments, were confirmed by capillary sequencing.

The individual PTC mutant library plasmids were then introduced into OSYRIS cells by transforming them into SQ171 FG/pRibo-Tt/poGFP-TnaC cells using the high-throughput transformation approach described above with the following modifications: (i) 20 ng of the purified individual plasmids were used in transformation; (ii) transformants were recovered in SOC medium for 6 h at 37 °C and patched onto LB/agar plates supplemented with 50 μg/ml Amp, 25 μg/ml Kan, 25 μg/ml Spc, and 15 μg/ml Chl; (iii) plates were incubated at 37 °C for 48 h; (iv) glycerol stocks were prepared in 96-well plates from cultures grown from individual colonies of the transformants.

### PTC library screening

Individual colonies of the OSYRIS cells carrying the PTC library mutants were inoculated in the wells of a 96-well plate containing 120 µl of LB media, supplemented with 50 μg/ml Amp, 25 μg/ml Kan, 25 μg/ml Spc, and 15 μg/ml Chl, and grown for 24 h at 37 °C with constant shaking. Cultures were diluted 1:40 (v/v) in 120 µl of fresh LB supplemented with the same antibiotics, 0.35 mg/ml 1-methyl-tryptophan (Sigma) and 0.016 ng/ml of HSL. Plates were placed into TECAN Infinite M200 Pro plate reader and incubated at 37 °C with constant linear (3 mm) shaking. Optical density (*A*_600_) of the cultures and oGFP fluorescence were monitored as described above.

The termination arrest bypass score was calculated by comparing the efficiency of GFP expression in the OSYRIS cells carrying GFP-TnaC(W12R) mutant construct to that in the OSYRIS cells carrying wt GFP-TnaC construct. The SB score values were computed based on the readings obtained at the 48 h time point using the following formula:

1 $${\mathrm{SB}}\,{\mathrm{score}} = \frac{{{\mathrm{RFU}}\left( {{\mathrm{WT}}} \right){/A}_{600}\left( {{\mathrm{WT}}} \right)}}{{{\mathrm{RFU}}\left( {{\mathrm{W}}12{\mathrm{R}}} \right){/A}_{600}\left( {{\mathrm{W}}12{\mathrm{R}}} \right)}},$$

where RFU is relative fluorescence units.

The mean SB score values were calculated using data obtained in two independent experiments.

### Isolating the 50S subunits from the OSYRIS cells

The ribosomes were isolated from the OSYRIS cells following the protocol described by Ohashi et al.^45^. Specifically, OSYRIS cells expressing ribosomes with mutations U2500G, A2060C, or A2450U in 23S rRNA were grown overnight at 37 °C in LB medium supplemented with 50 μg/ml Amp, 25 μg/ml Kan, 25 μg/ml Spc, and 15 μg/ml Chl. The cultures were diluted to the final *A*_600_ = 0.003 into 1 L of fresh LB media supplemented with the same antibiotics and grown for approximately 15 h with vigorous shaking until optical density reached *A*_600_ = 0.35. Cells were collected by centrifugation for 15 min at 5000*g* (4 °C), and cell pellets were flash-frozen in liquid nitrogen and stored at −80 °C. Frozen cell pellets were resuspended in 20 ml of lysis buffer (10 mM HEPES-KOH, pH 7.6, 50 mM KCl, 10 mM Mg(OAc)_2_, 7 mM β-mercaptoethanol), lysed in EmulsiFlex-C3 homogenizer (AVESTIN Inc.) at 15,000 psi for 5 min and then lysates were clarified by 30 min centrifugation at 20,000*g* (4 °C) and transferred to new centrifuge tubes. Ammonium sulfate was added to the final concentration of 1.5 M and tubes were centrifuged for 1 h at 20,000 g (4 °C). The ribosome (Ribo-T + dissociable ribosomes)-containing supernatant was filtered through a 0.22-µm ∅ 30 mm polyethersulfone (PES) membrane filter (CELLTREAT Scientific Products). Ribosome material was purified by hydrophobic chromatography using a 5 ml HiTrap Butyl FF column (GE Healthcare Life Sciences), equilibrated with 20 mM HEPES-KOH, pH 7.6, 10 mM Mg(OAc)_2_, 7 mM β-mercaptoethanol, 1.5 M (NH_4_)_2_SO_4_, on an AKTApurifier UPC 10 (GE Healthcare). After loading the material, the column was washed with 20 mM HEPES-KOH, pH 7.6, 10 mM Mg(OAc)_2_, 7 mM β-mercaptoethanol, 1.2 M (NH_4_)_2_SO_4_, and the ribosomes were then eluted with the buffer containing 20 mM HEPES-KOH, pH 7.6, 10 mM Mg(OAc)_2_, 7 mM β-mercaptoethanol, 0.75 M (NH_4_)_2_SO_4_. Eluate fractions containing ribosomes were pulled together and loaded onto 16 ml 30% sucrose cushion prepared in the buffer 20 mM HEPES-KOH, pH 7.6, 10 mM Mg(OAc)_2_, 30 mM NH_4_Cl, 7 mM β-mercaptoethanol in 35 ml centrifuge tubes. Ribosomes were pelleted by centrifugation at 10,000*g* for 18 h at 4 °C in the Type 70 Ti rotor (Beckman). Ribosome pellets were resuspended in the dissociation/storage buffer (20 mM HEPES-KOH pH 7.6, 30 mM KCl, 1.5 mM Mg(OAc)_2_, 7 mM β-mercaptoethanol) and aliquots were flash-frozen and stored at −80 °C.

To isolate individual 50 S ribosomal subunits, the ribosome preparations were loaded on 10–40% sucrose gradients prepared in buffer 20 mM Tris-HCl, pH 7.5, 1.5 mM Mg(OAc)_2_, 100 mM NH_4_Cl, 2 mM β-mercaptoethanol in the centrifuge tubes for the SW41 rotor (Beckman). The gradients were centrifuged for 16 h at 83,000*g* at 4 °C and fractionated on a gradient fractionator (BioComp) with *A*_254_ monitoring. Fractions corresponding to the large ribosomal subunits were pooled, concentrated on Vivaspin 2 ml concentrators with cellulose triacetate membrane (Sartorius Stedim Biotech GmbH) and recovered in the ribosome storage buffer (20 mM HEPES-KOH pH 7.6, 30 mM KCl, 6 mM Mg(OAc)_2_, 7 mM β-mercaptoethanol). The aliquots were flash-frozen and stored at −80 °C.

### Isolating ribosomes with non-lethal 23S rRNA mutations

Ribosomes carrying non-lethal mutations in the 23S rRNA (A2503G, A2062G, C2611G, and C2611U) were isolated from the SQ171 cells carrying pAM552 plasmids with the corresponding mutations and expressing pure populations of the mutant ribosomes^14,46^. The ribosomes were isolated as described above except that after sucrose cushion centrifugation, the ribosomal pellets were resuspended in the ribosome storage buffer (20 mM HEPES-KOH pH 7.6, 30 mM KCl, 6 mM Mg(OAc)_2_, 7 mM β-mercaptoethanol). The aliquots were flash-frozen and stored at −80 °C.

### Toe-printing analysis

Primer extension inhibition (toeprinting) analysis^47^ was performed following a published protocol^48^. When needed, the prolyl-tRNA synthetase inhibitor 5′-O-[N-(L-prolyl)-sulfamoyl] adenosine (L-PSA)^49^ was added to the reactions to the final concentrations of 50 µM. After separation of the primer extension products in the sequencing gel and phosphorimaging, the intensity of the toeprint bands was determined using the ImageJ software^41^. The efficiency of the TnaC-induced translation arrest at the *tnaC* stop codon was calculated by comparing the intensity of the stop codon (SC) toeprint band (green arrowhead in Supplementary Fig. 11c) with the intensity of the toeprint band at the preceding codon (PC) in the L-PSA-containing samples (open arrowhead in Supplementary Fig. 11c) using the formula:

2 $${\mathrm{TnaC}} - {\mathrm{induced}}\,{\mathrm{translation}}\,{\mathrm{arrest}} = \frac{{{\mathrm{SC}} - {\mathrm{SC}}_{{\mathrm{BG}}}}}{{{\mathrm{PC}} - {\mathrm{PC}}_{{\mathrm{BG}}}}} \ast 100,$$

where SC_BG_ and PC_BG_ are backgrounds for the corresponding bands.

### Structural analysis and figure preparation

For calculating the distances of the 23S rRNA nucleotides to the attacking α-amino group of the A-site amino acid, the crystal structure of the *Thermus thermophilus* ribosomes with P- and A-site tRNAs in the pre-attack state (PDB 1VY4)^23^ were aligned on the basis of the full-length 23S rRNA with the high-resolution structure of the partially rotated vacant *E. coli* ribosome (PDB 4YBB)^50^. The distance measurements and figure rendering were performed in PyMOL (Molecular Graphics System, Version 2.0 Schrödinger, LLC). Figure 4g was prepared by aligning the cryo-EM structure of the *E. coli* ribosomes stalled with the TnaC-tRNA in the P site (PDB 4UY8)^30^ with the crystallographic structure of *T. thermophilus* ribosome complexed with RF2 (PDB 4V67)^51^.

### Statistical analysis

Where relevant, statistical values can be found in the figure legends. The mean of the value was defined as the arithmetic mean. Depending on the numbers of the independent biological replicates (*n*), deviation ranges represent either standard deviation (s.d.) (*n* ≥ 3) or experimental error (*n* = 2). All statistical values were calculated and all graphs were plotted using the Microsoft Excel 365 software. The Student’s *t* test was performed using GraphPad Prism version 8.00 for Windows (GraphPad Software, La Jolla, CA, USA).

### Reporting summary

Further information on research design is available in the Nature Research Reporting Summary linked to this article.

## Supplementary information

Supplementary Information

Reporting Summary

Supplementary Data 1

Description of Additional Supplementary Files

Peer Review File

## Data Availability

The data that support the findings of this study are available from the corresponding author upon reasonable request. The source data underlying Figs. 3a, 4b, 5a, 6b, Supplementary Figs. 4a,b, 5a–c, 6, 8, and 7c are provided in the Source Data file.

## Source data

Source Data
