# Supplementary material for: A fully orthogonal system for protein synthesis in bacterial cells
Source: Nat Commun. 2020 Apr 20;11:1858. doi: 10.1038/s41467-020-15756-1 (PMC7170887; doi:10.1038/s41467-020-15756-1)
Supplement: Supplementary file 1 — Supplementary Information [file 41467_2020_15756_MOESM1_ESM.pdf]

SUPPLEMENTARY INFORMATION

A fully orthogonal system for protein synthesis in bacterial cells

Nikolay A. Aleksashin, Teresa Szal, Anne E. d'Aquino, Michael C. Jewett, Nora Vázquez-Laslop, and Alexander S. Mankin

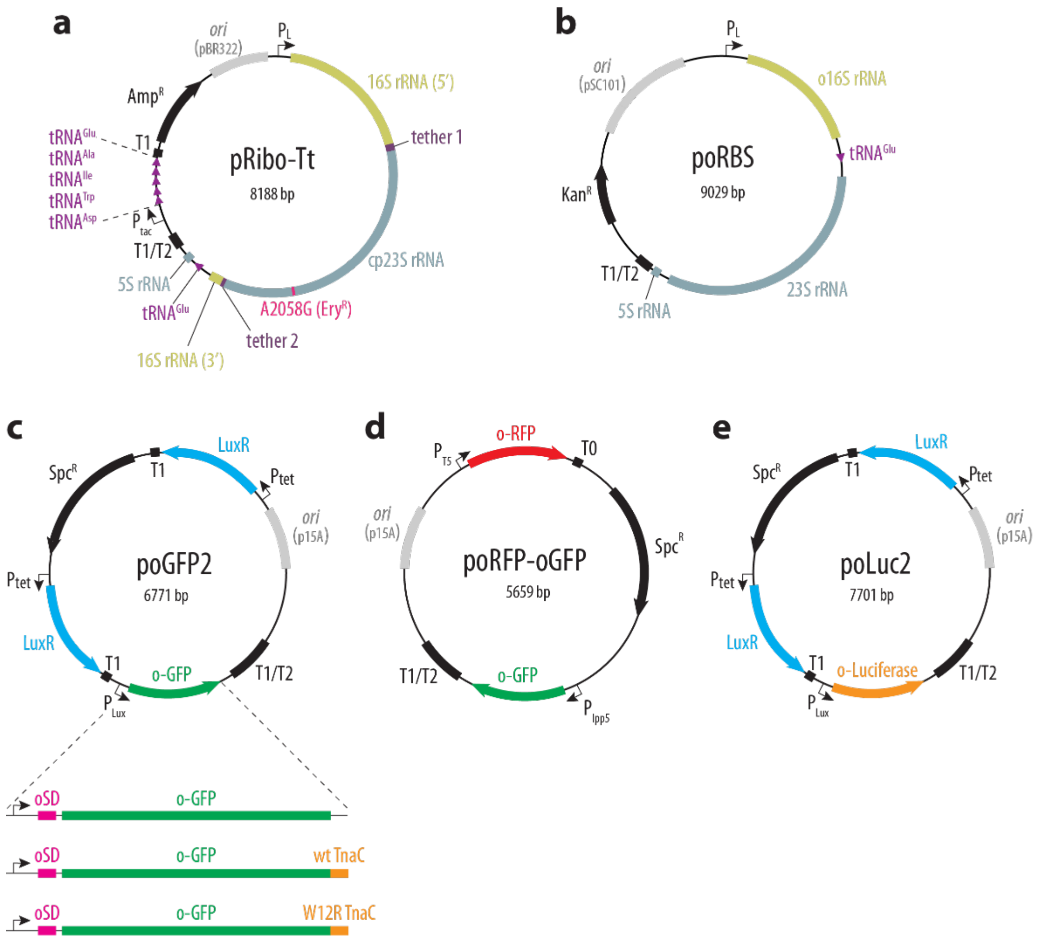

Supplementary Figure 1. The main plasmids of the OSYRIS. a The map of the pRibo-Tt plasmid. The genes encoding the 16S-23S rRNA hybrid and 5S rRNA are controlled by the lambda P_L promoter. In the 16S-23S rRNA hybrid, the circularly permutated 23S rRNA opened at the loop of helix 101, is inserted into the loop of helix 44 of the 16S rRNA by way of two RNA tethers [1,2]. The 23S rRNA segment carries the A2058G mutation rendering Ribo-T erythromycin-resistant. The cluster of the tRNA genes, which are missing in the host cells due to the deletion of chromosomal rRNA operons[3] is under control of the P_{tac} promoter. **b** The poRBS plasmid, derived from pAM552[1], carries the *E. coli rrnB* operon with the altered ASD sequence GUGGUU in the 16S rRNA gene[2]. The control plasmid pRbs (not shown) is identical to poRbs except that it contains wt ASD in the 16S rRNA genes. **c-e** The reporter plasmids carry either the gene of the superfolder green fluorescent protein (*gfp*) (poGFP2) **(c)**, the combination of *gfp* and the red fluorescent protein gene (poRFP-oGFP) **(d),** or the gene of the firefly luciferase (poLuc2) **(e)**. The coding sequences of the reporters are preceded by the altered (orthogonal) SD (oSD) sequence, AACCAC[2] cognate to the ASD in the o16S rRNA encoded in poRbs. Transcription of the reporters in poGFP2 and poLuc2 plasmids is controlled by the inducible P_{Lux} promoter regulated by binding of N-(β-ketocaproyl)-L-homoserine lactone (HSL) to the LuxR repressor. Two copies of the luxR gene are present in the plasmids poGFP2 and poLuc2 due to a spontaneous event. **d** The *gfp* and *rfp* genes in poGFP-poRFP are under control of the P_{lpp5} and P_{T5} promoters, respectively. The fully annotated sequences of the plasmids shown in this figure can be found in the Source data file.

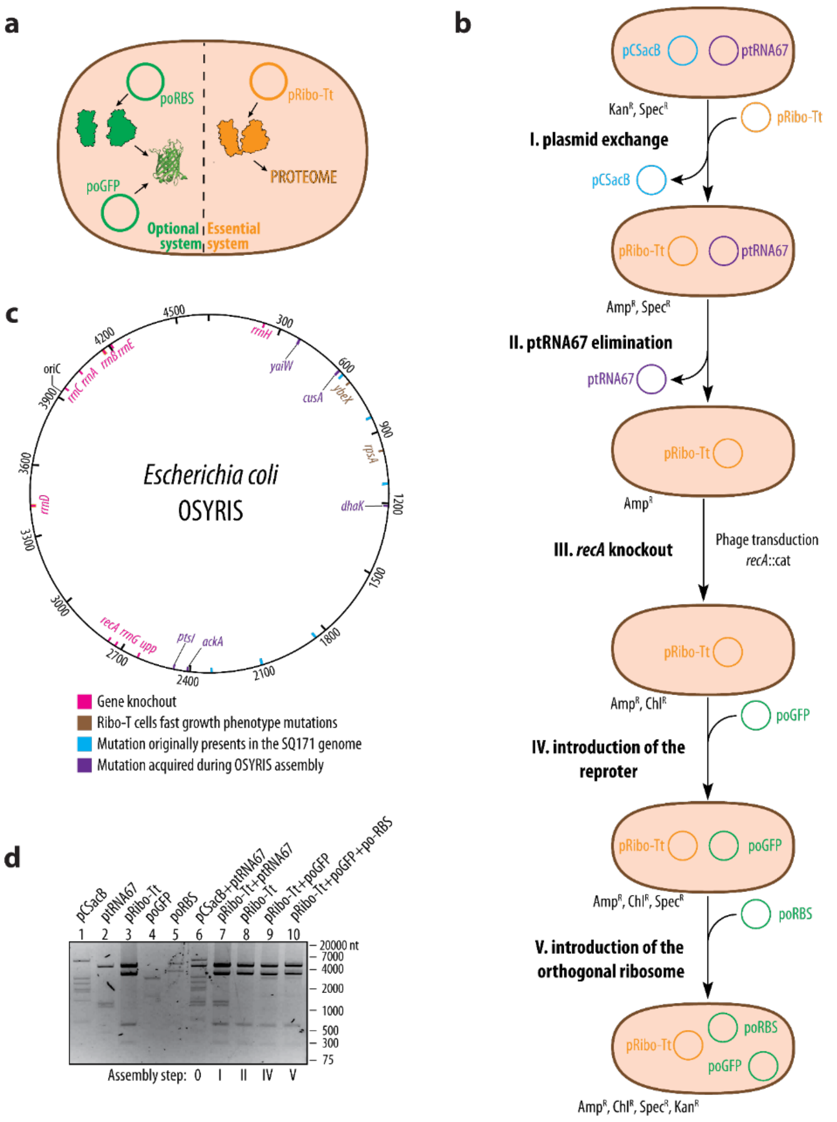

Supplementary Figure 2. The OSYRIS assembly in *E. coli* cells. a The plasmid composition of the OSYRIS cells. Ribo-T, that translates the cellular proteome, is expressed from the pRibo-Tt plasmid. The mRNA, transcribed from the orthogonal reporter gene on the poGFP (or poRFP/oGFP) plasmid, is translated by the o-ribosome whose rRNA is encoded in the poRbs plasmid. **b** Sequential steps for the construction of the OSYRIS cells. The genome of the cells was completely sequenced after the assembly step III (see panel **c**). In the next two steps, cells were subsequently transformed with the reporter plasmids (poGFP in the illustrated example) and then with poRbs (or by the plasmids of the PTC mutant library described in Fig. 7). Antibiotics resistance of cells generated at every step is indicated. **c** The genome of the OSYRIS cells. The starting SQ171 FG strain was derived from *Escherichia coli* MG1655 cells[4]. Five spontaneous mutations (purple) were acquired during OSYRIS cells assembly and propagation; the precise positions of the mutations and functions of the affected genes are listed in Supplementary Table 4. Numbers outside of the circle indicate genome nucleotide numbering. **d** Gel electrophoresis analysis of the plasmid content of the cells from the different steps of OSYRIS assembly (shown in panel **b**). Plasmid preparations were digested with a mixture of KpnI, BamHI and HindIII restriction enzymes. Restriction digest of the individual plasmids is shown for reference. The uncropped gel can be found in the Source data file.

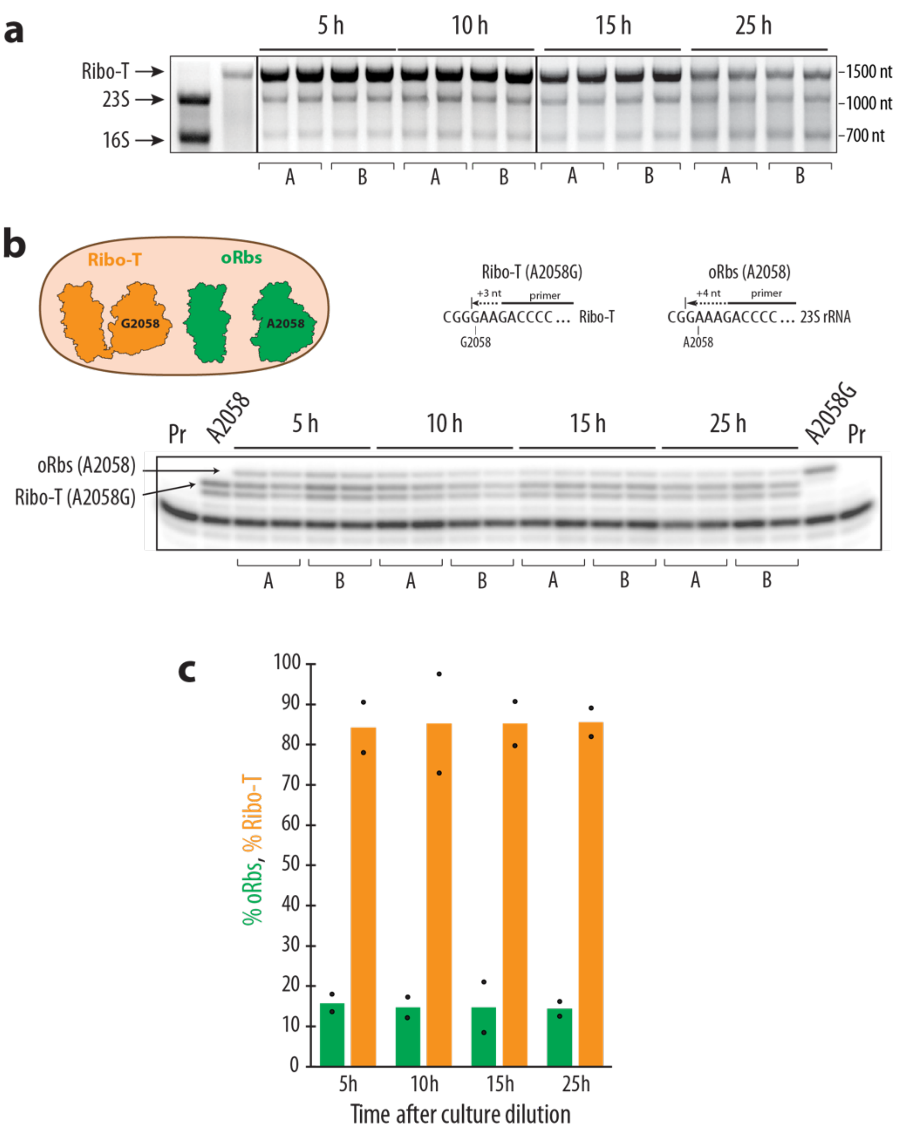

Supplementary Figure 3. oRbs are stably expressed in OSYRIS cells. a Agarose gel-electrophoresis analysis of total RNA maintained in OSYRIS cells after dilution from an overnight culture. Two independent colonies (A and B) of OSYRIS cells with poGFP plasmid were grown overnight and diluted each 1:50 into two tubes with LB medium supplemented with 50 μg/ml Amp, 25 μg/ml Kan and 15 μg/ml Spc. Total RNA was isolated after the indicated time intervals. Two technical replicates for each culture were processed independently and run in separate lanes of the gel. **b** Primer extension analysis of the representation of oRbs (which has wt A2058) relative to Ribo-T (which carries the A2058G mutation) in the OSYRIS cells over time. The principle of primer extension analysis is illustrated above the sequencing gel. Total RNA prepared from OSYRIS cells (see panel **a**) was used as a template for primer extension. RNA samples prepared from wt *E. coli* cells ('A2058') and from cells expressing only Ribo-T ('A2058G') were used as controls. Lanes marked 'Pr' contain the [^{32}P]-labeled DNA primer. The uncropped gels shown in panels **a** and **b** can be found in the Source data file. **c** Quantitation of the relative intensity of the Ribo-T and oRbs-specific bands in the gel shown in panel **(b)** was used to assess the relative representation of two ribosome species in OSYRIS cells. The bar graph represents the mean of two independent biological replicates (individual experimental data points are shown by black dots).

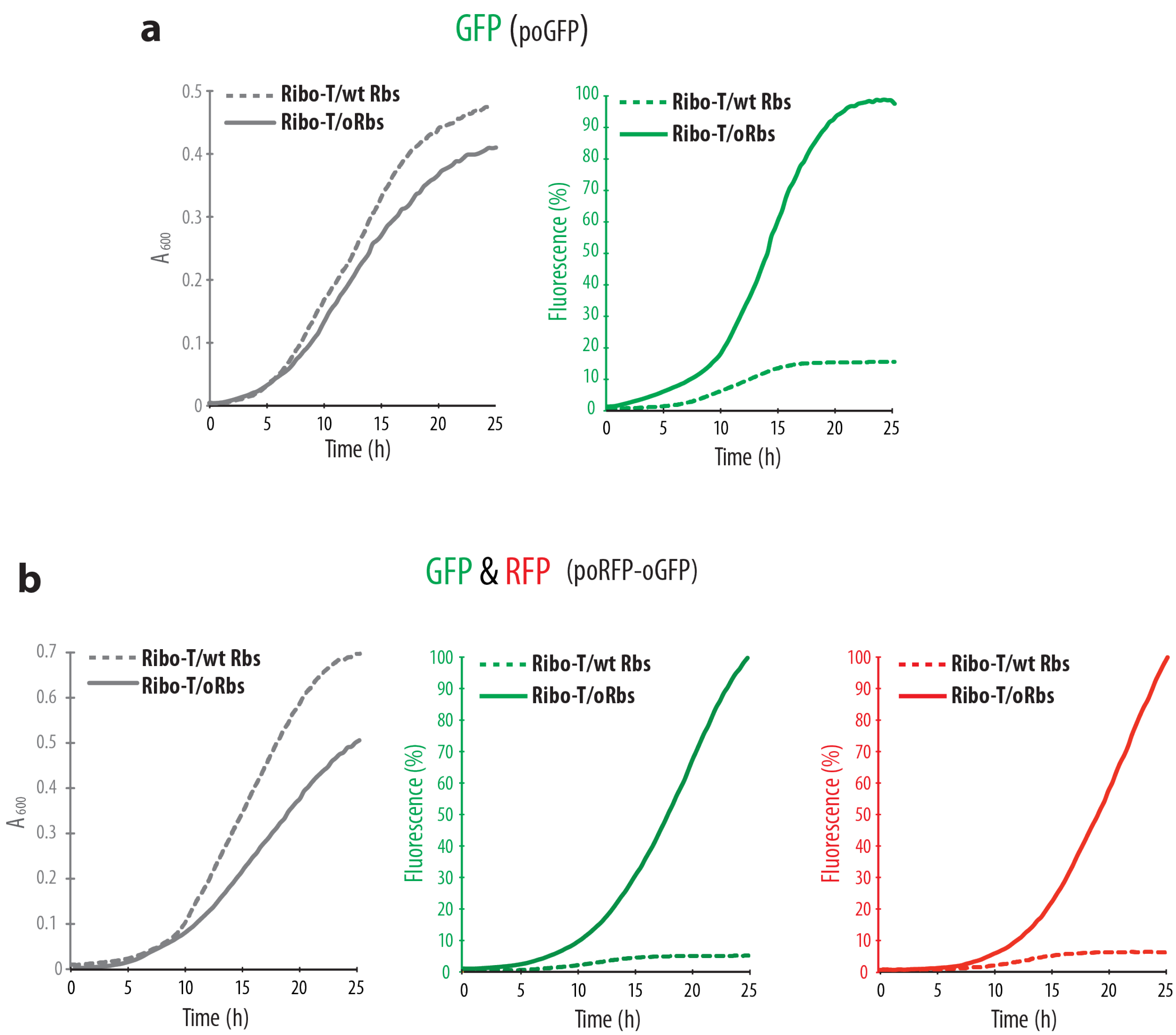

Supplementary Figure 4. Efficient translation of the orthogonal reporters in OSYRIS cells. a Growth curves (top) of the OSYRIS cells containing either o-ribosomes (oRbs) (solid lines) or wt ribosomes (wt Rbs) (dashed lines) and expression of the orthogonal GFP reporter therein (bottom). **b,** Growth curves (top) and expression of the orthogonal GFP (green) and RFP (red) reporters (bottom) in OSYRIS cells expressing oRbs (solid lines) or wt Rbs (dashed lines). The highest fluorescence reading (relative fluorescence units) in each experiment was taken as 100%.

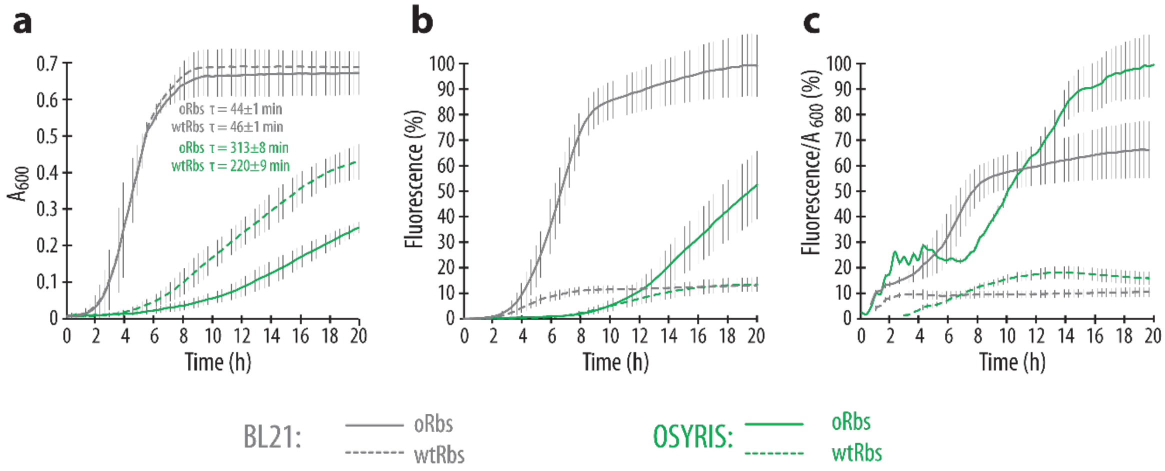

Supplementary Figure 5. Expression of the orthogonal *gfp* reporter in OSYRIS cells and in *E. coli* strain BL21. a Growth curves, **b** GFP fluorescence, and **c** normalized GFP fluorescence (oGFP fluorescence / A_{600}) in OSYRIS cells (green curves) and BL21 cells (grey curves) grown in 96-well plates. Both types of cells express either wt ribosomes (wt Rbs) (dashed lines) or o-ribosomes (oRbs) (solid lines). Notice that the normalized oGFP fluorescence (or oGFP fluorescence per cell) is higher in the OSYRIS cells than in the BL21 cells (**c**). The data represent the mean of three independent biological replicates; error bars indicate s.d. In (**b**), the highest fluorescence reading (relative fluorescence units) for BL21 cells was set as 100%. In (**c**), the highest normalized fluorescence reading for OSYRIS cells was taken as 100%. The raw data can be found in the Source data file.

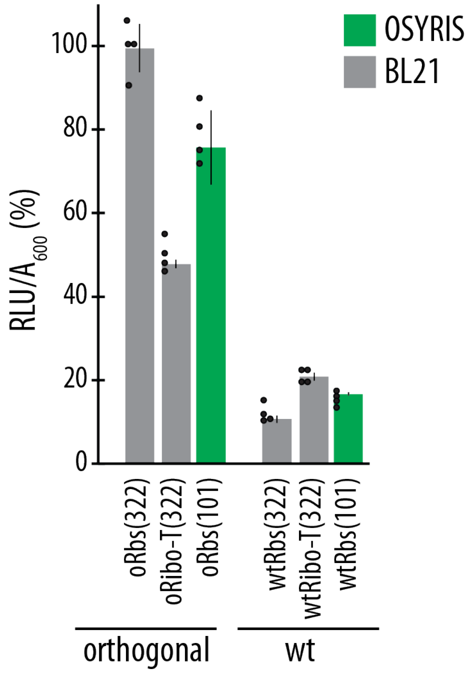

Supplementary Figure 6. oRbs outperforms oRibo-T in expressiong the orthogonal luciferase reporter. Expression of o-*luc* in BL21 or in OSYRIS cells driven by dissociable oRbs or oRibo-T. BL21 cells with the reporter plasmids poLuc2 were transformed with the medium copy number (pBR322 *ori*) plasmids o-pAM552 or with poRibo-T expressing oRbs or oRibo-T, respectively. OSYRIS cells express oRbs from a low copy number plasmid poRbs. Control cells were transformed with the same plasmids but carrying rRNA with wt ASD (see Supplementary Fig. 1 for information about the plasmids). The relative reporter expression was recorded as described in the Methods section. The bar graph represents the mean ± s.d. of four biological replicates. The raw data can be found in the Source data file.

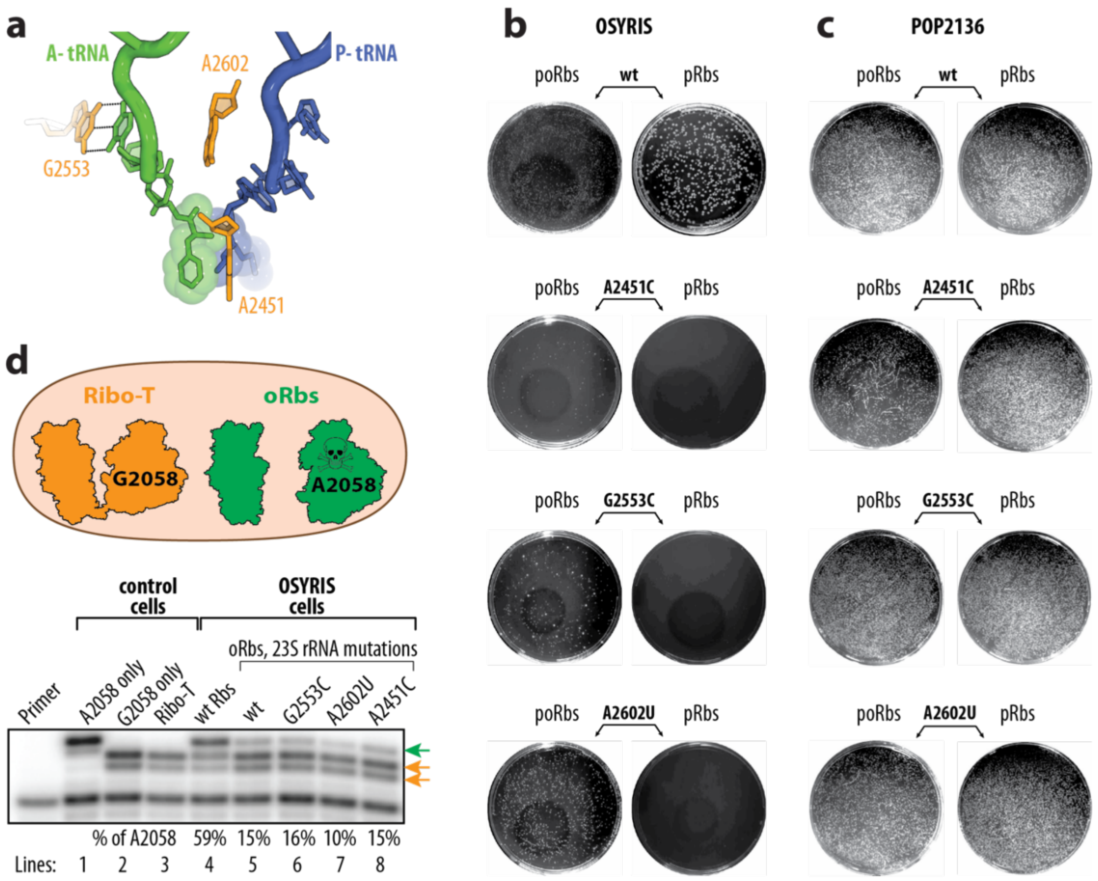

Supplementary Figure 7. The viability of the OSYRIS cells expressing lethal mutations in the rRNA of the 50S subunit of the orthogonal ribosome demonstrates functional isolation of the two orthogonal translation systems. a Locations of 23S rRNA nucleotides G2553, A2602, A2451 (orange) in the PTC active site (PDB 1VY4)[5]. Mutations of these nucleotides are dominantly lethal in wt *E. coli* cells[6]. A-site tRNA is green and P-site tRNA is blue. **b** Transformation of the OSYRIS cells yields viable colonies when mutant 23S rRNA carrying lethal mutations is co-expressed with the orthogonal 16S rRNA (poRbs), but not when it is co-expressed with wt 16S rRNA (pRbs). **c** Transformation of the POP2136 strain where the expression of the rRNAs operon from the pRbs and poRbs plasmids is repressed[7], yields many colonies. **d** Top: The ribosome composition in the OSYRIS cells expressing Ribo-T with the A2058G mutation and dissociable orthogonal ribosome (oRbs) (green) whose 50S subunit, with wt A2058, carries a single rRNA nucleotide lethal mutation (represented by a skull). Bottom: Primer extension analysis of the rRNA region proximal to nucleotide 2058 showing stable maintenance in OSYRIS cells of the 50S subunits with lethal mutations (and wt A2058) alongside with Ribo-T carrying the A2058G mutation. Lanes 1-3: control primer extensions on preparations of the wt 23S rRNA (lane 1), 23S rRNA with the A2058G mutation (lane 2), or RNA extracted from the OSYRIS cells expressing only Ribo-T (lane 3). Lane 4, rRNA from the OSYRIS cells transformed with pRbs and expressing wt Rbs. Lanes 5-8, rRNA from the cells expressing oRbs with no mutations in the 23S rRNA (lane 4) (none) or with the indicated lethal mutations in the 23S rRNA (lanes 5-8). Numbers under the lanes of the gel indicate the content (%) of the 23S rRNA estimated as the ratio of the intensity of the cDNA band representing 23S rRNA (orange arrows) to the sum of intensities of the 23S rRNA- and Ribo-T-specific bands (green and orange arrows, respectively). Shown is a representative gel of three independent biological replicates. Uncropped gel can be found in the Source data file.

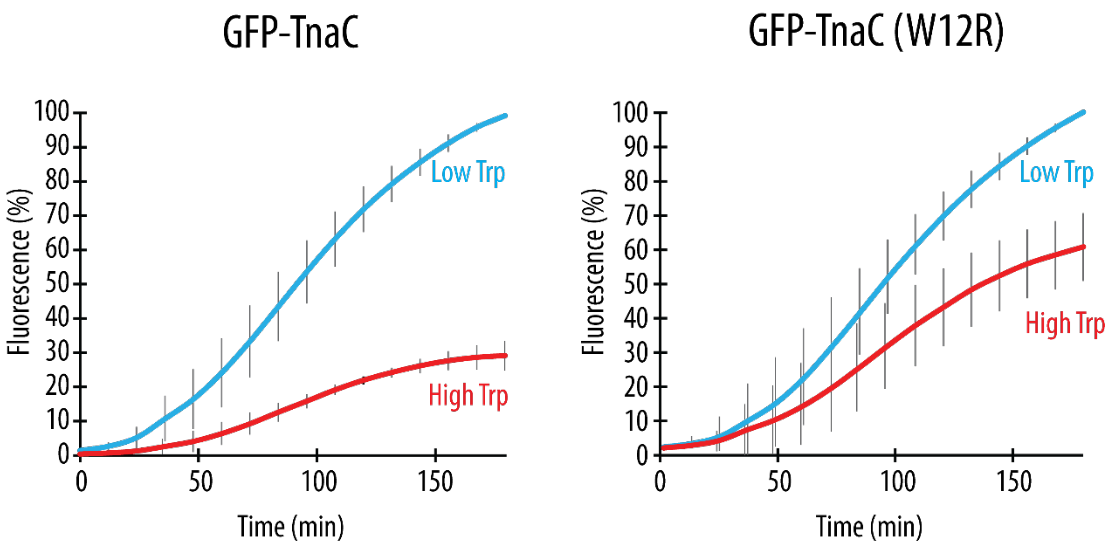

Supplementary Figure 8. TnaC-mediated inhibition of in vitro translation of the reporter protein. Translation of the GFP-TnaC or GFP-TnaC(W12R) reporters was carried out in the PURExpress cell-free system in the presence of low (50 μM) or high (5 mM) L-tryptophan concentration. The TnaC mutation W12R is known to diminish the TnaC-mediated inhibition of the protein release at the stop codon at high L-tryptophan concentration [8]. The data represent the results of the three independent experiments, and the error bars indicate the experimental error (mean +/- s.d.). The raw data and sequences of the DNA templates can be found in the Source data file. The highest fluorescence reading (relative fluorescence units) in each experiment was taken as 100%.

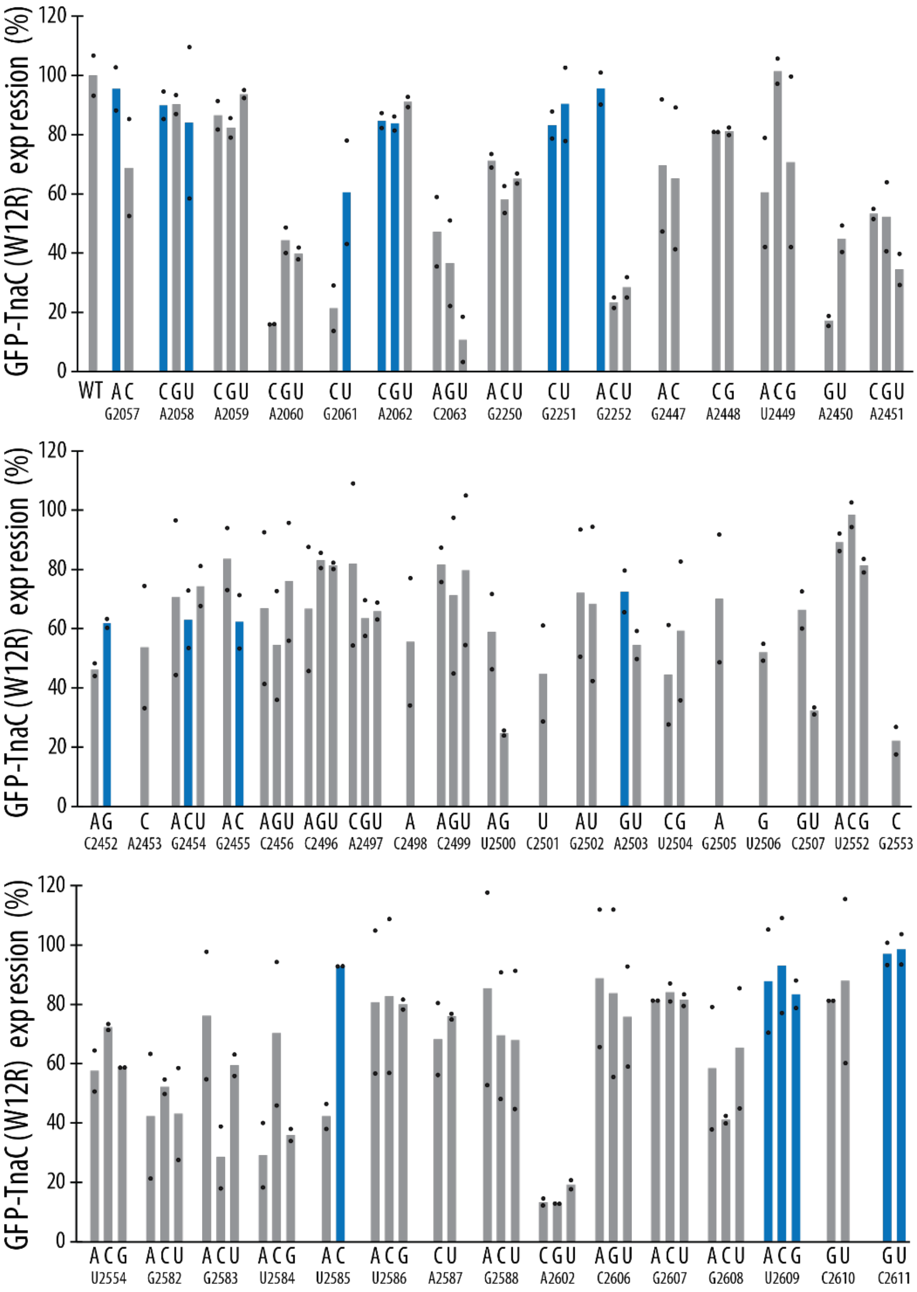

Supplementary Figure 9. The translation activity of the PTC library mutants in OSYRIS cells. Translation activity of the individual mutants was estimated by comparing the expression of the o-GFP-TnaC (W12R) reporter (which shows partial stalling relieve) (see Figure 6 and Supplementary Fig. 8) in the OSYRIS cells with the mutant oRbs to the expression of the same reporter in OSYRIS cells containing oRbs with wt 23S rRNA (100%). The respective wt 23S rRNA residues are indicated, and the identity of the assessed mutants is shown. High translation activity was defined as that where reporter expression was >60%. The gain-of-function mutants that combine high SB score with high translation activity are shown in blue (see Fig. 8a and Supplementary Fig. 10). The bars represent the mean of two independent biological replicates; black dots indicate the experimental data points. The numeric data can be found in Supplementary Table 1. The normalized fluorescence reading (relative fluorescence units over A_{600}) of OSYRIS cells containing oRbs with wt 23S rRNA was taken as 100%.

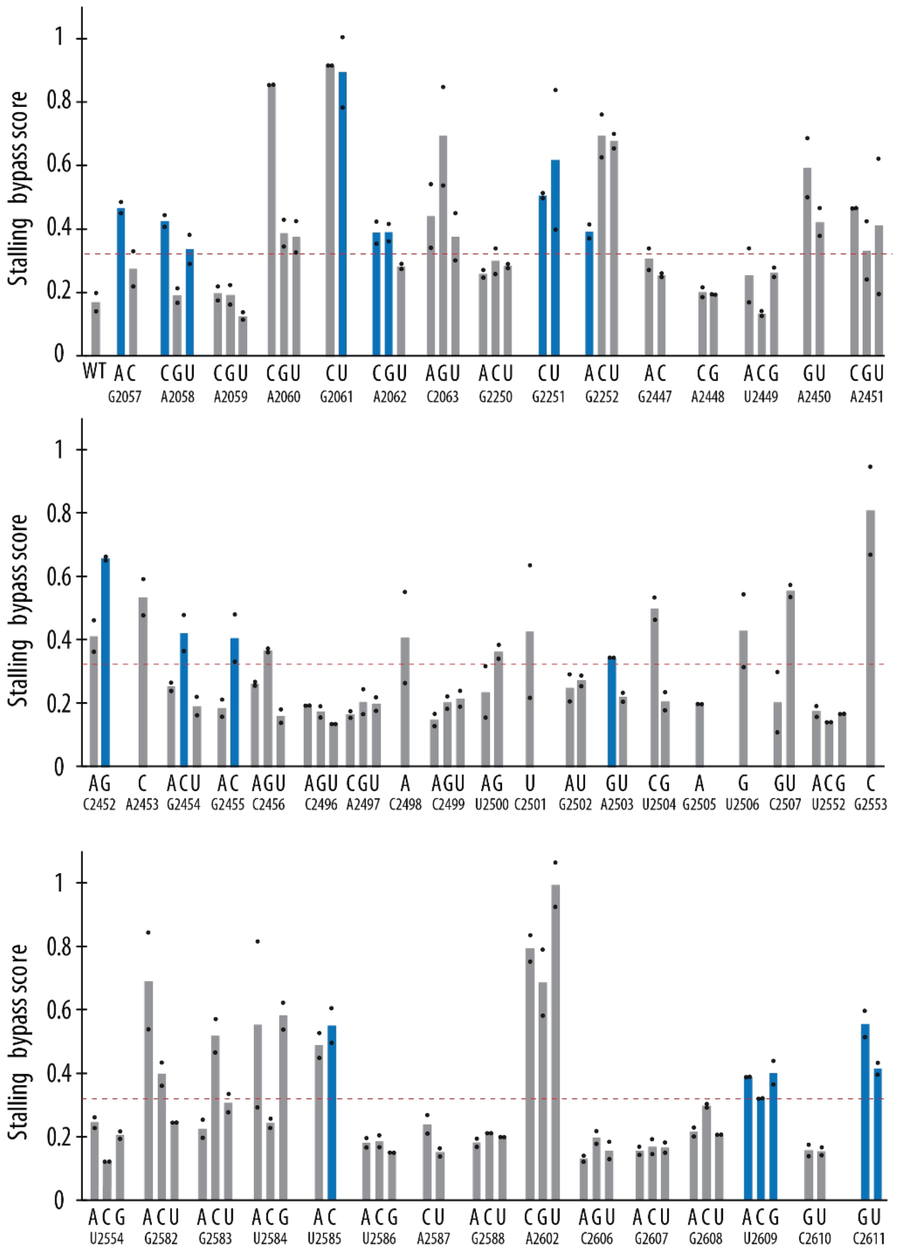

Supplementary Figure 10. The termination stalling bypass scores of the individual PTC mutants. TnaC stalling bypass score (SB) was calculated as the ratio of GFP fluorescence (normalized by cell density) in OSYRIS cells expressing GFP-TnaC relative to that in cells with the GFP-TnaC(W12R) reporter. The SB score for cells with oRbs with wt 23S rRNA is 0.17. A threshold high SB score (≥0.3, indicated as a dashed red line) was defined as that afforded by the U2609C mutation, which has been reported to diminish the translation arrest at the *tnaC* stop codon [9,10]. The respective wt 23S rRNA residues are indicated and the identity of the assessed mutants is shown. The gain-of-function mutants that combine high bypass score with high translation activity (see Fig. 8a and Supplementary Fig. 9) are shown in blue. The data represents the results of two independent biological replicates; black dots indicate the experimental data points. The numeric data can be found in Supplementary Table 1.

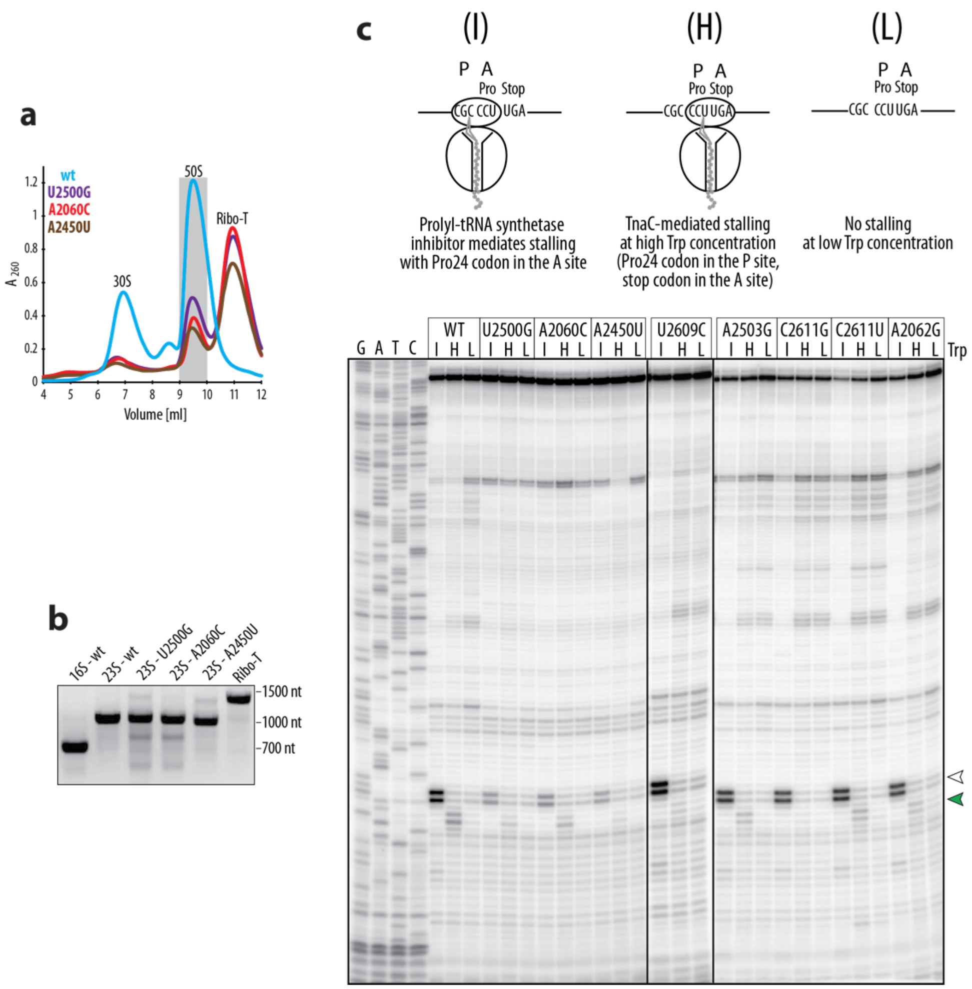

Supplementary Figure 11. Testing the gain-of-function mutants in a cell-free translation system. a Sucrose gradient fractionation under subunit-dissociation conditions of the ribosomal material from OSYRIS cells. The 30S and 50S subunits prepared from dissociated wt ribosomes (blue trace) were used as markers. Gray shading indicates the 50S subunit fractions that were collected and used in the cell-free translation experiments. The Ribo-T peak is indicated. **b** Analysis of the purity of the 50S material (isolated as described in **a**) by agarose gel electrophoresis of the rRNA. Wt 16S and 23S, as well as purified Ribo-T rRNAs, were used as mobility markers. Uncropped gel can be found in the Source data file. **c** The principle (top) and results (bottom) of the *in vitro* toeprinting experiments. The *tnaC* template was translated by ribosomes assembled from the isolated mutant 50S subunits and wt 30S subunits. For each mutant, translation reactions were performed under three different conditions: (I) in the presence of the inhibitor L-PSA of prolyl-tRNA synthetase[11] that stalls the ribosome with the *tnaC* Pro24 codon in the A site; the intensity of the corresponding toeprinting band (indicated by an open arrowhead) reflects the translation activity of the mutant ribosome; (H) in the presence of high concentration of L-tryptophan (5 mM) that leads to translation arrest at the *tnaC* stop codon (indicated by the green arrowhead) unless the rRNA mutation alleviates stalling; (L) at low concentration of L-tryptophan (5 µM), when none or limited stalling at the *tnaC* stop codon is detected. The termination stalling efficiency (Fig. 8b) was estimated by the ratio of the intensity of the stop codon toeprint bands of the H samples relative to those of the Pro24 codon in the I samples.

Supplementary Table 1. Translation activity and SB score of the PTC mutants.

23S rRNA nucleotide number	Mutation	Translation activity (%)[a)]	SB score	Distance from the PTC active site (Å)[b)]
G2057	A<br>C	100 ± 6.7<br>95 ± 7.3	0.47 ± 0.02<br>0.27 ± 0.06	18.5
A2058	C<br>G<br>U	69 ± 16.5<br>90 ± 4.7<br>90 ± 3.3	0.43 ± 0.02<br>0.19 ± 0.02<br>0.34 ± 0.05	16.8
A2059	C<br>G<br>U	84 ± 25.6<br>86 ± 4.8<br>82 ± 3.3	0.20 ± 0.02<br>0.19 ± 0.03<br>0.13 ± 0.01	14.9
A2060	C<br>G<br>U	94 ± 1.4<br>16 ± 0.5<br>44 ± 4.3	0.85 ± 0.01<br>0.39 ± 0.04<br>0.38 ± 0.05	18.5
G2061	C<br>U	40 ± 2.1<br>21 ± 7.7	0.92 ± 0.01<br>0.90 ± 0.11	6.3
A2062	C<br>G<br>U	61 ± 17.5<br>85 ± 2.5<br>84 ± 2.5	0.39 ± 0.03<br>0.39 ± 0.03<br>0.28 ± 0.01	9.3
C2063	A<br>G<br>U	91 ± 1.7<br>47 ± 11.7<br>37 ± 14.6	0.44 ± 0.10<br>0.69 ± 0.16<br>0.38 ± 0.07	5.2
G2250	A<br>C<br>U	11 ± 7.5<br>71 ± 2.3<br>58 ± 4.6	0.26 ± 0.01<br>0.30 ± 0.04<br>0.28 ± 0.01	19.3
G2251	C<br>U	65 ± 1.7<br>83 ± 4.5	0.51 ± 0.01<br>0.62 ± 0.22	11.5
G2252	A<br>C<br>U	90 ± 12.5<br>96 ± 5.4<br>23 ± 1.7	0.39 ± 0.02<br>0.70 ± 0.07<br>0.68 ± 0.02	13.8
G2447	A<br>C	28 ± 3.4<br>70 ± 22.4	0.30 ± 0.03<br>0.25 ± 0.01	10.1
A2448	C<br>G	65 ± 24.2<br>81 ± 0.4	0.20 ± 0.01<br>0.19 ± 0.01	19
U2449	A<br>C<br>G	81 ± 1.3<br>61 ± 18.5<br>101 ± 4.4	0.26 ± 0.09<br>0.13 ± 0.01<br>0.26 ± 0.02	14.4
A2450	G<br>U	71 ± 28.9<br>17 ± 1.9	0.59 ± 0.09<br>0.42 ± 0.05	6.9
A2451	C<br>G<br>U	45 ± 4.5<br>53 ± 1.6<br>52 ± 11.8	0.46 ± 0.01<br>0.33 ± 0.09<br>0.41 ± 0.21	3.8
C2452	A<br>G	35 ± 5.4<br>46 ± 2.1	0.41 ± 0.05<br>0.66 ± 0.01	7.1

A2453	C	62 ± 1.4	0.54 ± 0.06	11
G2454	A	54 ± 20.7	0.25 ± 0.01	15.1
	C	71 ± 26.2	0.42 ± 0.06	
	U	63 ± 9.8	0.19 ± 0.03	
G2455	A	74 ± 6.8	0.19 ± 0.03	17
	C	84 ± 10.5	0.41 ± 0.08	
C2456	A	62 ± 9.1	0.26 ± 0.01	20
	G	67 ± 25.8	0.37 ± 0.01	
	U	54 ± 18.5	0.16 ± 0.02	
C2496	A	76 ± 19.9	0.19 ± 0.01	16.2
	G	67 ± 21.0	0.18 ± 0.01	
	U	83 ± 2.6	0.14 ± 0.01	
A2497	C	81 ± 1.1	0.17 ± 0.01	14.1
	G	82 ± 27.5	0.21 ± 0.03	
	U	64 ± 6.0	0.20 ± 0.02	
C2498	A	66 ± 2.9	0.41 ± 0.14	17
C2499	A	82 ± 5.9	0.15 ± 0.01	14.7
	G	71 ± 26.4	0.21 ± 0.01	
	U	80 ± 25.3	0.22 ± 0.02	
U2500	A	59 ± 12.8	0.24 ± 0.08	12.7
	G	25 ± 0.9	0.37 ± 0.02	
C2501	U	45 ± 16.2	0.43 ± 0.20	10.2
G2502	A	72 ± 21.4	0.25 ± 0.04	16.7
	U	68 ± 26.1	0.27 ± 0.01	
A2503	G	73 ± 7.1	0.35 ± 0.01	11.2
	U	54 ± 4.7	0.22 ± 0.01	
U2504	C	44 ± 16.8	0.50 ± 0.03	10.5
	G	59 ± 23.4	0.21 ± 0.02	
G2505	A	70 ± 21.7	0.20 ± 0.01	8.4
U2506	G	52 ± 2.8	0.43 ± 0.11	6.9
C2507	G	66 ± 6.3	0.21 ± 0.09	10.9
	U	32 ± 1.2	0.56 ± 0.01	
U2552	A	89 ± 3.0	0.17 ± 0.01	21.6
	C	99 ± 4.2	0.14 ± 0.01	
	G	81 ± 2.3	0.16 ± 0.01	
G2553	C	22 ± 4.7	0.81 ± 0.13	14.5
U2554	A	58 ± 6.9	0.24 ± 0.01	17.3
	C	72 ± 1.0	0.12 ± 0.01	
	G	59 ± 0.5	0.20 ± 0.01	
G2582	A	42 ± 21.2	0.69 ± 0.15	15.4
	C	52 ± 2.4	0.40 ± 0.03	
	U	43 ± 15.5	0.24 ± 0.01	
G2583	A	76 ± 21.5	0.22 ± 0.02	9.4
	C	28 ± 10.4	0.52 ± 0.05	
	U	59 ± 3.6	0.31 ± 0.02	
U2584	A	29 ± 10.8	0.55 ± 0.26	7.3
	C	70 ± 24.3	0.24 ± 0.01	

	G	36 ± 2.0	0.58 ± 0.04	
U2585	A	42 ± 4.2	0.49 ± 0.03	5.4
	C	93 ± 0.4	0.55 ± 0.05	
U2586	A	81 ± 24.1	0.18 ± 0.01	10.2
	C	83 ± 25.9	0.18 ± 0.01	
	G	80 ± 1.7	0.15 ± 0.01	
A2587	C	68 ± 12.2	0.24 ± 0.02	14.8
	U	76 ± 0.9	0.15 ± 0.01	
G2588	A	85 ± 32.2	0.18 ± 0.01	19.3
	C	70 ± 20.9	0.21 ± 0.01	
	U	68 ± 22.9	0.20 ± 0.01	
A2602	C	13 ± 0.7	0.79 ± 0.04	6.6
	G	13 ± 0.2	0.68 ± 0.10	
	U	19 ± 1.0	0.99 ± 0.07	
C2606	A	89 ± 23.2	0.13 ± 0.01	19.6
	G	84 ± 28.2	0.20 ± 0.01	
	U	76 ± 16.9	0.16 ± 0.02	
G2607	A	81 ± 0.5	0.15 ± 0.01	17.3
	C	84 ± 2.4	0.17 ± 0.02	
	U	81 ± 1.4	0.16 ± 0.01	
G2608	A	59 ± 20.3	0.21 ± 0.01	15.1
	C	41 ± 0.7	0.30 ± 0.01	
	U	65 ± 20.1	0.20 ± 0.01	
U2609	A	88 ± 17.4	0.39 ± 0.01	18
	C	93 ± 16.0	0.32 ± 0.01	
	G	83 ± 4.6	0.40 ± 0.03	
C2610	G	81 ± 0.4	0.16 ± 0.01	14.3
	U	88 ± 27.6	0.15 ± 0.01	
C2611	G	97 ± 3.7	0.55 ± 0.04	17.7
	U	99 ± 5.2	0.42 ± 0.01	

[a] Translation of the *gfp-tnaC* (W12R) mutant relative to its translation in the OSYRIS cells expressing unmutated oRbs

[b] Distance from α-amino group of the amino acid esterifying the A-site tRNA

Supplementary Table 2. Genotypes of *E. coli* strains used in this study.

Strain	Genotype	Reference
SQ171 (FG)	*ilvG rfb-50 rph-1 ΔrrnGADEHBC / ptRNA67, pA15, SpcR, alaT, aspT, ileT, trpT, gltT, yebX, rpsA A549V*	[1,3]
JM109	*F´ traD36 proA+B+ lacIq Δ(lacZ)M15/ Δ(lac-proAB) glnV44 e14- gyrA96 recA1 relA1 endA1 thi hsdR17*	[12]
POP2136	*F- supE44 hsdR17 mcrA+ mcrB+ endA1 thi-1 aroB mal- cI857 lambda PR tetR lambda*	[7]
BW25113	Δ(*araD-araB*)*567* Δ(*rhaD-rhaB*)*568 ΔlacZ4787* (::rrnB-3) *hsdR514 rph-1*	[13]

Supplementary Table 3. The genotype of the OSYRIS cells.

Gene name	Mutation	Function of the gene
Mutations present in the Ribo-T cells (the fast-growing, FG, phenotype) [a)1]		
rpsA	Ala548Val	30S ribosomal subunit protein S1
ybeX	Leu22 -> stop codon	Putative Mg^{2+}/Co^{2+} efflux transporter
Mutations deliberately introduced during construction of the OSYRIS cells		
upp	Deletion of the gene	uracil phosphoribosyltransferase
recA	Deletion of the gene	DNA recombination/repair protein
Spontaneous mutations acquired during OSYRIS development		
yaiW	Glu292Gly	surface-exposed outer membrane lipoprotein
cusA	Glu512 -> stop codon	copper/silver export system permease
dhaK	Single nucleotide deletion in the promoter	dihydroxyacetone kinase subunit K
ackA	Thr151Pro	acetate kinase
ptsI	Gly452Ala	phosphoenolpyruvate-protein phosphotransferase

[a)] Genotype of the original SQ171 host derived from MG1655: *ilvG rfb-50 rph-1 ΔrrnGADEHBC*[3]

Supplementary Table 4. Primers used in the study.

Primer	Sequence [a)]
NA1	AATAGGGGTTCCGCGCACATTTCCCGGCGCGTAAAACAAAAGGCCCAGTCTTCCG
NA2	ACTCTTCCTGTCGTCATATCTACAAGCCGGCTTTCCTTTCCATCAAAAAAATATTGATGAAATGA
NA3	TATACGAGCCGATGATTAATTGTCA
NA4	GCCTGGCGGCAGTAGCGC
NA5	AATCATGGCAATTCTGGAAGAAATAGCGCGGCCGCGATCTCTCACCTACCAAACAAT
NA6	CTGCAGGTCGACGGATCCCCGGAATGGCGCGCCGGCTTGTAGATATGACGACAGGAAGAG
NA7	TCCTGTCGTCATATCTACAAGCCGGCGCGCCATTCCGGGGATCCGTCGACC
NA8	GTCTGACCACTTCGGATTATCCCGCATCGATTGTAGGCTGGAGCTGCTTCG
NA9	AACTTCGAAGCAGCTCCAGCCTACAATCGATGCGGGATAATCCGAAGTGGTCAGACTGGA
NA10	ATTGTTTGGTAGGTGAGAGATCGCGGCCGCGCTATTTCTTCCAGAATTGCCATGA
NA11	CAAGAAAATGGTTTGTTATAGTCGAATAAACTATATCTGTTATTTTTTCCAACCACAGAT
NA12	GAAAGAAGAAAAAACCTAAATAACGCCCCACAGCTCATTTCATCAATATTTTTTTGATGG
NA13	ATAGTTGGCGAAGTAATCGCAACATATTCGCGGCCGCTTCTAGAGTCCCT
NA14	ATCTGTGGTTGGAAAAAATAACAGATATAGTTTATTCGACTATAACAAACCATTTTCTTG
NA15	ACCCTCACTGATCCGCATGGGGCCCCCGTTCCATACAGAAGCTGGGCGAA
NA16	AGGGACTCTAGAAGCGGCCGCGAATATGTTGCGATTACTTCGCCAACTAT
NA17	TTCGCCCAGCTTCTGTATGGAACGGGGGCCCCATGCGGATCAGTGAGGGT
NA18	CCATCAAAAAAATATTGATGAAATGAGCTGTGGGGCGTTATTTAGGTTTTTTCTTCTTTC
NA19	AAACTATCAGGTCAAGTCTGCTTTTATTAT
NA20	CGAGTAAATAATATTCACAATGTACCATT
NA21	CTGTTATTTTTTCCAACCACAGATCTATGGAAGACGCCAAAAACATAAAGAA
NA22	GCGGCCGCGGGCTTTGTTAGCAGCCGGTCGACTTACAATTTGGACTTTCCGCCCTTC
NA23	TCGTCGAGATCGATCTTCAGCGGCCGCGGGCTTTGTTAGCAGCCGGTCGACTCAAGGGCGGTGATCGACAATTTTG
NA24	TATCTGTTATTTTTTCCAACCACAGATCTATGAGCAAAGGTGAAGAACTGTTTAC
NA25	CAAAATTGTCGATCACCGCCCTTGA
NA26	ACGTTCAAATCCGCTCCCGG
NA27	CAGAACATATTGACTATCCGGTATTACCCGGCATGACAGGAGTAAAAATGGTGTAGGCTGGAGCTGCTTC
NA28	ATGCGACCCTTGTGTATCAAACAAGACGATTAAAAATCTTCGTTAGTTTCATGGGAATTAGCCATGGTCC
NA29	TCGATTTTTGTGATGCTCGTCAGGG
NA30	ATCGACAATTTTGTTGTCAATATTGAACC***A***TTTATGGCTCCACGTAATACCTGCC
NA31	GACAATTTTGTTGTCAATATTGAACC***G***TTTATGGCTCCACGTAATACCTGCC
NA32	GTGAATGCGGCAGGTATTACGTGGAGCCATAAATGGTTCAATATTGACAACAAAATTGTC
NA33	GTGAATGCGGCAGGTATTACGTGGAGCCATAAACGGTTCAATATTGACAACAAAATTGTC
NA34	CTAAAACAAGTAAATAAACGAGAGATGACC
NA35	CAGCACTGTGCAAACACGAAAGTGGACGTATACGGTGTGACGCCTGCCCG
NA36	TGCAGGTCGACGGATCCCCGGAATGGCGCGTATTCGAGCCGGATGAGTAATTGTCAATTT

[a)] The sequences complementary to the target are underlined

Supplementary Table 5. Primers and dNTP/ddNTP combinations used for the primer extension analysis.

23S rRNA mutation	Primer sequence	ddNTP	dNTP
A2058G	GTAAAGGTTCACGGGGTC	ddCTP	dATP, dGTP, dTTP
A2451C	CTCTTGGGCGGTATCAGCCT	ddTTP	dATP, dGTP, dCTP
G2553C	CGTACCACTTTAAATGGCG	ddCTP	dATP, dGTP, dTTP
A2602U	CACGGCAGATAGGGACCGAA	ddTTP	dATP, dGTP, dCTP
A2060C	AAGCTATAGTAAAGGTTCACGGGGT	ddTTP	dATP, dGTP, dCTP
A2062G	TGTCAAGCTATAGTAAAGGTTCACG	ddTTP	dATP, dGTP, dCTP
A2450U	TGAACTCTTGGGCGGTATCAGCCTG	ddATP	dGTP, dCTP, dTTP
U2500G	CCCCAGGATGTGATGAGCCGACATC	ddATP	dGTP, dCTP, dTTP
A2503G	CCAGGATGTGATGAGCCGAC	ddTTP	dATP, dGTP, dCTP
C2611U	TTCTCCAGCGCCCACGGCAGATA	ddATP	dGTP, dCTP, dTTP
C2611G	TTCTCCAGCGCCCACGGCAGATA	ddCTP	dATP, dGTP, dTTP

Supplementary references

1 Orelle, C. *et al.* Protein synthesis by ribosomes with tethered subunits. *Nature* **524**, 119-124 (2015).

2 Carlson, E. D. *et al.* Engineered ribosomes with tethered subunits for expanding biological function. *Nat. Commun.* **10**, 3920 (2019).

3 Quan, S., Skovgaard, O., McLaughlin, R. E., Buurman, E. T. & Squires, C. L. Markerless *Escherichia coli rrn* deletion strains for genetic determination of ribosomal binding sites. *G3* **5**, 2555-2557 (2015).

4 Keseler, I. M. *et al.* The EcoCyc database: reflecting new knowledge about Escherichia coli K-12. *Nucleic Acids Res.* **45**, D543-D550 (2017).

5 Polikanov, Y. S., Steitz, T. A. & Innis, C. A. A proton wire to couple aminoacyl-tRNA accommodation and peptide-bond formation on the ribosome. *Nat. Struct. Molec. Biol.* **21**, 787-793 (2014).

6 Sato, N. S., Hirabayashi, N., Agmon, I., Yonath, A. & Suzuki, T. Comprehensive genetic selection revealed essential bases in the peptidyl-transferase center. *Proc. Natl. Acad. Sci. USA* **103**, 15386-15391 (2006).

7 Kusters, J. G., Jager, E. J. & van der Zeijst, B. A. Improvement of the cloning linker of the bacterial expression vector pEX. *Nucleic Acids Res.* **17**, 8007 (1989).

8 Gong, F. & Yanofsky, C. Instruction of translating ribosome by nascent peptide. *Science* **297**, 1864-1867 (2002).

9 Cruz-Vera, L. R., Rajagopal, S., Squires, C. & Yanofsky, C. Features of ribosome-peptidyl-tRNA interactions essential for tryptophan induction of *tna* operon expression. *Mol. Cell* **19**, 333-343 (2005).

10 Vazquez-Laslop, N., Ramu, H., Klepacki, D., Kannan, K., Mankin, A. S. The key role of a conserved and modified rRNA residue in the ribosomal response to the nascent peptide. *EMBO J.* **29**, 3108-3117 (2010).

11 Heacock, D., Forsyth, C. J., Shiba, K. & MusierForsyth, K. Synthesis and aminoacyl-tRNA synthetase inhibitory activity of prolyl adenylate analogs. *Bioorg. Chem.* **24**, 273-289 (1996).

12 Yanisch-Perron, C., Vieira, J. & Messing, J. Improved M13 phage cloning vectors and host strains: nucleotide sequences of the M13mp18 and pUC19 vectors. *Gene* **33**, 103-119 (1985).

13 Datsenko, K. A. & Wanner, B. L. One-step inactivation of chromosomal genes in *Escherichia coli* K-12 using PCR products. *Proc. Natl. Acad. Sci. USA* **97**, 6640-6645 (2000).
