# Supplementary material for: A fully orthogonal system for protein synthesis in bacterial cells
Source: Nat Commun. 2020 Apr 20;11:1858. doi: 10.1038/s41467-020-15756-1 (PMC7170887; doi:10.1038/s41467-020-15756-1)
Supplement: Supplementary file 3 — Supplementary Data 1 [file 41467_2020_15756_MOESM3_ESM.pdf]

pRibo-Tt

LOCUS Exported 8188 bp ds-DNA circular SYN 17-JUN-2019
DEFINITION synthetic circular DNA
ACCESSION .
VERSION .
KEYWORDS .
SOURCE synthetic DNA construct
  ORGANISM synthetic DNA construct
REFERENCE 1 (bases 1 to 8188)
AUTHORS Alexander S. Mankin
TITLE Direct Submission
  JOURNAL Exported Monday, Jun 17, 2019 from SnapGene 4.3.10
  https://www.snapgene.com
FEATURES Location/Qualifiers
  source 1..8188
    /organism="synthetic DNA construct"
    /mol_type="other DNA"
  promoter 9..148
    /locus_tag="pL"
    /label=pL
  -35_signal 107..112
    /locus_tag="-35"
    /label=-35
  -10_signal 130..135
    /locus_tag="-10"
    /label=-10
  precursor_RNA 181..319
    /locus_tag="16S 5' precursor"
    /label=16S 5' precursor
  rRNA 320..1768
    /locus_tag="16S rRNA (5')"
    /label=16S rRNA (5')
  rRNA 1767..1781
    /locus_tag="Linker 1"
    /label=Linker 1
  rRNA 1782..4685
    /locus_tag="cp23S"
    /label=cp23S
  rRNA 1826..1829
    /locus_tag="cp linker (GAGA)"
    /label=cp linker (GAGA)
  misc_feature 3887
    /locus_tag="A2058G, Ery resistance"
    /label=A2058G, Ery resistance
  rRNA 4686..4695
    /locus_tag="Linker 2"
    /label=Linker 2
  rRNA 4696..4784
    /locus_tag="16S rRNA (3')"
    /label=16S rRNA (3')
  RBS 4776..4784
    /label=Shine-Dalgarno sequence
    /note="full consensus sequence for ribosome-binding sites
    upstream of start codons in E. coli; complementary to a

```
                 region in the 3' end of the 16S rRNA (Chen et al., 1994)"
     precursor_RNA   4785..4841
                 /locus_tag="16S 3' precursor"
                 /label=16S 3' precursor
     tRNA            4956..5031
                 /locus_tag="Glu-tRNA"
                 /label=Glu-tRNA
     rRNA            5328..5446
                 /locus_tag="5S rRNA"
                 /label=5S rRNA
     terminator      5447..5533
                 /gene="Escherichia coli rrnB"
                 /label=rrnB T1 terminator
                 /note="transcription terminator T1 from the E. coli rrnB
                 gene"
     terminator      5625..5652
                 /label=rrnB T2 terminator
                 /note="transcription terminator T2 from the E. coli rrnB
                 gene"
     promoter        5735..5763
                 /label=tac promoter
                 /note="strong E. coli promoter; hybrid between the trp and
                 lac UV5 promoters"
     tRNA            5837..5913
                 /locus_tag="tRNA Asp"
                 /label=tRNA Asp
     tRNA            5922..5997
                 /locus_tag="tRNA Trp"
                 /label=tRNA Trp
     tRNA            6036..6112
                 /locus_tag="tRNA Ile"
                 /label=tRNA Ile
     tRNA            6155..6230
                 /locus_tag="tRNA Ala"
                 /label=tRNA Ala
     tRNA            6269..6344
                 /locus_tag="tRNA Glu"
                 /label=tRNA Glu
     terminator      6361..6403
                 /locus_tag="T1"
                 /label=T1
     promoter        6420..6524
                 /gene="bla"
                 /label=AmpR promoter
     CDS             6525..7385
                 /codon_start=1
                 /gene="bla"
                 /product="beta-lactamase"
                 /label=AmpR
                 /note="confers resistance to ampicillin, carbenicillin, and
                 related antibiotics"
                 /translation="MSIQHFRVALIPFFAAFCLPVFAHPETLVKVKDAEDQLGARVGYI
                 ELDLNSGKILESFRPEERFPMMSTFKVLLCGAVLSRVDAGQEQLGRRIHYSQNDLVEYS
                 PVTEKHLTDGMTVRELCSAAITMSDNTAANLLLTTIGGPKELTAFLHNMGDHVTRLDRW
                 EPELNEAIPNDERDTTMPAAMATTLRKLLTGELLTLASRQQLIDWMEADKVAGPLLRSA
                 LPASWFIADKSGAGERGSRGIIAALGPDGKPSRIVVIYTTGSQATMDERNRQIAEIGAS
```

```
                     LIKHW"
     rep_origin      7555..8143
                     /direction=RIGHT
                     /label=ori
                     /note="high-copy-number ColE1/pMB1/pBR322/pUC origin of
                     replication"
ORIGIN
        1 GCGGCCGCGA TCTCTCACCT ACCAAACAAT GCCCCCCTGC AAAAAATAAA TTCATATAAA
       61 AAACATACAG ATAACCATCT GCGGTGATAA ATTATCTCTG GCGGTGTTGA CATAAATACC
      121 ACTGGCGGTG ATACTGAGCA CGGGTACCGG CCGCTGAGAA AAAGCGAAGC GGCACTGCTC
      181 TTTAACAATT TATCAGACAA TCTGTGTGGG CACTCGAAGA TACGGATTCT TAACGTCGCA
      241 AGACGAAAAA TGAATACCAA GTCTCAAGAG TGAACACGTA ATTCATTACG AAGTTTAATT
      301 CTTTGAGCGT CAAACTTTTA AATTGAAGAG TTTGATCATG GCTCAGATTG AACGCTGGCG
      361 GCAGGCCTAA CACATGCAAG TCGAACGGTA ACAGGAAGAA GCTTGCTTCT TTGCTGACGA
      421 GTGGCGGACG GGTGAGTAAT GTCTGGGAAA CTGCCTGATG GAGGGGGATA ACTACTGGAA
      481 ACGGTAGCTA ATACCGCATA ACGTCGCAAG ACCAAAGAGG GGGACCTTCG GGCCTCTTGC
      541 CATCGGATGT GCCCAGATGG GATTAGCTAG TAGGTGGGGT AACGGCTCAC CTAGGCGACG
      601 ATCCCTAGCT GGTCTGAGAG GATGACCAGC CACACTGGAA CTGAGACACG GTCCAGACTC
      661 CTACGGGAGG CAGCAGTGGG GAATATTGCA CAATGGGCGC AAGCCTGATG CAGCCATGCC
      721 GCGTGTATGA AGAAGGCCTT CGGGTTGTAA AGTACTTTCA GCGGGGAGGA AGGGAGTAAA
      781 GTTAATACCT TTGCTCATTG ACGTTACCCG CAGAAGAAGC ACCGGCTAAC TCCGTGCCAG
      841 CAGCCGCGGT AATACGGAGG GTGCAAGCGT TAATCGGAAT TACTGGGCGT AAAGCGCACG
      901 CAGGCGGTTT GTTAAGTCAG ATGTGAAATC CCCGGGCTCA ACCTGGGAAC TGCATCTGAT
      961 ACTGGCAAGC TTGAGTCTCG TAGAGGGGGG TAGAATTCCA GGTGTAGCGG TGAAATGCGT
     1021 AGAGATCTGG AGGAATACCG GTGGCGAAGG CGGCCCCCTG GACGAAGACT GACGCTCAGG
     1081 TGCGAAAGCG TGGGGAGCAA ACAGGATTAG ATACCCTGGT AGTCCACGCC GTAAACGATG
     1141 TCGACTTGGA GGTTGTGCCC TTGAGGCGTG GCTTCCGGAG CTAACGCGTT AAGTCGACCG
     1201 CCTGGGGAGT ACGGCCGCAA GGTTAAAACT CAAATGAATT GACGGGGGCC CGCACAAGCG
     1261 GTGGAGCATG TGGTTTAATT CGATGCAACG CGAAGAACCT TACCTGGTCT TGACATCCAC
     1321 GGAAGTTTTC AGAGATGAGA ATGTGCCTTC GGGAACCGTG AGACAGGTGC TGCATGGCTG
     1381 TCGTCAGCTC GTGTTGTGAA ATGTTGGGTT AAGTCCCGCA ACGAGCGCAA CCCTTATCCT
     1441 TTGTTGCCAG CGGTCCGGCC GGGAACTCAA AGGAGACTGC CAGTGATAAA CTGGAGGAAG
     1501 GTGGGGATGA CGTCAAGTCA TCATGGCCCT TACGACCAGG GCTACACACG TGCTACAATG
     1561 GCGCATACAA AGAGAAGCGA CCTCGCGAGA GCAAGCGGAC CTCATAAAGT GCGTCGTAGT
     1621 CCGGATTGGA GTCTGCAACT CGACTCCATG AAGTCGGAAT CGCTAGTAAT CGTGGATCAG
     1681 AATGCCACGG TGAATACGTT CCCGGGCCTT GTACACACCG CCCGTCACAC CATGGGAGTG
     1741 GGTTGCAAAA GAAGTAGGTA GCTTAACCCA ATGAACAATT GGATGCGTTG AGCTAACCGG
     1801 TACTAATGAA CCGTGAGGCT TAACCGAGAG GTTAAGCGAC TAAGCGTACA CGGTGGATGC
     1861 CCTGGCAGTC AGAGGCGATG AAGGACGTGC TAATCTGCGA TAAGCGTCGG TAAGGTGATA
     1921 TGAACCGTTA TAACCGGCGA TTTCCGAATG GGGAAACCCA GTGTGTTTCG ACACACTATC
     1981 ATTAACTGAA TCCATAGGTT AATGAGGCGA ACCGGGGGAA CTGAAACATC TAAGTACCCC
     2041 GAGGAAAAGA AATCAACCGA GATTCCCCCA GTAGCGGCGA GCGAACGGGG AGCAGCCCAG
     2101 AGCCTGAATC AGTGTGTGTG TTAGTGGAAG CGTCTGGAAA GGCGCGCGAT ACAGGGTGAC
     2161 AGCCCCGTAC ACAAAAATGC ACATGCTGTG AGCTCGATGA GTAGGGCGGG ACACGTGGTA
     2221 TCCTGTCTGA ATATGGGGGG ACCATCCTCC AAGGCTAAAT ACTCCTGACT GACCGATAGT
     2281 GAACCAGTAC CGTGAGGGAA AGGCGAAAAG AACCCCGGCG AGGGGAGTGA AAAAGAACCT
     2341 GAAACCGTGT ACGTACAAGC AGTGGGAGCA CGCTTAGGCG TGTGACTGCG TACCTTTTGT
     2401 ATAATGGGTC AGCGACTTAT ATTCTGTAGC AAGGTTAACC GAATAGGGGA GCCGAAGGGA
     2461 AACCGAGTCT TAACTGGGCG TTAAGTTGCA GGGTATAGAC CCGAAACCCG GTGATCTAGC
     2521 CATGGGCAGG TTGAAGGTTG GGTAACACTA ACTGGAGGAC CGAACCGACT AATGTTGAAA
     2581 AATTAGCGGA TGACTTGTGG CTGGGGGTGA AAGGCCAATC AAACCGGGAG ATAGCTGGTT
     2641 CTCCCCGAAA GCTATTTAGG TAGCGCCTCG TGAATTCATC TCCGGGGGTA GAGCACTGTT
     2701 TCGGCAAGGG GGTCATCCCG ACTTACCAAC CCGATGCAAA CTGCGAATAC CGGAGAATGT
     2761 TATCACGGGA GACACACGGC GGGTGCTAAC GTCCGTCGTG AAGAGGGAAA CAACCCAGAC
     2821 CGCCAGCTAA GGTCCCAAAG TCATGGTTAA GTGGGAAACG ATGTGGGAAG GCCCAGACAG
     2881 CCAGGATGTT GGCTTAGAAG CAGCCATCAT TTAAAGAAAG CGTAATAGCT CACTGGTCGA
```

```
2941 GTCGGCCTGC GCGGAAGATG TAACGGGGCT AAACCATGCA CCGAAGCTGC GGCAGCGACG
3001 CTTATGCGTT GTTGGGTAGG GGAGCGTTCT GTAAGCCTGC GAAGGTGTGC TGTGAGGCAT
3061 GCTGGAGGTA TCAGAAGTGC GAATGCTGAC ATAAGTAACG ATAAAGCGGG TGAAAAGCCC
3121 GCTCGCCGGA AGACCAAGGG TTCCTGTCCA ACGTTAATCG GGGCAGGGTG AGTCGACCCC
3181 TAAGGCGAGG CCGAAAGGCG TAGTCGATGG GAAACAGGTT AATATTCCTG TACTTGGTGT
3241 TACTGCGAAG GGGGGACGGA GAAGGCTATG TTGGCCGGGC GACGGTTGTC CCGGTTTAAG
3301 CGTGTAGGCT GGTTTTCCAG GCAAATCCGG AAAATCAAGG CTGAGGCGTG ATGACGAGGC
3361 ACTACGGTGC TGAAGCAACA AATGCCCTGC TTCCAGGAAA AGCCTCTAAG CATCAGGTAA
3421 CATCAAATCG TACCCCAAAC CGACACAGGT GGTCAGGTAG AGAATACCAA GGCGCTTGAG
3481 AGAACTCGGG TGAAGGAACT AGGCAAAATG GTGCCGTAAC TTCGGGAGAA GGCACGCTGA
3541 TATGTAGGTG AGGTCCCTCG CGGATGGAGC TGAAATCAGT CGAAGATACC AGCTGGCTGC
3601 AACTGTTTAT TAAAAACACA GCACTGTGCA AACACGAAAG TGGACGTATA CGGTGTGACG
3661 CCTGCCCGGT GCCGGAAGGT TAATTGATGG GGTTAGCGCA AGCGAAGCTC TTGATCGAAG
3721 CCCCGGTAAA CGGCGGCCGT AACTATAACG GTCCTAAGGT AGCGAAATTC CTTGTCGGGT
3781 AAGTTCCGAC CTGCACGAAT GGCGTAATGA TGGCCAGGCT GTCTCCACCC GAGACTCAGT
3841 GAAATTGAAC TCGCTGTGAA GATGCAGTGT ACCCGCGGCA AGACGGAAAG ACCCCGTGAA
3901 CCTTTACTAT AGCTTGACAC TGAACATTGA GCCTTGATGT GTAGGATAGG TGGGAGGCTT
3961 TGAAGTGTGG ACGCCAGTCT GCATGGAGCC GACCTTGAAA TACCACCCTT TAATGTTTGA
4021 TGTTCTAACG TTGACCCGTA ATCCGGGTTG CGGACAGTGT CTGGTGGGTA GTTTGACTGG
4081 GGCGGTCTCC TCCTAAAGAG TAACGGAGGA GCACGAAGGT TGGCTAATCC TGGTCGGACA
4141 TCAGGAGGTT AGTGCAATGG CATAAGCCAG CTTGACTGCG AGCGTGACGG CGCGAGCAGG
4201 TGCGAAAGCA GGTCATAGTG ATCCGGTGGT TCTGAATGGA AGGGCCATCG CTCAACGGAT
4261 AAAAGGTACT CCGGGGATAA CAGGCTGATA CCGCCCAAGA GTTCATATCG ACGGCGGTGT
4321 TTGGCACCTC GATGTCGGCT CATCACATCC TGGGGCTGAA GTAGGTCCCA AGGGTATGGC
4381 TGTTCGCCAT TTAAAGTGGT ACGCGAGCTG GGTTTAGAAC GTCGTGAGAC AGTTCGGTCC
4441 CTATCTGCCG TGGGCGCTGG AGAACTGAGG GGGGCTGCTC CTAGTACGAG AGGACCGGAG
4501 TGGACGCATC ACTGGTGTTC GGGTTGTCAT GCCAATGGCA CTGCCCGGTA GCTAAATGCG
4561 GAAGAGATAA GTGCTGAAAG CATCTAAGCA CGAAACTTGC CCCGAGATGA GTTCTCCCTG
4621 ACCCTTTAAG GGTCCTGAAG GAACGTTGAA GACGACGACG TTGATAGGCC GGGTGTGTAA
4681 GCGCAGATAA CTAGTGGAGG GCGCTTACCA CTTTGTGATT CATGACTGGG GTGAAGTCGT
4741 AACAAGGTAA CCGTAGGGGA ACCTGCGGTT GGATCACCTC CTTACCTTAA AGAAGCGTAC
4801 TTTGTAGTGC TCACACAGAT TGTCTGATAG AAAGTGAAAA GCAAGGCGTT TACGCGTTGG
4861 GAGTGAGGCT GAAGAGAATA AGGCCGTTCG CTTTCTATTA ATGAAAGCTC ACCCTACACG
4921 AAAATATCAC GCAACGCGTG ATAAGCAATT TTCGTGTCCC CTTCGTCTAG AGGCCCAGGA
4981 CACCGCCCTT TCACGGCGGT AACAGGGGTT CGAATCCCCT AGGGGACGCC ACTTGCTGGT
5041 TTGTGAGTGA AAGTCGCCGA CCTTAATATC TCAAAACTCA TCTTCGGGTG ATGTTTGAGA
5101 TATTTGCTCT TTAAAAATCT GGATCAAGCT GAAAATTGAA ACACTGAACA ACGAGAGTTG
5161 TTCGTGAGTC TCTCAAATTT TCGCAACACG ATGATGAATC GAAAGAAACA TCTTCGGGTT
5221 GTGAGCTTAA GCTTACAACG CCGAAGCTGT TTTGGCGGAT GAGAGAAGAT TTTCAGCCTG
5281 ATACAGATTA AATCAGAACG CAGAAGCGGT CTGATAAAAC AGAATTTGCC TGGCGGCAGT
5341 AGCGCGGTGG TCCCACCTGA CCCCATGCCG AACTCAGAAG TGAAACGCCG TAGCGCCGAT
5401 GGTAGTGTGG GGTCTCCCCA TGCGAGAGTA GGGAACTGCC AGGCATCAAA TAAAACGAAA
5461 GGCTCAGTCG AAAGACTGGG CCTTTCGTTT TATCTGTTGT TTGTCGGTGA ACGCTCTCCT
5521 GAGTAGGACA AATCCGCCGG GAGCGGATTT GAACGTTGCG AAGCAACGGC CCGGAGGGTG
5581 GCGGGCAGGA CGCCCGCCAT AAACTGCCAG GCATCAAATT AAGCAGAAGG CCATCCTGAC
5641 GGATGGCCTT TTTGCGTTTC TACAAACTCT TCCTGTCGTC ATATCTACAA GCCGGCTTTC
5701 CTTTCCATCA AAAAAATATT GATGAAATGA GCTGTTGACA ATTAATCATC GGCTCGTATA
5761 ATGTGTGGAA TTGTCACACA GGAAACAGAA TTCCCGGGGA TCTGGGGGAT CATCGATGGT
5821 TGTAAAAGAA TTCGGTGGAG CGGTAGTTCA GTCGGTTAGA ATACCTGCCT GTCACGCAGG
5881 GGGTCGCGGG TTCGAGTCCC GTCCGTTCCG CCACCCTAAT TAGGGGCGTA GTTCAATTGG
5941 TAGAGCACCG GTCTCCAAAA CCGGGTGTTG GGAGTTCGAG TCTCTCCGCC CCTGCCAGAA
6001 ATCATCCTTG TCGATGGGAG CAGTAAAACC TCTACAGGCT TGTAGCTCAG GTGGTTAGAG
6061 CGCACCCCTG ATAAGGGTGA GGTCGGTGGT TCAAGTCCAC TCAGGCCTAC CAAATTTGCA
6121 CGGCAAATTT GAAGAGGTTT TAACTACATG TTATGGGGCT ATAGCTCAGC TGGGAGAGCG
6181 CCTGCTTTGC ACGCAGGAGG TCTGCGGTTC GATCCCGCAT AGCTCCACCA TCTCTGTAGT
6241 GATTAAGAGC GTGATAAGCA ATTTTCGTGT CCCCTTCGTC TAGAGGCCCA GGACACCGCC
6301 CTTTCACGGC GGTAACAGGG GTTCGAATCC CCTAGGGGAC GCCACTCTAG GAAATCCGCC
```

```
6361 ATAAAACAAA AGGCTCAGTC GGAAGACTGG GCCTTTTGTT TTACGCGCCG GGAAATGTGC
6421 GCGGAACCCC TATTTGTTTA TTTTTCTAAA TACATTCAAA TATGTATCCG CTCATGAGAC
6481 AATAACCCTG ATAAATGCTT CAATAATATT GAAAAAGGAA GAGTATGAGT ATTCAACATT
6541 TCCGTGTCGC CCTTATTCCC TTTTTTGCGG CATTTTGCCT TCCTGTTTTT GCTCACCCAG
6601 AAACGCTGGT GAAAGTAAAA GATGCTGAAG ATCAGTTGGG TGCACGAGTG GGTTACATCG
6661 AACTGGATCT CAACAGCGGT AAGATCCTTG AGAGTTTTCG CCCCGAAGAA CGTTTTCCAA
6721 TGATGAGCAC TTTTAAAGTT CTGCTATGTG GCGCGGTATT ATCCCGTGTT GACGCCGGGC
6781 AAGAGCAACT CGGTCGCCGC ATACACTATT CTCAGAATGA CTTGGTTGAG TACTCACCAG
6841 TCACAGAAAA GCATCTTACG GATGGCATGA CAGTAAGAGA ATTATGCAGT GCTGCAATAA
6901 CCATGAGTGA TAACACTGCG GCCAACTTAC TTCTGACAAC GATCGGAGGA CCGAAGGAGC
6961 TAACCGCTTT TTTGCACAAC ATGGGGGATC ATGTAACTCG CCTTGATCGT TGGGAACCGG
7021 AGCTGAATGA AGCCATACCA AACGACGAGC GTGACACCAC GATGCCTGCA GCAATGGCAA
7081 CAACGTTGCG CAAACTATTA ACTGGCGAAC TACTTACTCT AGCTTCCCGG CAACAATTAA
7141 TAGACTGGAT GGAGGCGGAT AAAGTTGCAG GACCACTTCT GCGCTCGGCC CTTCCGGCTA
7201 GCTGGTTTAT TGCTGATAAA TCTGGAGCCG GTGAGCGTGG GTCTCGCGGT ATCATTGCAG
7261 CACTGGGGCC AGATGGTAAG CCCTCCCGTA TCGTAGTTAT CTACACGACG GGGAGTCAGG
7321 CAACTATGGA TGAACGAAAT AGACAGATCG CTGAGATAGG TGCCTCACTG ATTAAGCATT
7381 GGTAACTGCA GACCAAGTTT ACTCATATAT ACTTTAGATT GATTTAAAAC TTCATTTTTA
7441 ATTTAAAAGG ATCTAGGTGA AGATCCTTTT TGATAATCTC ATGACCAAAA TCCCTTAACG
7501 TGAGTTTTCG TTCCACTGAG CGTCAGACCC CGTAGAAAAG ATCAAAGGAT CTTCTTGAGA
7561 TCCTTTTTTT CTGCGCGTAA TCTGCTGCTT GCAAACAAAA AAACCACCGC TACCAGCGGT
7621 GGTTTGTTTG CCGGATCAAG AGCTACCAAC TCTTTTTCCG AAGGTAACTG GCTTCAGCAG
7681 AGCGCAGATA CCAAATACTG TCCTTCTAGT GTAGCCGTAG TTAGGCCACC ACTTCAAGAA
7741 CTCTGTAGCA CCGCCTACAT ACCTCGCTCT GCTAATCCTG TTACCAGTGG CTGCTGCCAG
7801 TGGCGATAAG TCGTGTCTTA CCGGGTTGGA CTCAAGACGA TAGTTACCGG ATAAGGCGCA
7861 GCGGTCGGGC TGAACGGGGG GTTCGTGCAC ACAGCCCAGC TTGGAGCGAA CGACCTACAC
7921 CGAACTGAGA TACCTACAGC GTGAGCTATG AGAAAGCGCC ACGCTTCCCG AAGGGAGAAA
7981 GGCGGACAGG TATCCGGTAA GCGGCAGGGT CGGAACAGGA GAGCGCACGA GGGAGCTTCC
8041 AGGGGGAAAC GCCTGGTATC TTTATAGTCC TGTCGGGTTT CGCCACCTCT GACTTGAGCG
8101 TCGATTTTTG TGATGCTCGT CAGGGGGGCG GAGCCTATGG AAAAACGCCA GCAACGCGGC
8161 CTTTTTACGG TTCCTGGCCT TTTGCTGG
```

//

poRbs

```
LOCUS       Exported              9029 bp ds-DNA     circular SYN 17-JUN-2019
DEFINITION  synthetic circular DNA
ACCESSION   .
VERSION     .
KEYWORDS    .
SOURCE      synthetic DNA construct
  ORGANISM  synthetic DNA construct
REFERENCE   1  (bases 1 to 9029)
AUTHORS   Alexander S. Mankin
TITLE     Direct Submission
  JOURNAL   Exported Monday, Jun 17, 2019 from SnapGene 4.3.10
            https://www.snapgene.com
FEATURES             Location/Qualifiers
     source          1..9029
                     /organism="synthetic DNA construct"
                     /mol_type="other DNA"
     promoter        9..148
                     /locus_tag="PL"
                     /label=PL
     -35_signal      107..112
                     /locus_tag="-35"
                     /label=-35
     -10_signal      130..135
                     /locus_tag="-10"
                     /label=-10
     precursor_RNA   181..319
                     /locus_tag="16S rRNA precursor"
                     /label=16S rRNA precursor
     rRNA            320..1861
                     /locus_tag="16S rRNA"
                     /label=16S rRNA
     RBS             1853..1861
                     /label=Shine-Dalgarno sequence
                     /note="full consensus sequence for ribosome-binding sites
                     upstream of start codons in E. coli; complementary to a
                     region in the 3' end of the 16S rRNA (Chen et al., 1994)"
     tRNA            2033..2108
                     /locus_tag="Glu tRNA"
                     /label=Glu tRNA
     rRNA            2302..5205
                     /locus_tag="23S rRNA"
                     /label=23S rRNA
     rRNA            5299..5417
                     /locus_tag="5S rRNA"
                     /label=5S rRNA
     terminator      5418..5504
                     /gene="Escherichia coli rrnB"
                     /label=rrnB T1 terminator
                     /note="transcription terminator T1 from the E. coli rrnB
                     gene"
     terminator      5596..5623
                     /label=rrnB T2 terminator
                     /note="transcription terminator T2 from the E. coli rrnB
```

```
                     gene"
     CDS             6102..6896
                     /codon_start=1
                     /gene="aph(3')-II (or nptII)"
                     /product="aminoglycoside phosphotransferase from Tn5"
                     /label=NeoR/KanR
                     /note="confers resistance to neomycin, kanamycin, and G418
                     (Geneticin(R))"
                     /translation="MIEQDGLHAGSPAAWVERLFGYDWAQQTIGCSDAAVFRLSAQGRP
                     VLFVKTDLSGALNELQDEAARLSWLATTGVPCAAVLDVVTEAGRDWLLLGEVPGQDLLS
                     SHLAPAEKVSIMADAMRRLHTLDPATCPFDHQAKHRIERARTRMEAGLVDQDDLDEEHQ
                     GLAPAELFARLKARMPDGEDLVVTHGDACLPNIMVENGRFSGFIDCGRLGVADRYQDIA
                     LATRDIAEELGGEWADRFLVLYGIAAPDSQRIAFYRLLDEFF"
     rep_origin      7387..7609
                     /label=pSC101 ori
                     /note="low-copy replication origin that requires the Rep101
                     protein"
     CDS             7657..8607
                     /codon_start=1
                     /gene="rep101"
                     /product="RepA protein needed for replication with the
                     pSC101 origin"
                     /label=Rep101
                     /translation="MSELVVFKANELAISRYDLTEHETKLILCCVALLNPTIENPTRKE
                     RTVSFTYNQYAQMMNISRENAYGVLAKATRELMTRTVEIRNPLVKGFEIFQWTNYAKFS
                     SEKLELVFSEEILPYLFQLKKFIKYNLEHVKSFENKYSMRIYEWLLKELTQKKTHKANI
                     EISLDEFKFMLMLENNYHEFKRLNQWVLKPISKDLNTYSNMKLVVDKRGRPTDTLIFQV
                     ELDRQMDLVTELENNQIKMNGDKIPTTITSDSYLRNGLRKTLHDALTAKIQLTSFEAKF
                     LSDMQSKHDLNGSFSWLTQKQRTTLENILAKYGRI"
ORIGIN

        1 GCGGCCGCGA TCTCTCACCT ACCAAACAAT GCCCCCCTGC AAAAAATAAA TTCATATAAA
       61 AAACATACAG ATAACCATCT GCGGTGATAA ATTATCTCTG GCGGTGTTGA CATAAATACC
      121 ACTGGCGGTG ATACTGAGCA CGGGTACCGG CCGCTGAGAA AAAGCGAAGC GGCACTGCTC
      181 TTTAACAATT TATCAGACAA TCTGTGTGGG CACTCGAAGA TACGGATTCT TAACGTCGCA
      241 AGACGAAAAA TGAATACCAA GTCTCAAGAG TGAACACGTA ATTCATTACG AAGTTTAATT
      301 CTTTGAGCGT CAAACTTTTA AATTGAAGAG TTTGATCATG GCTCAGATTG AACGCTGGCG
      361 GCAGGCCTAA CACATGCAAG TCGAACGGTA ACAGGAAGAA GCTTGCTTCT TTGCTGACGA
      421 GTGGCGGACG GGTGAGTAAT GTCTGGGAAA CTGCCTGATG GAGGGGGATA ACTACTGGAA
      481 ACGGTAGCTA ATACCGCATA ACGTCGCAAG ACCAAAGAGG GGGACCTTCG GGCCTCTTGC
      541 CATCGGATGT GCCCAGATGG GATTAGCTAG TAGGTGGGGT AACGGCTCAC CTAGGCGACG
      601 ATCCCTAGCT GGTCTGAGAG GATGACCAGC CACACTGGAA CTGAGACACG GTCCAGACTC
      661 CTACGGGAGG CAGCAGTGGG GAATATTGCA CAATGGGCGC AAGCCTGATG CAGCCATGCC
      721 GCGTGTATGA AGAAGGCCTT CGGGTTGTAA AGTACTTTCA GCGGGGAGGA AGGGAGTAAA
      781 GTTAATACCT TTGCTCATTG ACGTTACCCG CAGAAGAAGC ACCGGCTAAC TCCGTGCCAG
      841 CAGCCGCGGT AATACGGAGG GTGCAAGCGT TAATCGGAAT TACTGGGCGT AAAGCGCACG
      901 CAGGCGGTTT GTTAAGTCAG ATGTGAAATC CCCGGGCTCA ACCTGGGAAC TGCATCTGAT
      961 ACTGGCAAGC TTGAGTCTCG TAGAGGGGGG TAGAATTCCA GGTGTAGCGG TGAAATGCGT
     1021 AGAGATCTGG AGGAATACCG GTGGCGAAGG CGGCCCCCTG GACGAAGACT GACGCTCAGG
     1081 TGCGAAAGCG TGGGGAGCAA ACAGGATTAG ATACCCTGGT AGTCCACGCC GTAAACGATG
     1141 TCGACTTGGA GGTTGTGCCC TTGAGGCGTG GCTTCCGGAG CTAACGCGTT AAGTCGACCG
     1201 CCTGGGGAGT ACGGCCGCAA GGTTAAAACT CAAATGAATT GACGGGGGCC CGCACAAGCG
     1261 GTGGAGCATG TGGTTTAATT CGATGCAACG CGAAGAACCT TACCTGGTCT TGACATCCAC
     1321 GGAAGTTTTC AGAGATGAGA ATGTGCCTTC GGGAACCGTG AGACAGGTGC TGCATGGCTG
     1381 TCGTCAGCTC GTGTTGTGAA ATGTTGGGTT AAGTCCCGCA ACGAGCGCAA CCCTTATCCT
     1441 TTGTTGCCAG CGGTCCGGCC GGGAACTCAA AGGAGACTGC CAGTGATAAA CTGGAGGAAG
```

1501 GTGGGGATGA CGTCAAGTCA TCATGGCCCT TACGACCAGG GCTACACACG TGCTACAATG
1561 GCGCATACAA AGAGAAGCGA CCTCGCGAGA GCAAGCGGAC CTCATAAAGT GCGTCGTAGT
1621 CCGGATTGGA GTCTGCAACT CGACTCCATG AAGTCGGAAT CGCTAGTAAT CGTGGATCAG
1681 AATGCCACGG TGAATACGTT CCCGGGCCTT GTACACACCG CCCGTCACAC CATGGGAGTG
1741 GGTTGCAAAA GAAGTAGGTA GCTTAACCTT CGGGAGGGCG CTTACCACTT TGTGATTCAT
1801 GACTGGGGTG AAGTCGTAAC AAGGTAACCG TAGGGGAACC TGCGGTTGGA TCACCTCCTT
1861 ACCTTAAAGA AGCGTACTTT GTAGTGCTCA CACAGATTGT CTGATAGAAA GTGAAAAGCA
1921 AGGCGTTTAC GCGTTGGGAG TGAGGCTGAA GAGAATAAGG CCGTTCGCTT TCTATTAATG
1981 AAAGCTCACC CTACACGAAA ATATCACGCA ACGCGTGATA AGCAATTTTC GTGTCCCCTT
2041 CGTCTAGAGG CCCAGGACAC CGCCCTTTCA CGGCGGTAAC AGGGGTTCGA ATCCCCTAGG
2101 GGACGCCACT TGCTGGTTTG TGAGTGAAAG TCGCCGACCT TAATATCTCA AAACTCATCT
2161 TCGGGTGATG TTTGAGATAT TTGCTCTTTA AAAATCTGGA TCAAGCTGAA AATTGAAACA
2221 CTGAACAACG AGAGTTGTTC GTGAGTCTCT CAAATTTTCG CAACACGATG ATGAATCGAA
2281 AGAAACATCT TCGGGTTGTG AGGTTAAGCG ACTAAGCGTA CACGGTGGAT GCCCTGGCAG
2341 TCAGAGGCGA TGAAGGACGT GCTAATCTGC GATAAGCGTC GGTAAGGTGA TATGAACCGT
2401 TATAACCGGC GATTTCCGAA TGGGGAAACC CAGTGTGTTT CGACACACTA TCATTAACTG
2461 AATCCATAGG TTAATGAGGC GAACCGGGGG AACTGAAACA TCTAAGTACC CCGAGGAAAA
2521 GAAATCAACC GAGATTCCCC CAGTAGCGGC GAGCGAACGG GGAGCAGCCC AGAGCCTGAA
2581 TCAGTGTGTG TGTTAGTGGA AGCGTCTGGA AAGGCGCGCG ATACAGGGTG ACAGCCCCGT
2641 ACACAAAAAT GCACATGCTG TGAGCTCGAT GAGTAGGGCG GGACACGTGG TATCCTGTCT
2701 GAATATGGGG GGACCATCCT CCAAGGCTAA ATACTCCTGA CTGACCGATA GTGAACCAGT
2761 ACCGTGAGGG AAAGGCGAAA AGAACCCCGG CGAGGGGAGT GAAAAAGAAC CTGAAACCGT
2821 GTACGTACAA GCAGTGGGAG CACGCTTAGG CGTGTGACTG CGTACCTTTT GTATAATGGG
2881 TCAGCGACTT ATATTCTGTA GCAAGGTTAA CCGAATAGGG GAGCCGAAGG GAAACCGAGT
2941 CTTAACTGGG CGTTAAGTTG CAGGGTATAG ACCCGAAACC CGGTGATCTA GCCATGGGCA
3001 GGTTGAAGGT TGGGTAACAC TAACTGGAGG ACCGAACCGA CTAATGTTGA AAAATTAGCG
3061 GATGACTTGT GGCTGGGGGT GAAAGGCCAA TCAAACCGGG AGATAGCTGG TTCTCCCCGA
3121 AAGCTATTTA GGTAGCGCCT CGTGAATTCA TCTCCGGGGG TAGAGCACTG TTTCGGCAAG
3181 GGGGTCATCC CGACTTACCA ACCCGATGCA AACTGCGAAT ACCGGAGAAT GTTATCACGG
3241 GAGACACACG GCGGGTGCTA ACGTCCGTCG TGAAGAGGGA AACAACCCAG ACCGCCAGCT
3301 AAGGTCCCAA AGTCATGGTT AAGTGGGAAA CGATGTGGGA AGGCCCAGAC AGCCAGGATG
3361 TTGGCTTAGA AGCAGCCATC ATTTAAAGAA AGCGTAATAG CTCACTGGTC GAGTCGGCCT
3421 GCGCGGAAGA TGTAACGGGG CTAAACCATG CACCGAAGCT GCGGCAGCGA CGCTTATGCG
3481 TTGTTGGGTA GGGGAGCGTT CTGTAAGCCT GCGAAGGTGT GCTGTGAGGC ATGCTGGAGG
3541 TATCAGAAGT GCGAATGCTG ACATAAGTAA CGATAAAGCG GGTGAAAAGC CCGCTCGCCG
3601 GAAGACCAAG GGTTCCTGTC CAACGTTAAT CGGGGCAGGG TGAGTCGACC CCTAAGGCGA
3661 GGCCGAAAGG CGTAGTCGAT GGGAAACAGG TTAATATTCC TGTACTTGGT GTTACTGCGA
3721 AGGGGGGACG GAGAAGGCTA TGTTGGCCGG GCGACGGTTG TCCCGGTTTA AGCGTGTAGG
3781 CTGGTTTTCC AGGCAAATCC GGAAAATCAA GGCTGAGGCG TGATGACGAG GCACTACGGT
3841 GCTGAAGCAA CAAATGCCCT GCTTCCAGGA AAAGCCTCTA AGCATCAGGT AACATCAAAT
3901 CGTACCCCAA ACCGACACAG GTGGTCAGGT AGAGAATACC AAGGCGCTTG AGAGAACTCG
3961 GGTGAAGGAA CTAGGCAAAA TGGTGCCGTA ACTTCGGGAG AAGGCACGCT GATATGTAGG
4021 TGAGGTCCCT CGCGGATGGA GCTGAAATCA GTCGAAGATA CCAGCTGGCT GCAACTGTTT
4081 ATTAAAAACA CAGCACTGTG CAAACACGAA AGTGGACGTA TACGGTGTGA CGCCTGCCCG
4141 GTGCCGGAAG GTTAATTGAT GGGGTTAGCG CAAGCGAAGC TCTTGATCGA AGCCCCGGTA
4201 AACGGCGGCC GTAACTATAA CGGTCCTAAG GTAGCGAAAT TCCTTGTCGG GTAAGTTCCG
4261 ACCTGCACGA ATGGCGTAAT GATGGCCAGG CTGTCTCCAC CCGAGACTCA GTGAAATTGA
4321 ACTCGCTGTG AAGATGCAGT GTACCCGCGG CAAGACGGAA AGACCCCGTG AACCTTTACT
4381 ATAGCTTGAC ACTGAACATT GAGCCTTGAT GTGTAGGATA GGTGGGAGGC TTTGAAGTGT
4441 GGACGCCAGT CTGCATGGAG CCGACCTTGA AATACCACCC TTTAATGTTT GATGTTCTAA
4501 CGTTGACCCG TAATCCGGGT TGCGGACAGT GTCTGGTGGG TAGTTTGACT GGGGCGGTCT
4561 CCTCCTAAAG AGTAACGGAG GAGCACGAAG GTTGGCTAAT CCTGGTCGGA CATCAGGAGG
4621 TTAGTGCAAT GGCATAAGCC AGCTTGACTG CGAGCGTGAC GGCGCGAGCA GGTGCGAAAG
4681 CAGGTCATAG TGATCCGGTG GTTCTGAATG GAAGGGCCAT CGCTCAACGG ATAAAAGGTA
4741 CTCCGGGGAT AACAGGCTGA TACCGCCCAA GAGTTCATAT CGACGGCGGT GTTTGGCACC
4801 TCGATGTCGG CTCATCACAT CCTGGGGCTG AAGTAGGTCC CAAGGGTATG GCTGTTCGCC
4861 ATTTAAAGTG GTACGCGAGC TGGGTTTAGA ACGTCGTGAG ACAGTTCGGT CCCTATCTGC

```
4921 CGTGGGCGCT GGAGAACTGA GGGGGGCTGC TCCTAGTACG AGAGGACCGG AGTGGACGCA
4981 TCACTGGTGT TCGGGTTGTC ATGCCAATGG CACTGCCCGG TAGCTAAATG CGGAAGAGAT
5041 AAGTGCTGAA AGCATCTAAG CACGAAACTT GCCCCGAGAT GAGTTCTCCC TGACCCTTTA
5101 AGGGTCCTGA AGGAACGTTG AAGACGACGA CGTTGATAGG CCGGGTGTGT AAGCGCAGCG
5161 ATGCGTTGAG CTAACCGGTA CTAATGAACC GTGAGGCTTA ACCTTACAAC GCCGAAGCTG
5221 TTTTGGCGGA TGAGAGAAGA TTTTCAGCCT GATACAGATT AAATCAGAAC GCAGAAGCGG
5281 TCTGATAAAA CAGAATTTGC CTGGCGGCAG TAGCGCGGTG GTCCCACCTG ACCCCATGCC
5341 GAACTCAGAA GTGAAACGCC GTAGCGCCGA TGGTAGTGTG GGGTCTCCCC ATGCGAGAGT
5401 AGGGAACTGC CAGGCATCAA ATAAAACGAA AGGCTCAGTC GAAAGACTGG GCCTTTCGTT
5461 TTATCTGTTG TTTGTCGGTG AACGCTCTCC TGAGTAGGAC AAATCCGCCG GGAGCGGATT
5521 TGAACGTTGC GAAGCAACGG CCCGGAGGGT GGCGGGCAGG ACGCCCGCCA TAAACTGCCA
5581 GGCATCAAAT TAAGCAGAAG GCCATCCTGA CGGATGGCCT TTTTGCGTTT CTACAAACTC
5641 TTCCTGTCGT CATATCTACA AGCCGGCGCG CCATTCCGGG GATCCGTCGA CCTGCAGTTC
5701 GAAGTTCCTA TTCTCTAGAA AGTATAGGAA CTTCAGAGCG CTTTTGAAGC TCACGCTGCC
5761 GCAAGCACTC AGGGCGCAAG GGCTGCTAAA GGAAGCGGAA CACGTAGAAA GCCAGTCCGC
5821 AGAAACGGTG CTGACCCCGG ATGAATGTCA GCTACTGGGC TATCTGGACA AGGGAAAACG
5881 CAAGCGCAAA GAGAAAGCAG GTAGCTTGCA GTGGGCTTAC ATGGCGATAG CTAGACTGGG
5941 CGGTTTTATG GACAGCAAGC GAACCGGAAT TGCCAGCTGG GGCGCCCTCT GGTAAGGTTG
6001 GGAAGCCCTG CAAAGTAAAC TGGATGGCTT TCTTGCCGCC AAGGATCTGA TGGCGCAGGG
6061 GATCAAGATC TGATCAAGAG ACAGGATGAG GATCGTTTCG CATGATTGAA CAAGATGGAT
6121 TGCACGCAGG TTCTCCGGCC GCTTGGGTGG AGAGGCTATT CGGCTATGAC TGGGCACAAC
6181 AGACAATCGG CTGCTCTGAT GCCGCCGTGT TCCGGCTGTC AGCGCAGGGG CGCCCGGTTC
6241 TTTTTGTCAA GACCGACCTG TCCGGTGCCC TGAATGAACT GCAGGACGAG GCAGCGCGGC
6301 TATCGTGGCT GGCCACGACG GGCGTTCCTT GCGCAGCTGT GCTCGACGTT GTCACTGAAG
6361 CGGGAAGGGA CTGGCTGCTA TTGGGCGAAG TGCCGGGGCA GGATCTCCTG TCATCTCACC
6421 TTGCTCCTGC CGAGAAAGTA TCCATCATGG CTGATGCAAT GCGGCGGCTG CATACGCTTG
6481 ATCCGGCTAC CTGCCCATTC GACCACCAAG CGAAACATCG CATCGAGCGA GCACGTACTC
6541 GGATGGAAGC CGGTCTTGTC GATCAGGATG ATCTGGACGA AGAGCATCAG GGGCTCGCGC
6601 CAGCCGAACT GTTCGCCAGG CTCAAGGCGC GCATGCCCGA CGGCGAGGAT CTCGTCGTGA
6661 CCCATGGCGA TGCCTGCTTG CCGAATATCA TGGTGGAAAA TGGCCGCTTT TCTGGATTCA
6721 TCGACTGTGG CCGGCTGGGT GTGGCGGACC GCTATCAGGA CATAGCGTTG GCTACCCGTG
6781 ATATTGCTGA AGAGCTTGGC GGCGAATGGG CTGACCGCTT CCTCGTGCTT TACGGTATCG
6841 CCGCTCCCGA TTCGCAGCGC ATCGCCTTCT ATCGCCTTCT TGACGAGTTC TTCTAATAAG
6901 GGGATCTTGA AGTTCCTATT CCGAAGTTCC TATTCTCTAG AAAGTATAGG AACTTCGAAG
6961 CAGCTCCAGC CTACAATCGA TGCGGGATAA TCCGAAGTGG TCAGACTGGA AAATCAGAGG
7021 GCAGGAACTG CTGAACAGCA AAAAGTCAGA TAGCACCACA TAGCAGACCC GCCATAAAAC
7081 GCCCTGAGAA GCCCGTGACG GGCTTTTCTT GTATTATGGG TAGTTTCCTT GCATGAATCC
7141 ATAAAAGGCG CCTGTAGTGC CATTTACCCC CATTCACTGC CAGAGCCGTG AGCGCAGCGA
7201 ACTGAATGTC ACGAAAAAGA CAGCGACTCA GGTGCCTGAT GGTCGGAGAC AAAAGGAATA
7261 TTCAGCGATT TGCCCGAGCT TGCGAGGGTG CTACTTAAGC CTTTAGGGTT TTAAGGTCTG
7321 TTTTGTAGAG GAGCAAACAG CGTTTGCGAC ATCCTTTTGT AATACTGCGG AACTGACTAA
7381 AGTAGTGAGT TATACACAGG GCTGGGATCT ATTCTTTTTA TCTTTTTTTA TTCTTTCTTT
7441 ATTCTATAAA TTATAACCAC TTGAATATAA ACAAAAAAAA CACACAAAGG TCTAGCGGAA
7501 TTTACAGAGG GTCTAGCAGA ATTTACAAGT TTTCCAGCAA AGGTCTAGCA GAATTTACAG
7561 ATACCCACAA CTCAAAGGAA AAGGACTAGT AATTATCATT GACTAGCCCA TCTCAATTGG
7621 TATAGTGATT AAAATCACCT AGACCAATTG AGATGTATGT CTGAATTAGT TGTTTTCAAA
7681 GCAAATGAAC TAGCGATTAG TCGCTATGAC TTAACGGAGC ATGAAACCAA GCTAATTTTA
7741 TGCTGTGTGG CACTACTCAA CCCCACGATT GAAAACCCTA CAAGGAAAGA ACGGACGGTA
7801 TCGTTCACTT ATAACCAATA CGCTCAGATG ATGAACATCA GTAGGGAAAA TGCTTATGGT
7861 GTATTAGCTA AAGCAACCAG AGAGCTGATG ACGAGAACTG TGGAAATCAG GAATCCTTTG
7921 GTTAAAGGCT TTGAGATTTT CCAGTGGACA AACTATGCCA AGTTCTCAAG CGAAAAATTA
7981 GAATTAGTTT TTAGTGAAGA GATATTGCCT TATCTTTTCC AGTTAAAAAA ATTCATAAAA
8041 TATAATCTGG AACATGTTAA GTCTTTTGAA AACAAATACT CTATGAGGAT TTATGAGTGG
8101 TTATTAAAAG AACTAACACA AAAGAAAACT CACAAGGCAA ATATAGAGAT TAGCCTTGAT
8161 GAATTTAAGT TCATGTTAAT GCTTGAAAAT AACTACCATG AGTTTAAAAG GCTTAACCAA
8221 TGGGTTTTGA AACCAATAAG TAAAGATTTA AACACTTACA GCAATATGAA ATTGGTGGTT
8281 GATAAGCGAG GCCGCCCGAC TGATACGTTG ATTTTCCAAG TTGAACTAGA TAGACAAATG
```

```
8341 GATCTCGTAA CCGAACTTGA GAACAACCAG ATAAAAATGA ATGGTGACAA AATACCAACA
8401 ACCATTACAT CAGATTCCTA CCTACGTAAC GGACTAAGAA AAACACTACA CGATGCTTTA
8461 ACTGCAAAAA TTCAGCTCAC CAGTTTTGAG GCAAAATTTT TGAGTGACAT GCAAAGTAAG
8521 CATGATCTCA ATGGTTCGTT CTCATGGCTC ACGCAAAAAC AACGAACCAC ACTAGAGAAC
8581 ATACTGGCTA AATACGGAAG GATCTGAGGT TCTTATGGCT CTTGTATCTA TCAGTGAAGC
8641 ATCAAGACTA ACAAACAAAA GTAGAACAAC TGTTCACCGT TAGATATCAA AGGGAAAACT
8701 GTCCATATGC ACAGATGAAA ACGGTGTAAA AAAGATAGAT ACATCAGAGC TTTTACGAGT
8761 TTTTGGTGCA TTTAAAGCTG TTCACCATGA ACAGATCGAC AATGTAACAG ATGAACAGCA
8821 TGTAACACCT AATAGAACAG GTGAAACCAG TAAAACAAAG CAACTAGAAC ATGAAATTGA
8881 ACACCTGAGA CAACTTGTTA CAGCTCAACA GTCACACATA GACAGCCTGA AACAGGCGAT
8941 GCTGCTTATC GAATCAAAGC TGCCGACAAC ACGGGAGCCA GTGACGCCTC CCGTGGGGAA
9001 AAAATCATGG CAATTCTGGA AGAAATAGC
//
```

poGFP2

```
LOCUS       Exported              6771 bp ds-DNA     circular SYN 17-JUN-2019
DEFINITION  synthetic circular DNA.
ACCESSION   .
VERSION     .
KEYWORDS    .
SOURCE      synthetic DNA construct
  ORGANISM  synthetic DNA construct
REFERENCE   1  (bases 1 to 6771)
AUTHORS   Alexander S. Mankin
TITLE     Direct Submission
  JOURNAL   Exported Monday, Jun 17, 2019 from SnapGene 4.3.10
            https://www.snapgene.com
FEATURES             Location/Qualifiers
     source          1..6771
                     /organism="synthetic DNA construct"
                     /mol_type="other DNA"
                     /note="color: #ffffff"
     promoter        21..74
                     /locus_tag="pTet ON promoter"
                     /label=pTet ON promoter
                     /note="color: #ffffff"
     protein_bind    21..39
                     /gene="tetO"
                     /label=tet operator
                     /bound_moiety="tetracycline repressor TetR"
                     /note="bacterial operator O2 for the tetR and tetA genes"
                     /note="color: #31849b"
     protein_bind    46..64
                     /gene="tetO"
                     /label=tet operator
                     /bound_moiety="tetracycline repressor TetR"
                     /note="bacterial operator O2 for the tetR and tetA genes"
                     /note="color: #31849b"
     RBS             83..94
                     /note="strong bacterial ribosome binding site (Elowitz and
                     Leibler, 2000)"
                     /note="color: #a6acb3"
     CDS             101..853
                     /locus_tag="LuxR"
                     /label=LuxR
                     /note="color: #993366; direction: RIGHT"
     terminator      898..969
                     /label=rrnB T1 terminator
                     /note="transcription terminator T1 from the E. coli rrnB
                     gene"
                     /note="color: #ffffff"
     terminator      985..1012
                     /label=T7Te terminator
                     /note="phage T7 early transcription terminator"
                     /note="color: #ffffff"
     promoter        1027..1081
                     /locus_tag="pLux"
                     /label=pLux
```

```
                     /note="color: #ffffff"
     RBS             1101..1107
                     /locus_tag="oRBS"
                     /label=oRBS
                     /note="color: #a6acb3"
     misc_feature    1114..1836
                     /locus_tag="sfGFP"
                     /label=sfGFP
                     /note="color: #a6acb3"
     terminator      2076..2162
                     /gene="Escherichia coli rrnB"
                     /label=rrnB T1 terminator
                     /note="transcription terminator T1 from the E. coli rrnB
                     gene"
                     /note="color: #ffffff"
     terminator      2254..2281
                     /label=rrnB T2 terminator
                     /note="transcription terminator T2 from the E. coli rrnB
                     gene"
                     /note="color: #ffffff"
     rep_origin      2867..3412
                     /direction=RIGHT
                     /label=p15A ori
                     /note="Plasmids containing the medium-copy-number p15A
                     origin of replication can be propagated in E. coli cells
                     that contain a second plasmid with the ColE1 origin."
                     /note="color: #ffff00"
promoter        4080..4133
                     /locus_tag="pTet ON promoter"
                     /label=pTet ON promoter
                     /note="color: #ffffff"
     protein_bind    4099..4103
                     /gene="tetO"
                     /label=tet operator
                     /bound_moiety="tetracycline repressor TetR"
                     /note="bacterial operator O2 for the tetR and tetA genes"
                     /note="color: #31849b"
     protein_bind    4124..4133
                     /gene="tetO"
                     /label=tet operator
                     /bound_moiety="tetracycline repressor TetR"
                     /note="bacterial operator O2 for the tetR and tetA genes"
                     /note="color: #31849b"
     RBS             4142..4153
                     /note="strong bacterial ribosome binding site (Elowitz and
                     Leibler, 2000)"
                     /note="color: #a6acb3"
     CDS             4160..4912
                     /locus_tag="LuxR"
                     /label=LuxR
                     /note="color: #993366; direction: RIGHT"
     terminator      4957..5027
                     /label=rrnB T1 terminator
                     /note="transcription terminator T1 from the E. coli rrnB
                     gene"
                     /note="color: #ffffff"
```

```
     terminator      5044..5071
                     /label=T7Te terminator
                     /note="phage T7 early transcription terminator"
                     /note="color: #ffffff"
     promoter        5086..5140
                     /locus_tag="pLux"
                     /label=pLux
                     /note="color: #ffffff"
     CDS             5762..6553
                     /codon_start=1
                     /gene="aadA"
                     /product="aminoglycoside adenylyltransferase (Murphy,
                     1985)"
                     /label=SmR
                     /note="confers resistance to spectinomycin and
                     streptomycin"
                     /note="color: #993366"
                     /translation="MREAVIAEVSTQLSEVVGVIERHLEPTLLAVHLYGSAVDGGLKPH
                     SDIDLLVTVTVRLDETTRRALINDLLETSASPGESEILRAVEVTIVVHDDIIPWRYPAK
                     RELQFGEWQRNDILAGIFEPATIDIDLAILLTKAREHSVALVGPAAEELFDPVPEQDLF
                     EALNETLTLWNSPPDWAGDERNVVLTLSRIWYSAVTGKIAPKDVAADWAMERLPAQYQP
                     VILEARQAYLGQEEDRLASRADQLEEFVHYVKGEITKVVGK"
ORIGIN

        1 ATTCGCGGCC GCTTCTAGAG TCCCTATCAG TGATAGAGAT TGACATCCCT ATCAGTGATA
       61 GAGATACTGA GCACTACTAG AGAAAGAGGA GAAATACTAG ATGAAAAACA TAAATGCCGA
      121 CGACACATAC AGAATAATTA ATAAAATTAA AGCTTGTAGA AGCAATAATG ATATTAATCA
      181 ATGCTTATCT GATATGACTA AAATGGTACA TTGTGAATAT TATTTACTCG CGATCATTTA
      241 TCCTCATTCT ATGGTTAAAT CTGATATTTC AATCCTAGAT AATTACCCTA AAAAATGGAG
      301 GCAATATTAT GATGACGCTA ATTTAATAAA ATATGATCCT ATAGTAGATT ATTCTAACTC
      361 CAATCATTCA CCAATTAATT GGAATATATT TGAAAACAAT GCTGTAAATA AAAAATCTCC
      421 AAATGTAATT AAAGAAGCGA AAACATCAGG TCTTATCACT GGGTTTAGTT TCCCTATTCA
      481 TACGGCTAAC AATGGCTTCG GAATGCTTAG TTTTGCACAT TCAGAAAAAG ACAACTATAT
      541 AGATAGTTTA TTTTTACATG CGTGTATGAA CATACCATTA ATTGTTCCTT CTCTAGTTGA
      601 TAATTATCGA AAAATAAATA TAGCAAATAA TAAATCAAAC AACGATTTAA CCAAAAGAGA
      661 AAAAGAATGT TTAGCGTGGG CATGCGAAGG AAAAAGCTCT TGGGATATTT CAAAAATATT
      721 AGGTTGCAGT GAGCGTACTG TCACTTTCCA TTTAACCAAT GCGCAAATGA AACTCAATAC
      781 AACAAACCGC TGCCAAAGTA TTTCTAAAGC AATTTTAACA GGAGCAATTG ATTGCCCATA
      841 CTTTAAAAAT TAATAACACT GATAGTGCTA GTGTAGATCA CTACTAGAGC CAGGCATCAA
      901 ATAAAACGAA AGGCTCAGTC GAAAGACTGG GCCTTTCGTT TTATCTGTTG TTTGTCGGTG
      961 AACGCTCTCT ACTAGAGTCA CACTGGCTCA CCTTCGGGTG GGCCTTTCTG CGTTTATATA
     1021 CTAGAGACCT GTAGGATCGT ACAGGTTTAC GCAAGAAAAT GGTTTGTTAT AGTCGAATAA
     1081 ACTATATCTG TTATTTTTTC CAACCACAGA TCTATGAGCA AAGGTGAAGA ACTGTTTACC
     1141 GGCGTTGTGC CGATTCTGGT GGAACTGGAT GGCGATGTGA ACGGTCACAA ATTCAGCGTG
     1201 CGTGGTGAAG GTGAAGGCGA TGCCACGATT GGCAAACTGA CGCTGAAATT TATCTGCACC
     1261 ACCGGCAAAC TGCCGGTGCC GTGGCCGACG CTGGTGACCA CCCTGACCTA TGGCGTTCAG
     1321 TGTTTTAGTC GCTATCCGGA TCACATGAAA CGTCACGATT TCTTTAAATC TGCAATGCCG
     1381 GAAGGCTATG TGCAGGAACG TACGATTAGC TTTAAAGATG ATGGCAAATA TAAAACGCGC
     1441 GCCGTTGTGA AATTTGAAGG CGATACCCTG GTGAACCGCA TTGAACTGAA AGGCACGGAT
     1501 TTTAAAGAAG ATGGCAATAT CCTGGGCCAT AAACTGGAAT ACAACTTTAA TAGCCATAAT
     1561 GTTTATATTA CGGCGGATAA ACAGAAAAAT GGCATCAAAG CGAATTTTAC CGTTCGCCAT
     1621 AACGTTGAAG ATGGCAGTGT GCAGCTGGCA GATCATTATC AGCAGAATAC CCCGATTGGT
     1681 GATGGTCCGG TGCTGCTGCC GGATAATCAT TATCTGAGCA CGCAGACCGT TCTGTCTAAA
     1741 GATCCGAACG AAAAAGGCAC GCGGGACCAC ATGGTTCTGC ACGAATATGT GAATGCGGCA
     1801 GGTATTACGT GGAGCCATCC GCAGTTCGAA AATAAGTCGG ACCGGCTGCT AACAAAGCCC
     1861 GCGGCCGCTG AAGATCGATC TCGACGAGTG AGAGAAGATT TTCAGCCTGA TACAGATTAA
     1921 ATCAGAACGC AGAAGCGGTC TGATAAAACA GAATTTGCCT GGCGGCAGTA GCGCGGTGGT
```

1981 CCCACCTGAC CCCATGCCGA ACTCAGAAGT GAAACGCCGT AGCGCCGATG GTAGTGTGGG
2041 GTCACCCCAT GCGAGAGTAG GGAACTGCCA GGCATCAAAT AAAACGAAAG GCTCAGTCGA
2101 AAGACTGGGC CTTTCGTTTT ATCTGTTGTT TGTCGGTGAA CGCTCTCCTG AGTAGGACAA
2161 ATCCGCCGGG AGCGGATTTG AACGTTGCGA AGCAACGGCC CGGAGGGTGG CGGGCAGGAC
2221 GCCCGCCATA AACTGCCAGG CATCAAATTA AGCAGAAGGC CATCCTGACG GATGGCCTTT
2281 TTGCGTTTCT ACACTCGAGG ATCTCTTTCC TTTCCATCAA AAAAATATTG ATGAAATGAG
2341 CTGTGGGGCG TTATTTAGGT TTTTTCTTCT TTCGAAAAAA TCTTTCTTTA TGAAGTTAAA
2401 AGCTATGTAT TCAATAGCAT ATTTTGAATA TGGACATAGA ATAGTGCTTA TCACTATTGC
2461 ATATAGCATC TTATCTGACA CAAGGAAATA ATACCCTTCG CTGTTTTTTG TTATAAGGTA
2521 TATATATATA AGTGTGCAGT ACAGGCCAAA TAAAATATTT TTTATGTAGT ATCTTAAATC
2581 CCGCAAGAGG CCCGGCAGTA CCGGCATAAC CAAGCCTATG CCTACAGCAT CCAGGGTGAC
2641 GGTGCCGAGG ATGACGATGA GCGCATTGTT AGATTTCATA CACGGTGCCT GACTGCGTTA
2701 GCAATTTAAC TGTGATAAAC TACCGCATTA AAGCTTATCG ATGATAAGCT GTCAAACATG
2761 AGAATTACAA CTTATATCGT ATGGGGCTGA CTTCAGGTGC TACATTTGAA GAGATAAATT
2821 GCACTGAAAT CTAGAAATAT TTTATCTGAT TAATAAGATG ATCTTCTTGA GATCGTTTTG
2881 GTCTGCGCGT AATCTCTTGC TCTGAAAACG AAAAAACCGC CTTGCAGGGC GGTTTTTCGA
2941 AGGTTCTCTG AGCTACCAAC TCTTTGAACC GAGGTAACTG GCTTGGAGGA GCGCAGTCAC
3001 CAAAACTTGT CCTTTCAGTT TAGCCTTAAC CGGCGCATGA CTTCAAGACT AACTCCTCTA
3061 AATCAATTAC CAGTGGCTGC TGCCAGTGGT GCTTTTGCAT GTCTTTCCGG GTTGGACTCA
3121 AGACGATAGT TACCGGATAA GGCGCAGCGG TCGGACTGAA CGGGGGGTTC GTGCATACAG
3181 TCCAGCTTGG AGCGAACTGC CTACCCGGAA CTGAGTGTCA GGCGTGGAAT GAGACAAACG
3241 CGGCCATAAC AGCGGAATGA CACCGGTAAA CCGAAAGGCA GGAACAGGAG AGCGCACGAG
3301 GGAGCCGCCA GGGGGAAACG CCTGGTATCT TTATAGTCCT GTCGGGTTTC GCCACCACTG
3361 ATTTGAGCGT CAGATTTCGT GATGCTTGTC AGGGGGGCGG AGCCTATGGA AAAACGGCTT
3421 TGCCGCGGCC CTCTCACTTC CCTGTTAAGT ATCTTCCTGG CATCTTCCAG GAAATCTCCG
3481 CCCCGTTCGT AAGCCATTTC CGCTCGCCGC AGTCGAACGA CCGAGCGTAG CGAGTCAGTG
3541 AGCGAGGAAG CGGAATATAT CCTGTATCAC ATATTCTGCT GACGCACCGG TGCAGCCTTT
3601 TTTCTCCTGC CACATGAAGC ACTTCACTGA CACCCTCATC AGTGCCAACA TAGTAAGCCA
3661 GTATACACTC CGCTAGCGCT GATGTCCGGC GGTGCTTTTG CCGTTACGCA CCACCCCGTC
3721 AGTAGCTGAA CAGGAGGGAC AGAGCTTTAT GCTTGTAAAC CGTTTTGTGA AAAAATTTTT
3781 AAAATAAAAA AGGGGACCTC TAGGGTCCCC AATTAATTAG TAATATAATC TATTAAAGGT
3841 CATTCAAAAG GTCATCCACC GGATCAATTC CCCTGCTCGC GCAGGCTGGG TGCCAAGCTC
3901 TCGGGTAACA TCAAGGCCCG ATCCTTGGAG CCCTTGCCCT CCCGCACGAT GATCGTGCCG
3961 TGATCGAAAA TCCAGATCCT TGACCCGCAT TTGCAAACCC TCACTGATCC GCATGGGGCC
4021 CCCGTTCCAT ACAGAAGCTG GGCGAAGTAA TCGCAACATA TTCGCGGCCG CTTCTAGAGT
4081 CCCTATCAGT GATAGAGATT GACATCCCTA TCAGTGATAG AGATACTGAG CACTACTAGA
4141 GAAAGAGGAG AAATACTAGA TGAAAAACAT AAATGCCGAC GACACATACA GAATAATTAA
4201 TAAAATTAAA GCTTGTAGAA GCAATAATGA TATTAATCAA TGCTTATCTG ATATGACTAA
4261 AATGGTACAT TGTGAATATT ATTTACTCGC GATCATTTAT CCTCATTCTA TGGTTAAATC
4321 TGATATTTCA ATCCTAGATA ATTACCCTAA AAAATGGAGG CAATATTATG ATGACGCTAA
4381 TTTAATAAAA TATGATCCTA TAGTAGATTA TTCTAACTCC AATCATTCAC CAATTAATTG
4441 GAATATATTT GAAAACAATG CTGTAAATAA AAAATCTCCA AATGTAATTA AAGAAGCGAA
4501 AACATCAGGT CTTATCACTG GGTTTAGTTT CCCTATTCAT ACGGCTAACA ATGGCTTCGG
4561 AATGCTTAGT TTTGCACATT CAGAAAAAGA CAACTATATA GATAGTTTAT TTTTACATGC
4621 GTGTATGAAC ATACCATTAA TTGTTCCTTC TCTAGTTGAT AATTATCGAA AAATAAATAT
4681 AGCCAATAAT AAATCAAACA ACGATTTAAC CAAAAGAGAA AAAGAATGTT TAGCGTGGGC
4741 ATGCGAAGGA AAAAGCTCTT GGGATATTTC AAAAATATTA GGTTGCAGTG AGCGTACTGT
4801 CACTTTCCAT TTAACCAATG CGCAAATGAA ACTCAATACA ACAAACCGCT GCCAAAGTAT
4861 TTCTAAAGCA ATTTTAACAG GAGCAATTGA TTGCCCATAC TTTAAAAATT AATAACACTG
4921 ATAGTGCTAG TGTAGATCAC TACTAGAGCC AGGCATCAAA TAAAACGAAA GGCTCAGTCG
4981 AAAGACTGGG CCTTTCGTTT TATCTGTTGT TTGTCGGTGA ACGCTCTCTA CTAGAGTCAC
5041 ACTGGCTCAC CTTCGGGTGG GCCTTTCTGC GTTTATATAC TAGAGACCTG TAGGATCGTA
5101 CAGGTTTACG CAAGAAAATG GTTTGTTATA GTCGAATAAA CTATATCTGT TATTTTTTCC
5161 AACCACTGAT CCGCATGGGG CCCCCGTTCC ATACAGAAGC TGGGCGAACA AACGATGCTC
5221 GCCTTCCAGA AAACCGAGGA TGCGAACCAC TTCATCCGGG GTCAGCACCA CCGGCAAGCG
5281 CCGCGACGGC CGAGGTCTTC CGATCTCCTG AAGCCAGGGC AGATCCGTGC ACAGCACCTT
5341 GCCGTAGAAG AACAGCAAGG CCGCCAATGC CTGACGATGC GTGGAGACCG AAACCTTGCG

```
     5401 CTCGTTCGCC AGCCAGGACA GAAATGCCTC GACTTCGCTG CTGCCCAAGG TTGCCGGGTG
     5461 ACGCACACCG TGGAAACGGA TGAAGGCACG AACCCAGTGG ACATAAGCCT GTTCGGTTCG
     5521 TAAGCTGTAA TGCAAGTAGC GTATGCGCTC ACGCAACTGG TCCAGAACCT TGACCGAACG
     5581 CAGCGGTGGT AACGGCGCAG TGGCGGTTTT CATGGCTTGT TATGACTGTT TTTTTGGGGT
     5641 ACAGTCTATG CCTCGGGCAT CCAAGCAGCA AGCGCGTTAC GCCGTGGGTC GATGTTTGAT
     5701 GTTATGGAGC AGCAACGATG TTACGCAGCA GGGCAGTCGC CCTAAAACAA AGTTAAACAT
     5761 CATGAGGGAA GCGGTGATCG CCGAAGTATC GACTCAACTA TCAGAGGTAG TTGGCGTCAT
     5821 CGAGCGCCAT CTCGAACCGA CGTTGCTGGC CGTACATTTG TACGGCTCCG CAGTGGATGG
     5881 CGGCCTGAAG CCACACAGTG ATATTGATTT GCTGGTTACG GTGACCGTAA GGCTTGATGA
     5941 AACAACGCGG CGAGCTTTGA TCAACGACCT TTTGGAAACT TCGGCTTCCC CTGGAGAGAG
     6001 CGAGATTCTC CGCGCTGTAG AAGTCACCAT TGTTGTGCAC GACGACATCA TTCCGTGGCG
     6061 TTATCCAGCT AAGCGCGAAC TGCAATTTGG AGAATGGCAG CGCAATGACA TTCTTGCAGG
     6121 TATCTTCGAG CCAGCCACGA TCGACATTGA TCTGGCTATC TTGCTGACAA AAGCAAGAGA
     6181 ACATAGCGTT GCCTTGGTAG GTCCAGCGGC GGAGGAACTC TTTGATCCGG TTCCTGAACA
     6241 GGATCTATTT GAGGCGCTAA ATGAAACCTT AACGCTATGG AACTCGCCGC CCGACTGGGC
     6301 TGGCGATGAG CGAAATGTAG TGCTTACGTT GTCCCGCATT TGGTACAGCG CAGTAACCGG
     6361 CAAAATCGCG CCGAAGGATG TCGCTGCCGA CTGGGCAATG GAGCGCCTGC CGGCCCAGTA
     6421 TCAGCCCGTC ATACTTGAAG CTAGACAGGC TTATCTTGGA CAAGAAGAAG ATCGCTTGGC
     6481 CTCGCGCGCA GATCAGTTGG AAGAATTTGT CCACTACGTG AAAGGCGAGA TCACCAAGGT
     6541 AGTCGGCAAA TAATGTCTAA CAATTCGTTC AAGCCGACGC CGCTTCGCGG CGCGGCTTAA
     6601 CTCAAGCGTT AGATGCACTA AGCACATAAT TGCTCACAGC CAAACTATCA GGTCAAGTCT
     6661 GCTTTTATTA TTTTTAAGCG TGCATAATAA GCCCTACACA AATTGGGAGA TATATCATGA
     6721 AAGGCTGGCT TTTTCTTGTT ATCGCAATAG TTGGCGAAGT AATCGCAACA T
//
```

poLuc2

```
LOCUS       Exported                7701 bp ds-DNA     circular SYN 17-JUN-2019
DEFINITION  synthetic circular DNA.
ACCESSION   .
VERSION     .
KEYWORDS    .
SOURCE      synthetic DNA construct
  ORGANISM  synthetic DNA construct
REFERENCE   1  (bases 1 to 7701)
AUTHORS   Alexander S. Mankin
TITLE     Direct Submission
  JOURNAL   Exported Monday, Jun 17, 2019 from SnapGene 4.3.10
            https://www.snapgene.com
FEATURES             Location/Qualifiers
     source          1..7701
                     /organism="synthetic DNA construct"
                     /mol_type="other DNA"
                     /note="color: #ffffff"
     promoter        21..74
                     /locus_tag="pTet ON promoter"
                     /label=pTet ON promoter
                     /note="color: #ffffff"
     protein_bind    21..39
                     /gene="tetO"
                     /label=tet operator
                     /bound_moiety="tetracycline repressor TetR"
                     /note="bacterial operator O2 for the tetR and tetA genes"
                     /note="color: #31849b"
     protein_bind    46..64
                     /gene="tetO"
                     /label=tet operator
                     /bound_moiety="tetracycline repressor TetR"
                     /note="bacterial operator O2 for the tetR and tetA genes"
                     /note="color: #31849b"
     RBS             83..94
                     /note="strong bacterial ribosome binding site (Elowitz and
                     Leibler, 2000)"
                     /note="color: #a6acb3"
     CDS             101..853
                     /locus_tag="LuxR"
                     /label=LuxR
                     /note="color: #993366; direction: RIGHT"
     terminator      898..969
                     /label=rrnB T1 terminator
                     /note="transcription terminator T1 from the E. coli rrnB
                     gene"
                     /note="color: #ffffff"
     terminator      985..1012
                     /label=T7Te terminator
                     /note="phage T7 early transcription terminator"
                     /note="color: #ffffff"
     promoter        1027..1081
                     /locus_tag="pLux"
                     /label=pLux
```

```
                /note="color: #ffffff"
     RBS            1101..1107
                /locus_tag="oRBS"
                /label=oRBS
                /note="color: #a6acb3"
     misc_feature   1114..2766
                /locus_tag="Firefly Luciferase"
                /label= Firefly Luciferase
                /note="color: #a6acb3"
     terminator     3006..3092
                /gene="Escherichia coli rrnB"
                /label=rrnB T1 terminator
                /note="transcription terminator T1 from the E. coli rrnB
                gene"
                /note="color: #ffffff"
     terminator     3184..3211
                /label=rrnB T2 terminator
                /note="transcription terminator T2 from the E. coli rrnB
                gene"
                /note="color: #ffffff"
     rep_origin     3797..4342
                /direction=RIGHT
                /label=p15A ori
                /note="Plasmids containing the medium-copy-number p15A
                origin of replication can be propagated in E. coli cells
                that contain a second plasmid with the ColE1 origin."
                /note="color: #ffff00"
promoter        5010..5063
                /locus_tag="pTet ON promoter"
                /label=pTet ON promoter
                /note="color: #ffffff"
     protein_bind   5029..5033
                /gene="tetO"
                /label=tet operator
                /bound_moiety="tetracycline repressor TetR"
                /note="bacterial operator O2 for the tetR and tetA genes"
                /note="color: #31849b"
     protein_bind   5054..5063
                /gene="tetO"
                /label=tet operator
                /bound_moiety="tetracycline repressor TetR"
                /note="bacterial operator O2 for the tetR and tetA genes"
                /note="color: #31849b"
     RBS            5072..5083
                /note="strong bacterial ribosome binding site (Elowitz and
                Leibler, 2000)"
                /note="color: #a6acb3"
     CDS            5090..5842
                /locus_tag="LuxR"
                /label=LuxR
                /note="color: #993366; direction: RIGHT"
     terminator     5887..5957
                /label=rrnB T1 terminator
                /note="transcription terminator T1 from the E. coli rrnB
                gene"
                /note="color: #ffffff"
```

```
     terminator      5974..6001
                     /label=T7Te terminator
                     /note="phage T7 early transcription terminator"
                     /note="color: #ffffff"
     promoter        6016..6070
                     /locus_tag="pLux"
                     /label=pLux
                     /note="color: #ffffff"
     CDS             6692..7483
                     /codon_start=1
                     /gene="aadA"
                     /product="aminoglycoside adenylyltransferase (Murphy,
                     1985)"
                     /label=SmR
                     /note="confers resistance to spectinomycin and
                     streptomycin"
                     /note="color: #993366"
                     /translation="MREAVIAEVSTQLSEVVGVIERHLEPTLLAVHLYGSAVDGGLKPH
                     SDIDLLVTVTVRLDETTRRALINDLLETSASPGESEILRAVEVTIVVHDDIIPWRYPAK
                     RELQFGEWQRNDILAGIFEPATIDIDLAILLTKAREHSVALVGPAAEELFDPVPEQDLF
                     EALNETLTLWNSPPDWAGDERNVVLTLSRIWYSAVTGKIAPKDVAADWAMERLPAQYQP
                     VILEARQAYLGQEEDRLASRADQLEEFVHYVKGEITKVVGK"
ORIGIN
        1 ATTCGCGGCC GCTTCTAGAG TCCCTATCAG TGATAGAGAT TGACATCCCT ATCAGTGATA
       61 GAGATACTGA GCACTACTAG AGAAAGAGGA GAAATACTAG ATGAAAAACA TAAATGCCGA
      121 CGACACATAC AGAATAATTA ATAAAATTAA AGCTTGTAGA AGCAATAATG ATATTAATCA
      181 ATGCTTATCT GATATGACTA AAATGGTACA TTGTGAATAT TATTTACTCG CGATCATTTA
      241 TCCTCATTCT ATGGTTAAAT CTGATATTTC AATCCTAGAT AATTACCCTA AAAAATGGAG
      301 GCAATATTAT GATGACGCTA ATTTAATAAA ATATGATCCT ATAGTAGATT ATTCTAACTC
      361 CAATCATTCA CCAATTAATT GGAATATATT TGAAAACAAT GCTGTAAATA AAAAATCTCC
      421 AAATGTAATT AAAGAAGCGA AAACATCAGG TCTTATCACT GGGTTTAGTT TCCCTATTCA
      481 TACGGCTAAC AATGGCTTCG GAATGCTTAG TTTTGCACAT TCAGAAAAAG ACAACTATAT
      541 AGATAGTTTA TTTTTACATG CGTGTATGAA CATACCATTA ATTGTTCCTT CTCTAGTTGA
      601 TAATTATCGA AAAATAAATA TAGCAAATAA TAAATCAAAC AACGATTTAA CCAAAAGAGA
      661 AAAAGAATGT TTAGCGTGGG CATGCGAAGG AAAAAGCTCT TGGGATATTT CAAAAATATT
      721 AGGTTGCAGT GAGCGTACTG TCACTTTCCA TTTAACCAAT GCGCAAATGA AACTCAATAC
      781 AACAAACCGC TGCCAAAGTA TTTCTAAAGC AATTTTAACA GGAGCAATTG ATTGCCCATA
      841 CTTTAAAAAT TAATAACACT GATAGTGCTA GTGTAGATCA CTACTAGAGC CAGGCATCAA
      901 ATAAAACGAA AGGCTCAGTC GAAAGACTGG GCCTTTCGTT TTATCTGTTG TTTGTCGGTG
      961 AACGCTCTCT ACTAGAGTCA CACTGGCTCA CCTTCGGGTG GGCCTTTCTG CGTTTATATA
     1021 CTAGAGACCT GTAGGATCGT ACAGGTTTAC GCAAGAAAAT GGTTTGTTAT AGTCGAATAA
     1081 ACTATATCTG TTATTTTTTC CAACCACAGA TCTATGGAAG ACGCCAAAAA CATAAAGAAA
     1141 GGCCCGGCGC CATTCTATCC GCTAGAGGAT GGAACCGCTG GAGAGCAACT GCATAAGGCT
     1201 ATGAAGAGAT ACGCCCTGGT TCCTGGAACA ATTGCTTTTA CAGATGCACA TATCGAGGTG
     1261 AACATCACGT ACGCGGAATA CTTCGAAATG TCCGTTCGGT TGGCAGAAGC TATGAAACGA
     1321 TATGGGCTGA ATACAAATCA CAGAATCGTC GTATGCAGTG AAAACTCTCT TCAATTCTTT
     1381 ATGCCGGTGT TGGGCGCGTT ATTTATCGGA GTTGCAGTTG CGCCCGCGAA CGACATTTAT
     1441 AATGAACGTG AATTGCTCAA CAGTATGAAC ATTTCGCAGC CTACCGTAGT GTTTGTTTCC
     1501 AAAAAGGGGT TGCAAAAAAT TTTGAACGTG CAAAAAAAAT TACCAATAAT CCAGAAAATT
     1561 ATTATCATGG ATTCTAAAAC GGATTACCAG GGATTTCAGT CGATGTACAC GTTCGTCACA
     1621 TCTCATCTAC CTCCCGGTTT TAATGAATAC GATTTTGTAC CAGAGTCCTT TGATCGTGAC
     1681 AAAACAATTG CACTGATAAT GAACTCCTCT GGATCTACTG GGTTACCTAA GGGTGTGGCC
     1741 CTTCCGCATA GAACTGCCTG CGTCAGATTC TCGCATGCCA GAGATCCTAT TTTTGGCAAT
     1801 CAAATCATTC CGGATACTGC GATTTTAAGT GTTGTTCCAT TCCATCACGG TTTTGGAATG
     1861 TTTACTACAC TCGGATATTT GATATGTGGA TTTCGAGTCG TCTTAATGTA TAGATTTGAA
     1921 GAAGAGCTGT TTTTACGATC CCTTCAGGAT TACAAAATTC AAAGTGCGTT GCTAGTACCA
```

```
1981 ACCCTATTTT CATTCTTCGC CAAAAGCACT CTGATTGACA AATACGATTT ATCTAATTTA
2041 CACGAAATTG CTTCTGGGGG CGCACCTCTT TCGAAAGAAG TCGGGGAAGC GGTTGCAAAA
2101 CGCTTCCATC TTCCAGGGAT ACGACAAGGA TATGGGCTCA CTGAGACTAC ATCAGCTATT
2161 CTGATTACAC CCGAGGGGGA TGATAAACCG GGCGCGGTCG GTAAAGTTGT TCCATTTTTT
2221 GAAGCGAAGG TTGTGGATCT GGATACCGGG AAAACGCTGG GCGTTAATCA GAGAGGCGAA
2281 TTATGTGTCA GAGGACCTAT GATTATGTCC GGTTATGTAA ACAATCCGGA AGCGACCAAC
2341 GCCTTGATTG ACAAGGATGG ATGGCTACAT TCTGGAGACA TAGCTTACTG GGACGAAGAC
2401 GAACACTTCT TCATAGTTGA CCGCTTGAAG TCTTTAATTA AATACAAAGG ATACCAGGTG
2461 GCCCCCGCTG AATTGGAGTC GATATTGTTA CAACACCCCA ACATCTTCGA CGCGGGCGTG
2521 GCAGGTCTTC CCGACGATGA CGCCGGTGAA CTTCCCGCCG CCGTTGTTGT TTTGGAGCAC
2581 GGAAAGACGA TGACGGAAAA AGAGATCGTG GATTACGTCG CCAGTCAAGT AACAACCGCC
2641 AAAAAGTTGC GCGGAGGAGT TGTGTTTGTG GACGAAGTAC CGAAAGGTCT TACCGGAAAA
2701 CTCGACGCAA GAAAAATCAG AGAGATCCTC ATAAAGGCCA AGAAGGGCGG AAAGTCCAAA
2761 TTGTAAGTCG ACCGGCTGCT AACAAAGCCC GCGGCCGCTG AAGATCGATC TCGACGAGTG
2821 AGAGAAGATT TTCAGCCTGA TACAGATTAA ATCAGAACGC AGAAGCGGTC TGATAAAACA
2881 GAATTTGCCT GGCGGCAGTA GCGCGGTGGT CCCACCTGAC CCCATGCCGA ACTCAGAAGT
2941 GAAACGCCGT AGCGCCGATG GTAGTGTGGG GTCACCCCAT GCGAGAGTAG GGAACTGCCA
3001 GGCATCAAAT AAAACGAAAG GCTCAGTCGA AAGACTGGGC CTTTCGTTTT ATCTGTTGTT
3061 TGTCGGTGAA CGCTCTCCTG AGTAGGACAA ATCCGCCGGG AGCGGATTTG AACGTTGCGA
3121 AGCAACGGCC CGGAGGGTGG CGGGCAGGAC GCCCGCCATA AACTGCCAGG CATCAAATTA
3181 AGCAGAAGGC CATCCTGACG GATGGCCTTT TTGCGTTTCT ACACTCGAGG ATCTCTTTCC
3241 TTTCCATCAA AAAAATATTG ATGAAATGAG CTGTGGGGCG TTATTTAGGT TTTTTCTTCT
3301 TTCGAAAAAA TCTTTCTTTA TGAAGTTAAA AGCTATGTAT TCAATAGCAT ATTTTGAATA
3361 TGGACATAGA ATAGTGCTTA TCACTATTGC ATATAGCATC TTATCTGACA CAAGGAAATA
3421 ATACCCTTCG CTGTTTTTTG TTATAAGGTA TATATATATA AGTGTGCAGT ACAGGCCAAA
3481 TAAAATATTT TTTATGTAGT ATCTTAAATC CCGCAAGAGG CCCGGCAGTA CCGGCATAAC
3541 CAAGCCTATG CCTACAGCAT CCAGGGTGAC GGTGCCGAGG ATGACGATGA GCGCATTGTT
3601 AGATTTCATA CACGGTGCCT GACTGCGTTA GCAATTTAAC TGTGATAAAC TACCGCATTA
3661 AAGCTTATCG ATGATAAGCT GTCAAACATG AGAATTACAA CTTATATCGT ATGGGGCTGA
3721 CTTCAGGTGC TACATTTGAA GAGATAAATT GCACTGAAAT CTAGAAATAT TTTATCTGAT
3781 TAATAAGATG ATCTTCTTGA GATCGTTTTG GTCTGCGCGT AATCTCTTGC TCTGAAAACG
3841 AAAAAACCGC CTTGCAGGGC GGTTTTTCGA AGGTTCTCTG AGCTACCAAC TCTTTGAACC
3901 GAGGTAACTG GCTTGGAGGA GCGCAGTCAC CAAAACTTGT CCTTTCAGTT TAGCCTTAAC
3961 CGGCGCATGA CTTCAAGACT AACTCCTCTA AATCAATTAC CAGTGGCTGC TGCCAGTGGT
4021 GCTTTTGCAT GTCTTTCCGG GTTGGACTCA AGACGATAGT TACCGGATAA GGCGCAGCGG
4081 TCGGACTGAA CGGGGGGTTC GTGCATACAG TCCAGCTTGG AGCGAACTGC CTACCCGGAA
4141 CTGAGTGTCA GGCGTGGAAT GAGACAAACG CGGCCATAAC AGCGGAATGA CACCGGTAAA
4201 CCGAAAGGCA GGAACAGGAG AGCGCACGAG GGAGCCGCCA GGGGGAAACG CCTGGTATCT
4261 TTATAGTCCT GTCGGGTTTC GCCACCACTG ATTTGAGCGT CAGATTTCGT GATGCTTGTC
4321 AGGGGGGCGG AGCCTATGGA AAAACGGCTT TGCCGCGGCC CTCTCACTTC CCTGTTAAGT
4381 ATCTTCCTGG CATCTTCCAG GAAATCTCCG CCCCGTTCGT AAGCCATTTC CGCTCGCCGC
4441 AGTCGAACGA CCGAGCGTAG CGAGTCAGTG AGCGAGGAAG CGGAATATAT CCTGTATCAC
4501 ATATTCTGCT GACGCACCGG TGCAGCCTTT TTTCTCCTGC CACATGAAGC ACTTCACTGA
4561 CACCCTCATC AGTGCCAACA TAGTAAGCCA GTATACACTC CGCTAGCGCT GATGTCCGGC
4621 GGTGCTTTTG CCGTTACGCA CCACCCCGTC AGTAGCTGAA CAGGAGGGAC AGAGCTTTAT
4681 GCTTGTAAAC CGTTTTGTGA AAAAATTTTT AAAATAAAAA AGGGGACCTC TAGGGTCCCC
4741 AATTAATTAG TAATATAATC TATTAAAGGT CATTCAAAAG GTCATCCACC GGATCAATTC
4801 CCCTGCTCGC GCAGGCTGGG TGCCAAGCTC TCGGGTAACA TCAAGGCCCG ATCCTTGGAG
4861 CCCTTGCCCT CCCGCACGAT GATCGTGCCG TGATCGAAAA TCCAGATCCT TGACCCGCAT
4921 TTGCAAACCC TCACTGATCC GCATGGGGCC CCCGTTCCAT ACAGAAGCTG GGCGAAGTAA
4981 TCGCAACATA TTCGCGGCCG CTTCTAGAGT CCCTATCAGT GATAGAGATT GACATCCCTA
5041 TCAGTGATAG AGATACTGAG CACTACTAGA GAAAGAGGAG AAATACTAGA TGAAAAACAT
5101 AAATGCCGAC GACACATACA GAATAATTAA TAAAATTAAA GCTTGTAGAA GCAATAATGA
5161 TATTAATCAA TGCTTATCTG ATATGACTAA AATGGTACAT TGTGAATATT ATTTACTCGC
5221 GATCATTTAT CCTCATTCTA TGGTTAAATC TGATATTTCA ATCCTAGATA ATTACCCTAA
5281 AAAATGGAGG CAATATTATG ATGACGCTAA TTTAATAAAA TATGATCCTA TAGTAGATTA
5341 TTCTAACTCC AATCATTCAC CAATTAATTG GAATATATTT GAAAACAATG CTGTAAATAA
```

```
5401 AAAATCTCCA AATGTAATTA AAGAAGCGAA AACATCAGGT CTTATCACTG GGTTTAGTTT
5461 CCCTATTCAT ACGGCTAACA ATGGCTTCGG AATGCTTAGT TTTGCACATT CAGAAAAAGA
5521 CAACTATATA GATAGTTTAT TTTTACATGC GTGTATGAAC ATACCATTAA TTGTTCCTTC
5581 TCTAGTTGAT AATTATCGAA AAATAAATAT AGCCAATAAT AAATCAAACA ACGATTTAAC
5641 CAAAAGAGAA AAAGAATGTT TAGCGTGGGC ATGCGAAGGA AAAAGCTCTT GGGATATTTC
5701 AAAAATATTA GGTTGCAGTG AGCGTACTGT CACTTTCCAT TTAACCAATG CGCAAATGAA
5761 ACTCAATACA ACAAACCGCT GCCAAAGTAT TTCTAAAGCA ATTTTAACAG GAGCAATTGA
5821 TTGCCCATAC TTTAAAAATT AATAACACTG ATAGTGCTAG TGTAGATCAC TACTAGAGCC
5881 AGGCATCAAA TAAAACGAAA GGCTCAGTCG AAAGACTGGG CCTTTCGTTT TATCTGTTGT
5941 TTGTCGGTGA ACGCTCTCTA CTAGAGTCAC ACTGGCTCAC CTTCGGGTGG GCCTTTCTGC
6001 GTTTATATAC TAGAGACCTG TAGGATCGTA CAGGTTTACG CAAGAAAATG GTTTGTTATA
6061 GTCGAATAAA CTATATCTGT TATTTTTTCC AACCACTGAT CCGCATGGGG CCCCCGTTCC
6121 ATACAGAAGC TGGGCGAACA AACGATGCTC GCCTTCCAGA AAACCGAGGA TGCGAACCAC
6181 TTCATCCGGG GTCAGCACCA CCGGCAAGCG CCGCGACGGC CGAGGTCTTC CGATCTCCTG
6241 AAGCCAGGGC AGATCCGTGC ACAGCACCTT GCCGTAGAAG AACAGCAAGG CCGCCAATGC
6301 CTGACGATGC GTGGAGACCG AAACCTTGCG CTCGTTCGCC AGCCAGGACA GAAATGCCTC
6361 GACTTCGCTG CTGCCCAAGG TTGCCGGGTG ACGCACACCG TGGAAACGGA TGAAGGCACG
6421 AACCCAGTGG ACATAAGCCT GTTCGGTTCG TAAGCTGTAA TGCAAGTAGC GTATGCGCTC
6481 ACGCAACTGG TCCAGAACCT TGACCGAACG CAGCGGTGGT AACGGCGCAG TGGCGGTTTT
6541 CATGGCTTGT TATGACTGTT TTTTTGGGGT ACAGTCTATG CCTCGGGCAT CCAAGCAGCA
6601 AGCGCGTTAC GCCGTGGGTC GATGTTTGAT GTTATGGAGC AGCAACGATG TTACGCAGCA
6661 GGGCAGTCGC CCTAAAACAA AGTTAAACAT CATGAGGGAA GCGGTGATCG CCGAAGTATC
6721 GACTCAACTA TCAGAGGTAG TTGGCGTCAT CGAGCGCCAT CTCGAACCGA CGTTGCTGGC
6781 CGTACATTTG TACGGCTCCG CAGTGGATGG CGGCCTGAAG CCACACAGTG ATATTGATTT
6841 GCTGGTTACG GTGACCGTAA GGCTTGATGA AACAACGCGG CGAGCTTTGA TCAACGACCT
6901 TTTGGAAACT TCGGCTTCCC CTGGAGAGAG CGAGATTCTC CGCGCTGTAG AAGTCACCAT
6961 TGTTGTGCAC GACGACATCA TTCCGTGGCG TTATCCAGCT AAGCGCGAAC TGCAATTTGG
7021 AGAATGGCAG CGCAATGACA TTCTTGCAGG TATCTTCGAG CCAGCCACGA TCGACATTGA
7081 TCTGGCTATC TTGCTGACAA AAGCAAGAGA ACATAGCGTT GCCTTGGTAG GTCCAGCGGC
7141 GGAGGAACTC TTTGATCCGG TTCCTGAACA GGATCTATTT GAGGCGCTAA ATGAAACCTT
7201 AACGCTATGG AACTCGCCGC CCGACTGGGC TGGCGATGAG CGAAATGTAG TGCTTACGTT
7261 GTCCCGCATT TGGTACAGCG CAGTAACCGG CAAAATCGCG CCGAAGGATG TCGCTGCCGA
7321 CTGGGCAATG GAGCGCCTGC CGGCCCAGTA TCAGCCCGTC ATACTTGAAG CTAGACAGGC
7381 TTATCTTGGA CAAGAAGAAG ATCGCTTGGC CTCGCGCGCA GATCAGTTGG AAGAATTTGT
7441 CCACTACGTG AAAGGCGAGA TCACCAAGGT AGTCGGCAAA TAATGTCTAA CAATTCGTTC
7501 AAGCCGACGC CGCTTCGCGG CGCGGCTTAA CTCAAGCGTT AGATGCACTA AGCACATAAT
7561 TGCTCACAGC CAAACTATCA GGTCAAGTCT GCTTTTATTA TTTTTAAGCG TGCATAATAA
7621 GCCCTACACA AATTGGGAGA TATATCATGA AAGGCTGGCT TTTTCTTGTT ATCGCAATAG
7681 TTGGCGAAGT AATCGCAACA T
//
```

poRFP-oGFP

```
LOCUS       Exported             5659 bp ds-DNA     circular SYN 18-JUN-2019
DEFINITION  synthetic circular DNA
ACCESSION   .
VERSION     .
KEYWORDS    .
SOURCE      synthetic DNA construct
  ORGANISM  synthetic DNA construct
REFERENCE   1  (bases 1 to 5659)
AUTHORS   Alexander S. Mankin
TITLE     Direct Submission
  JOURNAL   Exported Tuesday, Jun 18, 2019 from SnapGene 4.3.10
          https://www.snapgene.com
FEATURES             Location/Qualifiers
     source          1..5659
                     /organism="synthetic DNA construct"
                     /mol_type="other DNA"
     promoter        66..110
                     /locus_tag="Plpp5"
                     /label=Plpp5
     protein_bind    111..135
                     /label=lac operator
                     /bound_moiety="lac repressor encoded by lacI"
                     /note="The lac repressor binds to the lac operator to
                     inhibit transcription in E. coli. This inhibition can be
                     relieved by adding lactose or
                     isopropyl-beta-D-thiogalactopyranoside (IPTG)."
     RBS             153..159
                     /locus_tag="oRBS(1)"
                     /label=oRBS
                     /label=oRBS(1)
     CDS             166..888
                     /locus_tag="oGFP"
                     /label=oGFP
     terminator      1128..1214
                     /gene="Escherichia coli rrnB"
                     /label=rrnB T1 terminator
                     /note="transcription terminator T1 from the E. coli rrnB
                     gene"
     terminator      1306..1333
                     /label=rrnB T2 terminator
                     /note="transcription terminator T2 from the E. coli rrnB
                     gene"
     rep_origin      1919..2464
                     /direction=RIGHT
                     /label=p15A ori
                     /note="Plasmids containing the medium-copy-number p15A
                     origin of replication can be propagated in E. coli cells
                     that contain a second plasmid with the ColE1 origin."
     promoter        3074..3118
                     /label=T5 promoter
                     /note="bacteriophage T5 promoter for E. coli RNA
                     polymerase, with embedded lac operator"
     -35_signal      3089..3093
```

```
                     /locus_tag="-35 signal"
                     /label=-35 signal
     protein_bind    3094..3110
                     /label=lac operator
                     /bound_moiety="lac repressor encoded by lacI"
                     /note="The lac repressor binds to the lac operator to
                     inhibit transcription in E. coli. This inhibition can be
                     relieved by adding lactose or
                     isopropyl-beta-D-thiogalactopyranoside (IPTG)."
     -10_signal      3110..3115
                     /locus_tag="-10 signal"
                     /label=-10 signal
     protein_bind    3126..3142
                     /label=lac operator
                     /bound_moiety="lac repressor encoded by lacI"
                     /note="The lac repressor binds to the lac operator to
                     inhibit transcription in E. coli. This inhibition can be
                     relieved by adding lactose or
                     isopropyl-beta-D-thiogalactopyranoside (IPTG)."
     RBS             3165..3171
                     /locus_tag="oRBS"
                     /label=oRBS
     CDS             3180..3857
                     /codon_start=1
                     /product="monomeric derivative of DsRed (Campbell et al.,
                     2002)"
                     /label=mRFP1
                     /translation="MASSEDVIKEFMRFKVRMEGSVNGHEFEIEGEGEGRPYEGTQTAK
                     LKVTKGGPLPFAWDILSPQFQYGSKAYVKHPADIPDYLKLSFPEGFKWERVMNFEDGGV
                     VTVTQDSSLQDGEFIYKVKLRGTNFPSDGPVMQKKTMGWEASTERMYPEDGALKGEIKM
                     RLKLKDGGHYDAEVKTTYMAKKPVQLPGAYKTDIKLDITSHNEDYTIVEQYERAEGRHS
                     TGA"
     terminator      3921..4015
                     /label=lambda t0 terminator
                     /note="transcription terminator from phage lambda"
     CDS             4650..5441
                     /codon_start=1
                     /gene="aadA"
                     /product="aminoglycoside adenylyltransferase (Murphy,
                     1985)"
                     /label=SmR
                     /note="confers resistance to spectinomycin and
                     streptomycin"
                     /translation="MREAVIAEVSTQLSEVVGVIERHLEPTLLAVHLYGSAVDGGLKPH
                     SDIDLLVTVTVRLDETTRRALINDLLETSASPGESEILRAVEVTIVVHDDIIPWRYPAK
                     RELQFGEWQRNDILAGIFEPATIDIDLAILLTKAREHSVALVGPAAEELFDPVPEQDLF
                     EALNETLTLWNSPPDWAGDERNVVLTLSRIWYSAVTGKIAPKDVAADWAMERLPAQYQP
                     VILEARQAYLGQEEDRLASRADQLEEFVHYVKGEITKVVGK"
ORIGIN

        1 CCGCATTAAA ATCTAGCGAG GATCCGAGGC GCGCCCAGAG ATTTTGAGAC ACAACGTGGC
       61 TTTCCATCAA AAAAATATTG ACAACATAAA AAACTTTGTG TTATACTTGT GGAATTGTGA
      121 GCGGATAACA ATTCTATATC TGTTATTTTT TCCAACCACA GATCTATGAG CAAAGGTGAA
      181 GAACTGTTTA CCGGCGTTGT GCCGATTCTG GTGGAACTGG ATGGCGATGT GAACGGTCAC
      241 AAATTCAGCG TGCGTGGTGA AGGTGAAGGC GATGCCACGA TTGGCAAACT GACGCTGAAA
      301 TTTATCTGCA CCACCGGCAA ACTGCCGGTG CCGTGGCCGA CGCTGGTGAC CACCCTGACC
```

```
 361 TATGGCGTTC AGTGTTTTAG TCGCTATCCG GATCACATGA AACGTCACGA TTTCTTTAAA
 421 TCTGCAATGC CGGAAGGCTA TGTGCAGGAA CGTACGATTA GCTTTAAAGA TGATGGCAAA
 481 TATAAAACGC GCGCCGTTGT GAAATTTGAA GGCGATACCC TGGTGAACCG CATTGAACTG
 541 AAAGGCACGG ATTTTAAAGA AGATGGCAAT ATCCTGGGCC ATAAACTGGA ATACAACTTT
 601 AATAGCCATA ATGTTTATAT TACGGCGGAT AAACAGAAAA ATGGCATCAA AGCGAATTTT
 661 ACCGTTCGCC ATAACGTTGA AGATGGCAGT GTGCAGCTGG CAGATCATTA TCAGCAGAAT
 721 ACCCCGATTG GTGATGGTCC GGTGCTGCTG CCGGATAATC ATTATCTGAG CACGCAGACC
 781 GTTCTGTCTA AAGATCCGAA CGAAAAAGGC ACGCGGGACC ACATGGTTCT GCACGAATAT
 841 GTGAATGCGG CAGGTATTAC GTGGAGCCAT CCGCAGTTCG AAAAATAAGT CGACCGGCTG
 901 CTAACAAAGC CCGCGGCCGC TGAAGATCGA TCTCGACGAG TGAGAGAAGA TTTTCAGCCT
 961 GATACAGATT AAATCAGAAC GCAGAAGCGG TCTGATAAAA CAGAATTTGC CTGGCGGCAG
1021 TAGCGCGGTG GTCCCACCTG ACCCCATGCC GAACTCAGAA GTGAAACGCC GTAGCGCCGA
1081 TGGTAGTGTG GGGTCACCCC ATGCGAGAGT AGGGAACTGC CAGGCATCAA ATAAAACGAA
1141 AGGCTCAGTC GAAAGACTGG GCCTTTCGTT TTATCTGTTG TTTGTCGGTG AACGCTCTCC
1201 TGAGTAGGAC AAATCCGCCG GGAGCGGATT TGAACGTTGC GAAGCAACGG CCCGGAGGGT
1261 GGCGGGCAGG ACGCCCGCCA TAAACTGCCA GGCATCAAAT TAAGCAGAAG GCCATCCTGA
1321 CGGATGGCCT TTTTGCGTTT CTACACTCGA GGATCTCTTT CCTTTCCATC AAAAAAATAT
1381 TGATGAAATG AGCTGTGGGG CGTTATTTAG GTTTTTTCTT CTTTCGAAAA AATCTTTCTT
1441 TATGAAGTTA AAAGCTATGT ATTCAATAGC ATATTTTGAA TATGGACATA GAATAGTGCT
1501 TATCACTATT GCATATAGCA TCTTATCTGA CACAAGGAAA TAATACCCTT CGCTGTTTTT
1561 TGTTATAAGG TATATATATA TAAGTGTGCA GTACAGGCCA AATAAAATAT TTTTTATGTA
1621 GTATCTTAAA TCCCGCAAGA GGCCCGGCAG TACCGGCATA ACCAAGCCTA TGCCTACAGC
1681 ATCCAGGGTG ACGGTGCCGA GGATGACGAT GAGCGCATTG TTAGATTTCA TACACGGTGC
1741 CTGACTGCGT TAGCAATTTA ACTGTGATAA ACTACCGCAT TAAAGCTTAT CGATGATAAG
1801 CTGTCAAACA TGAGAATTAC AACTTATATC GTATGGGGCT GACTTCAGGT GCTACATTTG
1861 AAGAGATAAA TTGCACTGAA ATCTAGAAAT ATTTTATCTG ATTAATAAGA TGATCTTCTT
1921 GAGATCGTTT TGGTCTGCGC GTAATCTCTT GCTCTGAAAA CGAAAAAACC GCCTTGCAGG
1981 GCGGTTTTTC GAAGGTTCTC TGAGCTACCA ACTCTTTGAA CCGAGGTAAC TGGCTTGGAG
2041 GAGCGCAGTC ACCAAAACTT GTCCTTTCAG TTTAGCCTTA ACCGGCGCAT GACTTCAAGA
2101 CTAACTCCTC TAAATCAATT ACCAGTGGCT GCTGCCAGTG GTGCTTTTGC ATGTCTTTCC
2161 GGGTTGGACT CAAGACGATA GTTACCGGAT AAGGCGCAGC GGTCGGACTG AACGGGGGGT
2221 TCGTGCATAC AGTCCAGCTT GGAGCGAACT GCCTACCCGG AACTGAGTGT CAGGCGTGGA
2281 ATGAGACAAA CGCGGCCATA ACAGCGGAAT GACACCGGTA AACCGAAAGG CAGGAACAGG
2341 AGAGCGCACG AGGGAGCCGC CAGGGGGAAA CGCCTGGTAT CTTTATAGTC CTGTCGGGTT
2401 TCGCCACCAC TGATTTGAGC GTCAGATTTC GTGATGCTTG TCAGGGGGGC GGAGCCTATG
2461 GAAAAACGGC TTTGCCGCGG CCCTCTCACT TCCCTGTTAA GTATCTTCCT GGCATCTTCC
2521 AGGAAATCTC CGCCCCGTTC GTAAGCCATT TCCGCTCGCC GCAGTCGAAC GACCGAGCGT
2581 AGCGAGTCAG TGAGCGAGGA AGCGGAATAT ATCCTGTATC ACATATTCTG CTGACGCACC
2641 GGTGCAGCCT TTTTTCTCCT GCCACATGAA GCACTTCACT GACACCCTCA TCAGTGCCAA
2701 CATAGTAAGC CAGTATACAC TCCGCTAGCG CTGATGTCCG GCGGTGCTTT TGCCGTTACG
2761 CACCACCCCG TCAGTAGCTG AACAGGAGGG ACAGAGCTTT ATGCTTGTAA ACCGTTTTGT
2821 GAAAAAATTT TTAAAATAAA AAAGGGGACC TCTAGGGTCC CCAATTAATT AGTAATATAA
2881 TCTATTAAAG GTCATTCAAA AGGTCATCCA CCGGATCAAT TCCCCTGCTC GCGCAGGCTG
2941 GGTGCCAAGC TCTCGGGTAA CATCAAGGCC CGATCCTTGG AGCCCTTGCC CTCCCGCACG
3001 ATGATCGTGC CGTGATCGAA AATCCAGATC CTTGACCCGC ATTTGCAAAC CCTCACTGAT
3061 CCGCATGGGG CCCTCATAAA AAATTTATTT GCTTTGTGAG CGGATAACAA TTATAATAGA
3121 TTCAATTGTG AGCGGATAAC AATTTCACAC AGAATTCATT AAAGCAACCA CAATTAACTA
3181 TGGCTTCCTC CGAAGACGTT ATCAAAGAGT TCATGCGTTT CAAAGTTCGT ATGGAAGGTT
3241 CCGTTAACGG TCACGAGTTC GAAATCGAAG GTGAAGGTGA AGGTCGTCCG TACGAAGGTA
3301 CCCAGACCGC TAAACTGAAA GTTACCAAAG GTGGTCCGCT GCCGTTCGCT TGGGACATCC
3361 TGTCCCCGCA GTTCCAGTAC GGTTCCAAAG CTTACGTTAA ACACCCGGCT GACATCCCGG
3421 ACTACCTGAA ACTGTCCTTC CCGGAAGGTT TCAAATGGGA ACGTGTTATG AACTTCGAAG
3481 ACGGTGGTGT TGTTACCGTT ACCCAGGACT CCTCCCTGCA AGACGGTGAG TTCATCTACA
3541 AAGTTAAACT GCGTGGTACC AACTTCCCGT CCGACGGTCC GGTTATGCAG AAAAAAACCA
3601 TGGGTTGGGA AGCTTCCACC GAACGTATGT ACCCGGAAGA CGGTGCTCTG AAAGGTGAAA
3661 TCAAAATGCG TCTGAAACTG AAAGACGGTG GTCACTACGA CGCTGAAGTT AAAACCACCT
3721 ACATGGCTAA AAAACCGGTT CAGCTGCCGG GTGCTTACAA AACCGACATC AAACTGGACA
```

```
3781 TCACCTCCCA CAACGAAGAC TACACCATCG TTGAACAGTA CGAACGTGCT GAAGGTCGTC
3841 ACTCCACCGG TGCTTAAGGA TCCGCATGCG AGCTCGGTAC CCCGGGTCGA CCTGCAGCCA
3901 AGCTTAATTA GCTGAGCTTG GACTCCTGTT GATAGATCCA GTAATGACCT CAGAACTCCA
3961 TCTGGATTTG TTCAGAACGC TCGGTTGCCG CCGGGCGTTT TTTATTGGTG AGAATCCAAG
4021 CTAGCTTGGC GAGATTTTCA GGAGCTAAGG AAGCTAAAGA TCCGACATAT GCCGTTCCAT
4081 ACAGAAGCTG GGCGAACAAA CGATGCTCGC CTTCCAGAAA ACCGAGGATG CGAACCACTT
4141 CATCCGGGGT CAGCACCACC GGCAAGCGCC GCGACGGCCG AGGTCTTCCG ATCTCCTGAA
4201 GCCAGGGCAG ATCCGTGCAC AGCACCTTGC CGTAGAAGAA CAGCAAGGCC GCCAATGCCT
4261 GACGATGCGT GGAGACCGAA ACCTTGCGCT CGTTCGCCAG CCAGGACAGA AATGCCTCGA
4321 CTTCGCTGCT GCCCAAGGTT GCCGGGTGAC GCACACCGTG GAAACGGATG AAGGCACGAA
4381 CCCAGTGGAC ATAAGCCTGT TCGGTTCGTA AGCTGTAATG CAAGTAGCGT ATGCGCTCAC
4441 GCAACTGGTC CAGAACCTTG ACCGAACGCA GCGGTGGTAA CGGCGCAGTG GCGGTTTTCA
4501 TGGCTTGTTA TGACTGTTTT TTTGGGGTAC AGTCTATGCC TCGGGCATCC AAGCAGCAAG
4561 CGCGTTACGC CGTGGGTCGA TGTTTGATGT TATGGAGCAG CAACGATGTT ACGCAGCAGG
4621 GCAGTCGCCC TAAAACAAAG TTAAACATCA TGAGGGAAGC GGTGATCGCC GAAGTATCGA
4681 CTCAACTATC AGAGGTAGTT GGCGTCATCG AGCGCCATCT CGAACCGACG TTGCTGGCCG
4741 TACATTTGTA CGGCTCCGCA GTGGATGGCG GCCTGAAGCC ACACAGTGAT ATTGATTTGC
4801 TGGTTACGGT GACCGTAAGG CTTGATGAAA CAACGCGGCG AGCTTTGATC AACGACCTTT
4861 TGGAAACTTC GGCTTCCCCT GGAGAGAGCG AGATTCTCCG CGCTGTAGAA GTCACCATTG
4921 TTGTGCACGA CGACATCATT CCGTGGCGTT ATCCAGCTAA GCGCGAACTG CAATTTGGAG
4981 AATGGCAGCG CAATGACATT CTTGCAGGTA TCTTCGAGCC AGCCACGATC GACATTGATC
5041 TGGCTATCTT GCTGACAAAA GCAAGAGAAC ATAGCGTTGC CTTGGTAGGT CCAGCGGCGG
5101 AGGAACTCTT TGATCCGGTT CCTGAACAGG ATCTATTTGA GGCGCTAAAT GAAACCTTAA
5161 CGCTATGGAA CTCGCCGCCC GACTGGGCTG GCGATGAGCG AAATGTAGTG CTTACGTTGT
5221 CCCGCATTTG GTACAGCGCA GTAACCGGCA AAATCGCGCC GAAGGATGTC GCTGCCGACT
5281 GGGCAATGGA GCGCCTGCCG GCCCAGTATC AGCCCGTCAT ACTTGAAGCT AGACAGGCTT
5341 ATCTTGGACA AGAAGAAGAT CGCTTGGCCT CGCGCGCAGA TCAGTTGGAA GAATTTGTCC
5401 ACTACGTGAA AGGCGAGATC ACCAAGGTAG TCGGCAAATA ATGTCTAACA ATTCGTTCAA
5461 GCCGACGCCG CTTCGCGGCG CGGCTTAACT CAAGCGTTAG ATGCACTAAG CACATAATTG
5521 CTCACAGCCA AACTATCAGG TCAAGTCTGC TTTTATTATT TTTAAGCGTG CATAATAAGC
5581 CCTACACAAA TTGGGAGATA TATCATGAAA GGCTGGCTTT TTCTTGTTAT CGCAATAGTT
5641 GGCGAAGTAA TCGCAACAT
//
```

<u>GFP-TnaC template for *in vitro* translation</u>

```
LOCUS       Exported              1020 bp ds-DNA     linear   UNA 19-JUN-2019
DEFINITION  natural linear DNA
ACCESSION   .
VERSION     .
KEYWORDS    .
SOURCE      natural DNA sequence
  ORGANISM  unspecified
REFERENCE   1  (bases 1 to 1020)
AUTHORS   Alexander S. Mankin
TITLE     Direct Submission
  JOURNAL   Exported Wednesday, Jun 19, 2019 from SnapGene 4.3.10
            https://www.snapgene.com
FEATURES             Location/Qualifiers
     source          1..1020
                     /organism="unspecified"
                     /mol_type="genomic DNA"
     promoter        71..89
                     /label=T7 promoter
                     /note="promoter for bacteriophage T7 RNA polymerase"
     RBS             121..143
                     /note="efficient ribosome binding site from bacteriophage
                     T7 gene 10 (Olins and Rangwala, 1989)"
     CDS             151..855
                     /locus_tag="sfGFP"
                     /label=sfGFP
     CDS             856..900
                     /locus_tag="TnaC"
                     /label=TnaC
ORIGIN
        1 TCGATTTTTG TGATGCTCGT CAGGGGGGCG GAGCCTATGG AAACGAATTC AGATCTCGAT
       61 CCCGCGAAAT TAATACGACT CACTATAGGG AGACCACAAC GGTTTCCCTC TAGAAATAAT
      121 TTTGTTTAAC TTTAAGAAGG AGATATACAT ATGAGCAAAG GTGAAGAACT GTTTACCGGC
      181 GTTGTGCCGA TTCTGGTGGA ACTGGATGGC GATGTGAACG GTCACAAATT CAGCGTGCGT
      241 GGTGAAGGTG AAGGCGATGC CACGATTGGC AAACTGACGC TGAAATTTAT CTGCACCACC
      301 GGCAAACTGC CGGTGCCGTG GCCGACGCTG GTGACCACCC TGACCTATGG CGTTCAGTGT
      361 TTTAGTCGCT ATCCGGATCA CATGAAACGT CACGATTTCT TTAAATCTGC AATGCCGGAA
      421 GGCTATGTGC AGGAACGTAC GATTAGCTTT AAAGATGATG GCAAATATAA AACGCGCGCC
      481 GTTGTGAAAT TTGAAGGCGA TACCCTGGTG AACCGCATTG AACTGAAAGG CACGGATTTT
      541 AAAGAAGATG GCAATATCCT GGGCCATAAA CTGGAATACA ACTTTAATAG CCATAATGTT
      601 TATATTACGG CGGATAAACA GAAAAATGGC ATCAAAGCGA ATTTTACCGT TCGCCATAAC
      661 GTTGAAGATG GCAGTGTGCA GCTGGCAGAT CATTATCAGC AGAATACCCC GATTGGTGAT
      721 GGTCCGGTGC TGCTGCCGGA TAATCATTAT CTGAGCACGC AGACCGTTCT GTCTAAAGAT
      781 CCGAACGAAA AAGGCACGCG GGACCACATG GTTCTGCACG AATATGTGAA TGCGGCAGGT
      841 ATTACGTGGA GCCATAAATG GTTCAATATT GACAACAAAA TTGTCGATCA CCGCCCTTGA
      901 TTTGCCCTTC TGTAGCCATC ACCAGAGCCA AACCGATTAG ATTCAATGTG ATCTATTTGT
      961 TTGCTATATC TTAATTTTGC CTTTTGCAAA GGTCATCTCT CGTTTATTTA CTTGTTTTAG
//
```

GFP-TnaC (W12R) template for *in vitro* translation

```
LOCUS       Exported              1020 bp ds-DNA     linear   UNA 19-JUN-2019
DEFINITION  natural linear DNA
ACCESSION   .
VERSION     .
KEYWORDS    .
SOURCE      natural DNA sequence
  ORGANISM  unspecified
REFERENCE   1  (bases 1 to 1020)
AUTHORS   Alexander S. Mankin
TITLE     Direct Submission
  JOURNAL   Exported Wednesday, Jun 19, 2019 from SnapGene 4.3.10
            https://www.snapgene.com
FEATURES             Location/Qualifiers
     source          1..1020
                     /organism="unspecified"
                     /mol_type="genomic DNA"
     promoter        71..89
                     /label=T7 promoter
                     /note="promoter for bacteriophage T7 RNA polymerase"
     RBS             121..143
                     /note="efficient ribosome binding site from bacteriophage
                     T7 gene 10 (Olins and Rangwala, 1989)"
     CDS             151..855
                     /locus_tag="sfGFP"
                     /label=sfGFP
     CDS             856..900
                     /locus_tag="TnaC W12R"
                     /label=TnaC W12R
ORIGIN

        1 TCGATTTTTG TGATGCTCGT CAGGGGGGCG GAGCCTATGG AAACGAATTC AGATCTCGAT
       61 CCCGCGAAAT TAATACGACT CACTATAGGG AGACCACAAC GGTTTCCCTC TAGAAATAAT
      121 TTTGTTTAAC TTTAAGAAGG AGATATACAT ATGAGCAAAG GTGAAGAACT GTTTACCGGC
      181 GTTGTGCCGA TTCTGGTGGA ACTGGATGGC GATGTGAACG GTCACAAATT CAGCGTGCGT
      241 GGTGAAGGTG AAGGCGATGC CACGATTGGC AAACTGACGC TGAAATTTAT CTGCACCACC
      301 GGCAAACTGC CGGTGCCGTG GCCGACGCTG GTGACCACCC TGACCTATGG CGTTCAGTGT
      361 TTTAGTCGCT ATCCGGATCA CATGAAACGT CACGATTTCT TTAAATCTGC AATGCCGGAA
      421 GGCTATGTGC AGGAACGTAC GATTAGCTTT AAAGATGATG GCAAATATAA AACGCGCGCC
      481 GTTGTGAAAT TTGAAGGCGA TACCCTGGTG AACCGCATTG AACTGAAAGG CACGGATTTT
      541 AAAGAAGATG GCAATATCCT GGGCCATAAA CTGGAATACA ACTTTAATAG CCATAATGTT
      601 TATATTACGG CGGATAAACA GAAAAATGGC ATCAAAGCGA ATTTTACCGT TCGCCATAAC
      661 GTTGAAGATG GCAGTGTGCA GCTGGCAGAT CATTATCAGC AGAATACCCC GATTGGTGAT
      721 GGTCCGGTGC TGCTGCCGGA TAATCATTAT CTGAGCACGC AGACCGTTCT GTCTAAAGAT
      781 CCGAACGAAA AAGGCACGCG GGACCACATG GTTCTGCACG AATATGTGAA TGCGGCAGGT
      841 ATTACGTGGA GCCATAAACG GTTCAATATT GACAACAAAA TTGTCGATCA CCGCCCTTGA
      901 TTTGCCCTTC TGTAGCCATC ACCAGAGCCA AACCGATTAG ATTCAATGTG ATCTATTTGT
      961 TTGCTATATC TTAATTTTGC CTTTTGCAAA GGTCATCTCT CGTTTATTTA CTTGTTTTAG
//
```
