# Supplementary material for: A fully orthogonal system for protein synthesis in bacterial cells
Source: Nat Commun. 2020 Apr 20;11:1858. doi: 10.1038/s41467-020-15756-1 (PMC7170887; doi:10.1038/s41467-020-15756-1)
Supplement: Supplementary file 4 — Description of Additional Supplementary Files [file 41467_2020_15756_MOESM4_ESM.pdf]

Title: Supplementary Data 1:

Description: Fully annotated sequences of the plasmids pRibo-Tt, poRbs, poGFP2, poLuc2, poRFP-oGFP used in the study as well as the sequences of GFP-TnaC and GFPTnaC(W12R) templates used in cell free translation experiments.
