# Supplementary material for: A fully orthogonal system for protein synthesis in bacterial cells
Source: Nat Commun. 2020 Apr 20;11:1858. doi: 10.1038/s41467-020-15756-1 (PMC7170887; doi:10.1038/s41467-020-15756-1)
Supplement: Supplementary file 5 — Peer Review File [file 41467_2020_15756_MOESM5_ESM.pdf]

Editorial Note: This manuscript has been previously reviewed at another journal that is not operating a transparent peer review scheme. This document only contains reviewer comments and rebuttal letters for versions considered at *Nature Communications*. Mentions of prior referee reports have been redacted.
